# Psychometric validation of the Chronic Ocular Pain Questionnaire (COP-Q)

**DOI:** 10.1186/s41687-025-00862-9

**Published:** 2025-03-12

**Authors:** Amy Findley, Brigitte J. Sloesen, Nicola Hodson, Agkreta Leventi, Ben Pascoe, Rob Arbuckle, Paul O’Brien, Christel Naujoks, Michela Montecchi-Palmer, Diana Plaza, Paul M. Karpecki, Pedram Hamrah

**Affiliations:** 1 Adelphi Values Ltd, Patient-Centered Outcomes, Bollington, Cheshire, UK; 2 Novartis Pharma AG, Basel, Switzerland; 3 Kentucky Eye Institute and University of Pikeville Kentucky College of Optometry, Pikeville, KY USA; 4 Department of Ophthalmology, Cornea Service, New England Eye Center and Tufts Medical Center, Tufts University School of Medicine, Boston, MA USA

**Keywords:** Chronic ocular surface pain, Patient-reported outcome, Clinical outcome assessment, Psychometric validation, Observational

## Abstract

**Background:**

The Chronic Ocular Pain Questionnaire (COP-Q) is a newly developed patient-reported outcome (PRO) measure intended to assess symptoms and impacts associated with Chronic Ocular Surface Pain (COSP). This study assessed the psychometric properties of the COP-Q to determine the adequacy of the COP-Q as a ‘fit-for-purpose’ instrument to derive trial endpoints for future clinical studies in COSP.

**Methods:**

Patients with COSP completed the COP-Q daily for four weeks on an electronic, touch-screen, tablet device as part of a longitudinal, observational study in the United States (*N* = 124). Analyses were conducted to assess item properties, dimensionality and scoring, reliability and validity, and interpretation of scores. In addition, 4-hour and 24-hour recall period versions of the COP-Q Symptom Module were compared.

**Results:**

Item responses were distributed across the full response scale for most COP-Q items. Inter-item correlations did not identify any redundant items (*r* > 0.90) and all items correlated at > 0.40 in their respective module. Confirmatory factor analysis (CFA) provided acceptable support for the unidimensional structure of the multi-item scales in the COP-Q and calculation of a total score for each module. However, CFA and Rasch analysis outlined potential redundant items for the COP-Q Visual Tasking Module (VTM), which were removed, resulting in a six-item VTM. The multi-item COP-Q modules had excellent internal consistency (α range = 0.91–0.96) and suggested fair to excellent test-retest reliability (ICC/Kappa range = 0.651–0.940) for all COP-Q modules. Construct validity for all COP-Q modules was supported by a logical pattern of correlations with concurrent measures and evidence of ability to distinguish between known-groups, with statistically significant differences between COSP severity groups. Paired t-tests, coefficient of determination (CoD) and concordance correlation coefficients (CCC) showed statistically significant differences between the two recall period versions of the Symptom Module, although the magnitude of the difference was small, and each version shares a high level of reproducibility in scores.

**Conclusions:**

Findings provide evidence that the COP-Q is a valid and reliable measure of patient-reported COSP symptoms and impacts for use in future clinical trials in COSP. Both 4-hour and 24-hour Symptom Module recall period versions are likely to yield consistent results and are equally robust.

**Supplementary Information:**

The online version contains supplementary material available at 10.1186/s41687-025-00862-9.

## Background

Ocular pain is a secondary symptom in various ophthalmic conditions, and can also be a consequence of recent eye surgery, eye trauma or postulated mechanisms such as inflammation and sensory neuronal dysregulation [1, 2]. While ocular pain from such events is common, Chronic Ocular Surface Pain (COSP) manifests as persistent pain-related symptoms that are disproportionate to expected clinical signs, or experienced for longer than clinically anticipated, even when other signs of trauma or surgery have healed [1, 2]. COSP is therefore defined as persistent pain at the ocular surface lasting for more than three months in the absence of other tissue injury [1, 3, 4], and can include nociceptive, inflammatory and neuropathic pain [4]. These pain types are not mutually exclusive and can occur in the same patient. Diagnosis is challenging with currently no universal diagnostic criteria for COSP [1, 5]. Consequently, definitive incidence and prevalence data are lacking [3].

Beyond pain, COSP symptoms may include sensations of dryness, burning, irritation, foreign body sensation, light sensitivity and itch at the ocular surface [2, 6–11]. These symptoms can cause difficulties with daily tasks that rely on vision, such as using digital devices, reading, and driving – all of which can significantly impact on physical, emotional, and social domains of Health-Related Quality of Life (HRQoL) [12–15]. There is little research exploring COSP as a distinct condition in patients who experience various types of ocular surface pain. Available treatment options include the use of artificial tears, anti-inflammatory treatments, autologous serum tears, neurostimulation therapies, and systemic treatments, in addition to treatments for underlying conditions associated with COSP. However, there is limited evidence regarding the extent to which such treatments alleviate the symptoms associated with COSP [1, 6, 16]. No treatments have yet been approved specifically for COSP, suggesting high unmet need.

Given that the symptoms of COSP can be disproportionate to its observable clinical signs (as can be seen in cases of neuropathic pain) [1, 2], patient-reported outcome (PRO) measures are highly important for measuring COSP symptoms, functional impairment, and wider HRQoL impacts, to support clinical trial endpoints and evaluation of new therapies. Although a review of the literature identified a number of existing PRO instruments that have been used in ocular pain [15, 17–24], none of these instruments were considered optimal for use in planned clinical trials in COSP to assess the target measurement concepts of interest [15]. As a result, the Chronic Ocular Pain Questionnaire (COP-Q) was developed as a new, COSP-specific, PRO measure to assess symptoms and quality of life for COSP patients, including impacts on visual functioning, in line with regulatory and best practice guidelines for PRO development [25–29]. Development and evaluation of the content validity of the COP-Q was informed by qualitative concept elicitation (CE) and cognitive debriefing (CD) interviews with COSP patients with a range of etiologies [15]. In this paper we report on the first assessment of the psychometric properties of an electronic version (ePRO) of the COP-Q, performed on data from a longitudinal, observational study.

## Methods

### Study design

This was a non-interventional, observational study to evaluate the psychometric properties and finalize scoring (with consideration of item reduction) of the COP-Q. Study participants (*N* = 124) completed the COP-Q daily for four weeks (28 days) on an electronic, touch-screen, tablet device. The first ten participants enrolled also took part in a usability interview (*n* = 10) to ensure equivalency of the electronic version of the COP-Q to the paper version included in previous testing and confirmed that the electronic version was suitable for use in the observational study (see Supplementary File 1 for usability testing methods and results).

Additional measures were implemented to support psychometric analyses, including patient global impression of severity (PGI-S) and change (PGI-C) items, with categorical response options designed to capture participants’ perception of overall COSP severity (PGI-S) over the past 7 days and change in overall COSP severity (PGI-C) compared to the start of the study. These items were developed in line with US Food and Drug Administration (FDA) guidelines [26–29] to be used as anchors to support analysis of test-retest, known-groups validity, and estimation of meaningful change thresholds.

**Fig. 1:**
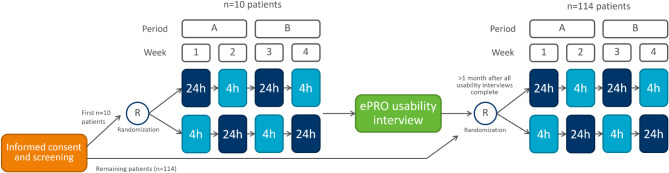
Data collection design for the COP-Q symptom module

Each week of the data collection period consisted of twice daily completion of the COP-Q, with the PGI items completed on the last day of each week (i.e., every seven days). During the qualitative development of the COP-Q (and taking account of regulatory feedback), both a 24-hour recall period version (administered once a day) and a 4-hour recall period version (administered twice a day; AM and PM) of the COP-Q Symptom Module were developed and tested. It was judged that both could be of value, depending on future clinical trial designs, and balancing considerations of minimizing risk of recall bias versus feasibility of multiple daily completions throughout a long clinical trial. Consequently, the observational study was designed with a cross-over design to support validation of both recall period versions of the Symptom Module. Participants completed the 24-hour and 4-hour recall versions of the COP-Q Symptom Module on alternate weeks, with the order randomized, to enable validation of and examination of the comparability of the two recall period versions (Fig. 1). The ophthalmology clinic participants were recruited from was not considered as part of this randomization, which was instead stratified by enrolment.

Participants also completed the Visual Functioning Questionnaire (VFQ-25) [24] and the EuroQoL-5 Dimension-5 Level (EQ-5D-5 L) with the vision bolt-on item on paper [30] at baseline, and the Ocular Pain Assessment Survey (OPAS) [17] on paper at the end of the diary completion period, for the purpose of supporting convergent validity analyses (i.e., correlation of the VFQ-25, EQ-5D-5 L and OPAS with the COP-Q was examined) (see Table 1 for schedule of assessments).

**Table 1:** Schedule of assessments

Assessment	Domain	Visit Name						
		Screening	Baseline	Week 1	Week 2	Week 3	Week 4	Final site visit
		Day − 1	Day 0	Days 1–7	Days 8–14	Days 15–21	Days 22–28	Days 29–35
CRF	All items	X (as close to baseline visit as possible)						
Demographic form	All items	X (as close to baseline visit as possible)						
VFQ-25	All items		X					
EQ-5D-5 L (with vision bolt-on)	All items		X					
COP-Q	Eye Pain Severity Module			X (twice daily, morning and evening assessments^1^)	X (twice daily, morning and evening assessments^1^)	X (twice daily, morning and evening assessments^1^)	X (twice daily, morning and evening assessments^1^)	
	Eye Pain Frequency Module			X (daily evening assessments^2^)	X (daily evening assessments^2^)	X (daily evening assessments^2^)	X (daily evening assessments^2^)	
	Symptom Module			X (daily, or twice daily, depending on study arm)	X (daily, or twice daily, depending on study arm)	X (daily, or twice daily, depending on study arm)	X (daily, or twice daily, depending on study arm)	
	VTM			X (assessments weekly^3^ Day 7)	X (assessments weekly^3^ Day 14)	X (assessments weekly^3^ Day 21)	X (assessments weekly^3^ Day 28)	
	HRQoL Module			X (assessments weekly^3^ Day 7)	X (assessments weekly^3^ Day 14)	X (assessments weekly^3^ Day 21)	X (assessments weekly^3^ Day 28)	
PGI items	PGI-S items			X (assessments weekly^3^ Day 7)	X (assessments weekly^3^ Day 14)	X (assessments weekly^3^ Day 21)	X (assessments weekly^3^ Day 28)	
	PGI-C items			X (assessments weekly^3^ Day 7)	X (assessments weekly^3^ Day 14)	X (assessments weekly^3^ Day 21)	X (assessments weekly^3^ Day 28)	
OPAS	All items							X

^1^Morning assessments completed between 07:00 to 10:00 (participants were encouraged by study staff to complete as close as possible to the same time each day)

^2^Evening assessments completed between 18:00 to 23:00 (participants were encouraged by study staff to complete as close as possible to the same time each day)

^3^Weekly assessments completed between 18:00 to 23:00

CRF = Case Report Form; VFQ-25 = Visual Functioning Questionnaire; EQ-5D-5 L = EuroQoL-5 Dimension-5 Level; COP-Q = Chronic Ocular Pain Questionnaire; VTM = Visual Tasking Module; HRQoL = health-related quality of life; PGI-S = Patient Global Impression of Severity; PGI-C = Patient Global Impression of Change; OPAS = Ocular Pain Assessment Survey

### Participant sample

Participants with symptoms of COSP (*N* = 124) were recruited by ophthalmologists from four specialist ophthalmology clinics in the United States (US). All participants were required to be 18 years or older, to have symptoms of COSP (chronic, persistent eye pain [can also be described as other symptoms e.g., burning, irritation, dryness etc.] at the ocular surface lasting for more than three months at screening), irrespective of treatment (further details on the eligibility criteria outlined in Supplementary File 2). In addition, it was required that their primary complaint was ocular pain coming from the surface of the eye [corneal or conjunctiva rather than systemic pain] and that they experienced ocular pain at least four days in a typical week. Individuals with an active ocular infection, or who experienced, or had a history of, acute seasonal ocular allergies during the time they would be participating in the study, were excluded from participating.

Sample quotas were employed to ensure a range of clinical and demographic characteristics were represented in the sample, including participants with a variety of etiologies for their COSP, such as eye surgery and comorbid conditions such as DED. These are also detailed in Supplementary File 2.

Two specialist ophthalmologists with expertise in COSP (authors PH and PK) helped interpret the results and contributed to decisions around item deletion and finalization of scoring. The same expert clinicians also provided extensive input regarding the selection/retention and wording of items in the previous qualitative research [15].

### Overview of instruments

#### Chronic Ocular Pain Questionnaire (COP-Q)

**Fig. 2:**
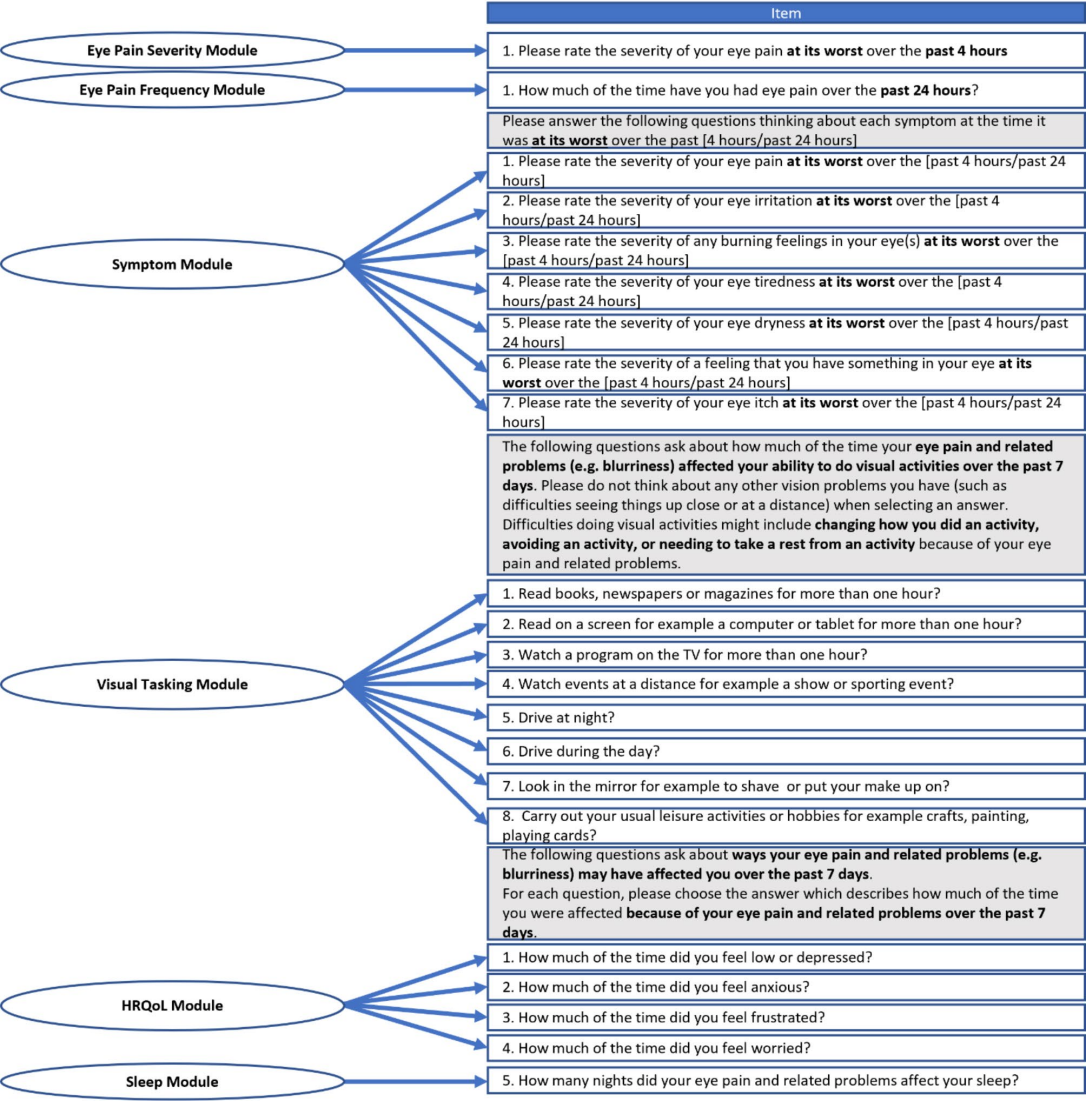
Hypothesized conceptual framework of the COP-Q

The initial version of the COP-Q administered to participants for psychometric validation consisted of five modules that assessed eye pain and related symptoms, ability to carry out visual activities, and HRQoL in COSP patients (Table 2). The hypothesized conceptual framework for the COP-Q (Fig. 2) was evaluated empirically during this study. An overview of the additional study instruments used for validation are presented in Supplementary File 3.

**Table 2:** Overview of the COP-Q modules

Module	Description	Response scale and scoring
Eye Pain Severity Module	A single item designed to capture participants’ perception of eye pain severity during the ‘past 4-hours’.	Numeric rating scale (NRS) from 0–10 ranging from ‘No eye pain’ (0) to ‘Worst possible eye pain’ (10).
Eye Pain Frequency Module	A single item designed to capture the frequency of participants’ eye pain within the ‘past 24-hours’.	Five-point verbal rating scale (VRS), ranging from ‘None of the time’ (0) to ‘All of the time’ (4).
Symptom Module	Seven items that assess symptoms associated with COSP. Two different recall period versions of the COP-Q Symptom Module were evaluated as part of this study (a 24-hour and a 4-hour recall period version).	All items have a 0–10 NRS, ranging from not experiencing the symptom at all (0) to experiencing the symptom at its worst (10) (e.g., the response scale for ‘eye irritation’ is ‘No eye irritation’ [0] to ‘Worst possible eye irritation’ [10]). A higher score indicates greater severity of that specific symptom. No items are reverse scored.
Visual Tasking Module (VTM)	Eight items that assess visual functioning in participants with COSP over the ‘past 7 days’.	Seven-point VRS, ranging from ‘None of the time’ (0) to ‘All of the time’ (6). Each item also has two additional response options ‘I avoided or was completely unable to do this activity due to my eye problems’ (7) and ‘I did not do this for reasons unrelated to my eye problems’ (not applicable). If these response options were selected, the participant’s item score was not included in the summary score and their item score was effectively treated as missing (but recorded separately). A higher score indicates greater impairment to visual activities. No items are reverse scored.
Health-Related Quality of Life (HRQoL) Module	Five items that assess quality of life/emotional wellbeing in participants with COSP over the ‘past 7 days’.	Five-point VRS, ranging from ‘None of the time’ (0) to ‘All of the time’ (4). The final item, that asks about how many nights participants’ eye pain and related problems affected their sleep over the past 7 days, has a five-point VRS, ranging from ‘0 nights’ (0) to ‘Every night’ (4). A higher score across all items indicates greater impact on HRQoL. No items are reverse scored.

NRS = numeric rating scale; VRS = verbal rating scale; VTM = Visual Tasking Module; HRQoL = health-related quality of life

### Statistical methods

A series of tests and analyses were performed throughout the study, each designed to evaluate different aspects of an item or score’s performance. Five stages of analysis were conducted corresponding to the assessment of item properties (Stage 1), dimensionality and scoring (Stage 2), score reliability and validity (Stage 3), interpretation of scores (Stage 4), and comparison of the 4-hour and 24-hour recall period versions of the Symptom Module (Stage 5). All planned statistical analyses were detailed in a psychometric analysis plan and were conducted using SAS^®^ version 9.4 or higher [31], Mplus [32] or R version 4.1.1 [33]. Table 3 details the main statistical methods used in this study. Analysis populations are detailed in Supplementary File 4. To note, due to the alternating study design (Fig. 1), different study weeks were used facilitate assessment of different recall periods for the ‘Symptom module’ (4-hour and 24-hour recall period). In addition, some study weeks were pooled to increase sample size and enable test-retest reliability analysis.

**Table 3:** Overview of statistical analyses

Analysis	Description and rationale
Stage 1: Item properties	
Quality of completion	• The quality of completion of the COP-Q was evaluated to identify any items with unexpectedly high levels of missing data. Missing data at the form-level and item-level were summarized at all time points in the full analysis population (i.e. all participants enrolled in the observational study).
Item response distributions and floor and ceiling effects	• The distributions of responses to each item were summarized through presenting the frequency and percentage of each endorsed response to help understand whether the COP-Q is able to capture the full range of COSP severities. • Response distributions were examined separately for morning and evening items for those items completed twice daily. ○ The second completion was used for all assessments in case the first completion was atypical due to participants being unfamiliar with completing the diary. • Percentages of minimum and maximum responses were also calculated to examine floor and ceiling effects for all items. An item with a substantial proportion of participants scoring floor/ceiling was flagged for further consideration: ○ Cut-off for ceiling and floor effects for the COP-Q Eye Pain Severity and Symptom Modules were defined as > 20%; ○ Cut-off for ceiling and floor effects for the VTM, were defined as > 30%; ○ Cut-off for ceiling and floor effects for the Eye Pain Frequency and HRQoL Modules, were defined as > 40%.
Stage 2: Dimensionality and scoring	
Inter-item correlations	• Inter-item correlations were computed between each pair of items, and examined separately for morning and evening items for those completed twice daily to aid understanding of the relationships among items. Items that correlated very highly with one another (*r* ≥ 0.90; indicating over 80% shared variance) were flagged for possible deletion due to potential item redundancy. • This was assessed at Week 1 (Day 2) for the Symptom modules (4 h and 24 h) and at Week 2 for the VTM and HRQoL.
Confirmatory Factor Analysis (CFA)	• CFA was performed separately for the VTM and HRQoL Modules in the psychometric analysis population (i.e. all participants enrolled into the study with at least one item completed on the COP-Q at any time point) using Week 2 data. • Multi-level CFA was performed on the Symptom Module using all item responses data at Week 1, with the clinic visit not included. • For each of these COP-Q modules, the model tested was one with a single factor hypothesized to be influencing all items in the module. • Factor analytic models employed a weighted least square mean and variance adjusted (WLSMV) estimator (as item responses are ordinal in nature), with theta parameterization for the VTM and HRQoL Module. For the Symptom Module a Maximum Likelihood Estimator (MLE) was used (due to the ‘continuous’ nature of the response options). • Model fit indices were used to assess model fit (CFI = Comparative Fit Index; TLI = Tucker Lewis Index; RMSEA = Root Mean Square Error of Approximation and SRMR = Standardizes Root Mean Square Residual) [34–36]. • Model fit indices were evaluated against the following desirable thresholds with the intended use to guide model fit assessment and not as strict cut-offs (CFI > 0.95, TLI > 0.95, RMSEA < 0.08 and SRMR < 0.05) [34–37]. • Deciding between a weighted or unweighted summary score was informed through comparison of constrained vs. unconstrained CFA models.
Rasch analysis	• Psychometric evaluation was carried out in the context of fitting Item Response Theory (IRT) Rating Scale (RSM) Rasch model for the VTM and HRQoL Module. • Rasch analysis was conducted for the COP-Q at Week 2 for the VTM and the HRQoL Module. • Item characteristic curves were used to assess probability of responses, weak or overlapping item response categories. • Person fit was evaluated through assessment of standardized fit residuals and number/proportion of participants with fit residuals outside of the range 0 ± 2.5 were summarized [38]. • Local dependency was assessed by Yens Q3 statistic with any residual correlation greater than the average residual correlation + 0.30 highlighting potential redundancy and interdependence [39, 40]. • Item fit was assessed by the infit mean square (MNSQ) and outfit MNSQ to highlight observed responses that deviate from the Rasch model expectation. Values between 0.5–1.5 indicate acceptable item fit and are productive for measurement [41]. • Item person maps were employed to flag overlapping items and any gaps in item location on the latent trait continuum. • IRT assessment has been used as supplementary to the Classical test theory analyses and to further inform the structure and the scoring of the VTM and HRQoL analyzed within the CFA.
Stage 3: Reliability and validity of scores	
Reliability	
Internal consistency reliability	• Internal consistency reliability, concerned with the homogeneity of items, was evaluated using Cronbach’s alpha coefficient (> 0.70 for good internal consistency) [42]. • Internal consistency was assessed at the second-timepoint participants completed the module (dependent on randomization).
Test-retest reliability (TRT)	• TRT is concerned with the degree to which scores are consistent between two time points, 14 days apart, in a subset of “stable” patients. Participants were considered ‘stable’ for a given concept if they showed no change on the corresponding PGI-S item between the two time points for that concept – further details regarding the specific PGI-S items and time points used are provided in Supplementary File 4. • Intra-class correlation coefficients (ICCs) were calculated. The following cut-offs were employed to interpret ICC values: values less than 0.50 were considered indicative of poor reliability; values between 0.50 and 0.75 indicated moderate reliability; values between 0.75 and 0.90 indicated good reliability; and values greater than 0.90 indicated excellent reliability [43].
Construct validity	
Convergent validity	• Convergent validity was evaluated by calculating Spearman’s correlations with the following concurrent measures: ○ VFQ-25 (Baseline) ○ EQ-5D-5 L with vision bolt-on (Baseline) ○ OPAS (Final visit) • For the scores that include daily or twice daily items, convergent validity was evaluated both for daily/twice daily scores, and also for scores based on a 7-day average. • Correlations between COP-Q scores and the scores assessing similar or related concepts were expected to have strong correlations (≥ 0.50) thereby demonstrating convergent validity, whereas scores assessing unrelated concepts were expected to show small (< 0.30) or negligible correlations, demonstrating discriminant validity [44].
Known-groups analysis	• Construct validity of scores was also assessed using the known-groups method [44]. Scores were compared between participants who differ on variables hypothesized to influence the construct of interest. • The known-groups were defined using the following measures: ○ COSP severity (clinician-rated) ○ COSP severity (patient-reported) ○ Diagnosis ○ PGI-S eye pain items ○ PGI-S visual activities item
Stage 4: Interpretation of scores	
Distribution-based methods	• The distributional properties of the scores were used to provide an indication of the amount of change that exceeds measurement error [25, 45]. • Both 0.5 of the standard deviation (SD) and the SEM were calculated at day 2 or week 2 (depending on the score). • As this was an observational, non-interventional study, only distribution-based analyses were possible (not anchor-based).
Stage 5: Recall period comparison	
Comparison of 4-hour and 24-hour recall period versions of the COP-Q Symptom Module	To explore whether there are differences in the information gained from the 4-hour recall periods and the 24-hour recall period versions of the COP-Q Symptom Module, the following analyses were performed: • A paired t-test was used to compare the difference between the 4-hour recall period score (AM or PM) and the 24-hour recall period score at Day 2 (and Day 9) for day-level scores. • A paired t-test was used to evaluate the difference between 7-day mean of averaged 4-hour recall (AM or PM) period score with the 7-day mean 24-hour recall period score at Week 3 (and Week 4) for 7-day averaged scores. If significantly different then there may be utility in administering a shorter recall period version of the Symptom Module more/less regularly. • Agreement between the two recall periods (4-hour and 24-hour) was assessed at Day 2 (and Day 9) using the Paired t-tests, coefficient of determination (CoD) (squared correlation between 4-hour and 24-hour) to determine the percentage of shared variance and concordance correlation coefficient (CCC) to determine reproducibility of scores [46, 47]. Evidence of the reliability of each recall period (averaged 4-hour vs. 24-hour) was collected via comparison of ICCs for averaged weekly score calculated for the 4-hour (14 scores averaged across the week) and 24-hour recall periods (7 scores averaged across the week). Non-significant paired t-test results, largely overlapping densities and large coefficients of determination and large concordance correlation coefficients would be supportive for single daily administration (4-hour recall).

COP-Q = Chronic Ocular Pain Questionnaire; VTM = Visual Tasking Module; HRQoL = health-related quality of life; CFA = Confirmatory Factor Analysis; WLSMV = weighted least square mean and variance adjusted; MLE = Maximum Likelihood Estimator; CFI = Comparative Fit Index; TLI = Tucker Lewis Index; RMSEA = Root Mean Square Error of Approximation; SRMR = Standardizes Root Mean Square Residual; IRT = Item Response Theory; RSM = Rating Scale Rasch model; MNSQ = mean square; TRT = Test-Retest Reliability; PGI-S = Patient Global Impression of Severity; ICC = Intra-class correlation coefficients; SEM = standard error of measurement; VFQ-25 = Visual Functioning Questionnaire; EQ-5D-5 L = EuroQoL-5 Dimension-5 Level; OPAS = Ocular Pain Assessment Survey; SD = standard deviation; CoD = coefficient of determination; CCC = concordance correlation coefficient

## Results

### Demographic and clinical characteristics

Overall, 124 patients with COSP from the US participated in the observational study. Due to careful quota sampling, the study sample included representation of a range of demographic and baseline clinical characteristics, including participants who varied in their underlying diagnoses associated with COSP and in their COSP severity (Table 4). Although more than half the sample (67.7%) reported that they were diagnosed with DED, many of these participants also fulfilled other recruitment quota, and there was still considerable diversity in the participants with DED. The range of COSP presentation and severity in the sample was considered adequate to support conclusions regarding the psychometric properties of the COP-Q in the intended context of use (full demographic and clinical characteristics presented in Supplementary File 5).

**Table 4:** Demographic and clinical characteristics of the psychometric analysis population at baseline (*N* = 124)

Participant sociodemographic characteristics	COP-Q psychometric analysis population (*N* = 124)
	Statistic or n (%)
**Age at enrolment into the study (years)**	
Mean (SD)	54.5 (16.53)
Median (Min, Max)	59.0 (21, 79)
**Gender**	
Female	91 (73.4%)
Male	33 (26.6%)
**Ethnicity**	
Non-Hispanic, Non-Latino or Non-Spanish origin	113 (91.1%)
Hispanic, Latino or Spanish origin (of any race)	10 (8.1%)
Prefer not to say	1 (0.8%)
**Race**	
White	63 (50.8%)
Black or African American	34 (27.4%)
Asian	23 (18.5%)
Native Hawaiian or Other Pacific Islander	1 (0.8%)
Multi-racial	3 (2.4%)
*Diagnosis	
Ophthalmological condition (DED, post-herpetic neuralgia, Graft vs. Host disease, corneal erosions etc.)	98 (79.0%)
* Ophthalmological condition patient diagnosed with*	
DED	84 (67.7%)
No other Ophthalmological conditions experienced other than COSP	7 (5.6%)
Meibomian gland dysfunction (MGD)	2 (1.6%)
Missing	2 (1.6%)
Other (Blepharitis/Meibomitis, Pterygia and Glaucoma)	3 (2.4%)
Had corneal eye surgery (refractive surgery - such as LASIK or PRK, cataract surgery, glaucoma surgery - such as MIGS)	34 (27.4%)
*Type of corneal surgery received	
Cataract	30 (75.0%)
Refractive (e.g., LASIK, PRK)	10 (25.0%)
Non-ophthalmic condition (systemic lupus erythematosus, rheumatoid arthritis, Sjögren’s syndrome etc.)	15 (12.1%)
**Clinician-rated COSP severity at screening**	
Mild	16 (12.9%)
Moderate	75 (60.5%)
Severe	32 (25.8%)
Very Severe	1 (0.8%)

*More than 1 response option selected

SD = standard deviation; DED = Dry Eye Disease; LASIK = Laser-Assisted In Situ Keratomileusis; PRK = Photorefractive Keratectomy; MIGS = Minimally Invasive Glaucoma Surgery; MGD = Meibomian Gland Dysfunction

### Item properties

Missing data for all COP-Q modules was minimal throughout the study. Although the COP-Q was programmed to allow participants to skip items, for each of the modules, for all participants (and each module), either they completed the whole module or when at least one item was missed, the whole module was missed, providing reassurance that items were not skipped due to being confusing or considered inappropriate. Responses were distributed across the full response scale for the majority of COP-Q items at most timepoints, illustrating that the COP-Q response scales were able to capture the full range of severity of COSP symptoms or impacts. Ceiling effects were observed for a small number of items in the VTM (Item 6; ‘Drive during the day’ and 7; ‘Look in the mirror for example to shave or put make-up on’), the Symptom Module (Item 3; ‘Burning of the eye’), the HRQoL Module (Item 1; ‘Low/Depressed’) and the Sleep Module, where item responses were skewed towards the less severe end of the scale. However, it was considered of importance to retain these items to assess the full spectrum of COSP symptoms and impacts. No floor effects were observed. Full item response distribution tables and graphs for the COP-Q are provided in Supplementary File 6.

All items within the multi-item COP-Q modules demonstrated correlations of > 0.40 with the other items in their module (r range = 0.44–0.87), which provides evidence of bivariate relationships between items and suggests the items are assessing similar/related constructs. In addition, no correlations for any of the COP-Q multi-item modules were > 0.90 which would have indicated high likelihood of redundancy (See Supplementary File 7 for full results).

### Dimensionality and scoring

#### Factor analysis

Confirmatory factor analysis (CFA) was conducted to assess the hypothesized unidimensional structure for each module (Fig. 2). The multi-level CFA results provided ‘fair’ support for the unidimensional structure of the Symptom Modules (all recall periods), with all items showing adequate factor loadings (> 0.40), but some of the fit statistics did fall slightly below the recommended thresholds [37] (see Table 5 and last column for target model fit thresholds; Model 1 found in Supplementary File 8). Assessment of residual correlations indicated shared unexplained variance between Item 6 (‘Feeling like something is in your eye’) and Item 7 (‘Eye itch’) in the Symptom Module. Based on the prior qualitative research, it was hypothesized that such shared variance could be due to conceptual overlap between the two items; they may be different descriptions of the same sensation. Feedback from clinical experts in COSP (authors PH and PK) indicated the clinical importance and relevance of both items. Thus, both items were retained, and a residual error correlation term was specified to account for this shared variance (See Table 5; Model 2 found in Supplementary File 8). Specification of the residual error term improved model fit for the 4-hour (AM) and 24-hour recall periods whilst the 4-hour (PM) marginally reduced.

**Table 5:** Multi-level CFA model fit indices for the symptom module

Model fit index	Model 1 (all items)			Model 2 (Residual error term)			A priori target model fit thresholds
	4 h (AM)	4 h (PM)	24 h	4 h (AM)	4 h; (PM)	24 h	
CFI	0.907	0.933	0.891	0.933	0.924	0.948	> 0.95
TLI	0.861	0.899	0.836	0.892	0.877	0.916	> 0.95
RMSEA	0.103	0.079	0.1	0.091	0.087	0.072	< 0.08
SRMR	0.037	0.025	0.054	0.032	0.034	0.038	< 0.05

CFI = Comparative Fit Index; TLI = Tucker Lewis Index; RMSEA = Root Mean Square Error of Approximation; SRMR = Standardizes Root Mean Square Residual [34–36]

The CFA models showed moderately good fit statistics (except RMSEA) and factor loadings > 0.70 for the VTM and HRQoL Module (see Tables 6 and 7). However, CFA highlighted residual correlations of Item 7 (‘Look in the mirror for example to shave or put make-up on’) and Item 5 (‘Drive at night’) with Item 2 (‘Read on a screen for example a computer or a tablet’) of the VTM, suggesting that these items share variance not attributable to the common factor, potentially undermining the unidimensionality of the VTM.

**Table 6:** Visual tasking module factor loadings

VTM Items	Factor loading
Item 1. Read books, newspapers or magazines for more than one hour?	0.82
Item 2. Read on a screen or example a computer or tablet for more than one hour?	0.80
Item 3. Watch a program on the TV for more than one hour?	0.88
Item 4. Watch events at a distance for example a show or sporting event?	0.84
Item 5. Drive at night?	0.75
Item 6. Driving during the day?	0.85
Item 7. Look in the mirror for example to shave or put make-up on?	0.82
Item 8. Carry out your usual leisure activities or hobbies for example crafts, painting, playing cards?	0.87

**Table 7:** CFA model fit indices for the VTM and HRQoL modules

Model fit index	VTM	HRQoL Module	A priori target model fit thresholds
	All Items (8)	All items (4)	
CFI	0.984	0.999	> 0.95
TLI	0.978	0.997	> 0.95
RMSEA	0.124	0.103	< 0.08
SRMR	0.045	0.007	< 0.05

CFI = Comparative Fit Index; TLI = Tucker Lewis Index; RMSEA = Root Mean Square Error of Approximation; SRMR = Standardizes Root Mean Square Residual [34–36]

#### Rasch analysis

Infit and outfit statistics did not identify any items that deviated from the Rasch model expectations (full results in Supplementary File 9). Person fit residuals were assessed for any values outside of the range 0 ± 2.5. Nine participants were outside this range for the VTM and only two for the HRQoL Module. Such a small percentage outside of this range are unlikely to impact the utility of these modules so no items were removed. Item characteristic curves (item parameters reported in Supplementary File 10 and item characteristic curves in Supplementary File 11) did not indicate any spurious or overlapping response options and followed a logical pattern compared to theta (θ). Item-person maps were generated (see Supplementary File 12) to assess item redundancy and accurate endorsement across the latent-trait. Two sets of items overlapped on the ‘Difficulty’ scale for the VTM (Item 1 and 5; Items 6 and 8) and one pair of items for the HRQoL Module (Items 2 and 4). These items were not removed based on this analysis as it was determined that the items assess different aspects of similar concepts and were important to retain.

To assess local dependency of the VTM and HRQoL Module items, Yen’s Q3 statistic was produced to assess residual correlations between item pairs [39, 40]. No items for the HRQoL Module were above the threshold (average residual correlation + 0.30 = 0.021), however, Items 7 and 8 for the VTM exceeded this cut off of 0.159 (full results in Supplementary File 13).

Thus, Rasch analysis supported the unidimensional structure of the modules but highlighted a few potential items for removal that deviate from the unidimensional structure of the VTM. Furthermore, Rasch analysis suggested that the response options were appropriate, with participants who experienced more severe HRQoL or visual tasking impairment selecting the more extreme response categories (and vice versa). In addition, the Rasch analysis was re-run on the new item structure for the VTM (6 items) which provided similar item parameters and did not indicate any misfitting items. Therefore, the new item set indicated that sum score sufficiency held true for the new set of items and the scale was demonstrably improved (analysis not included but available on request).

#### Item reduction and finalized scoring of COP-Q

**Fig. 3:**
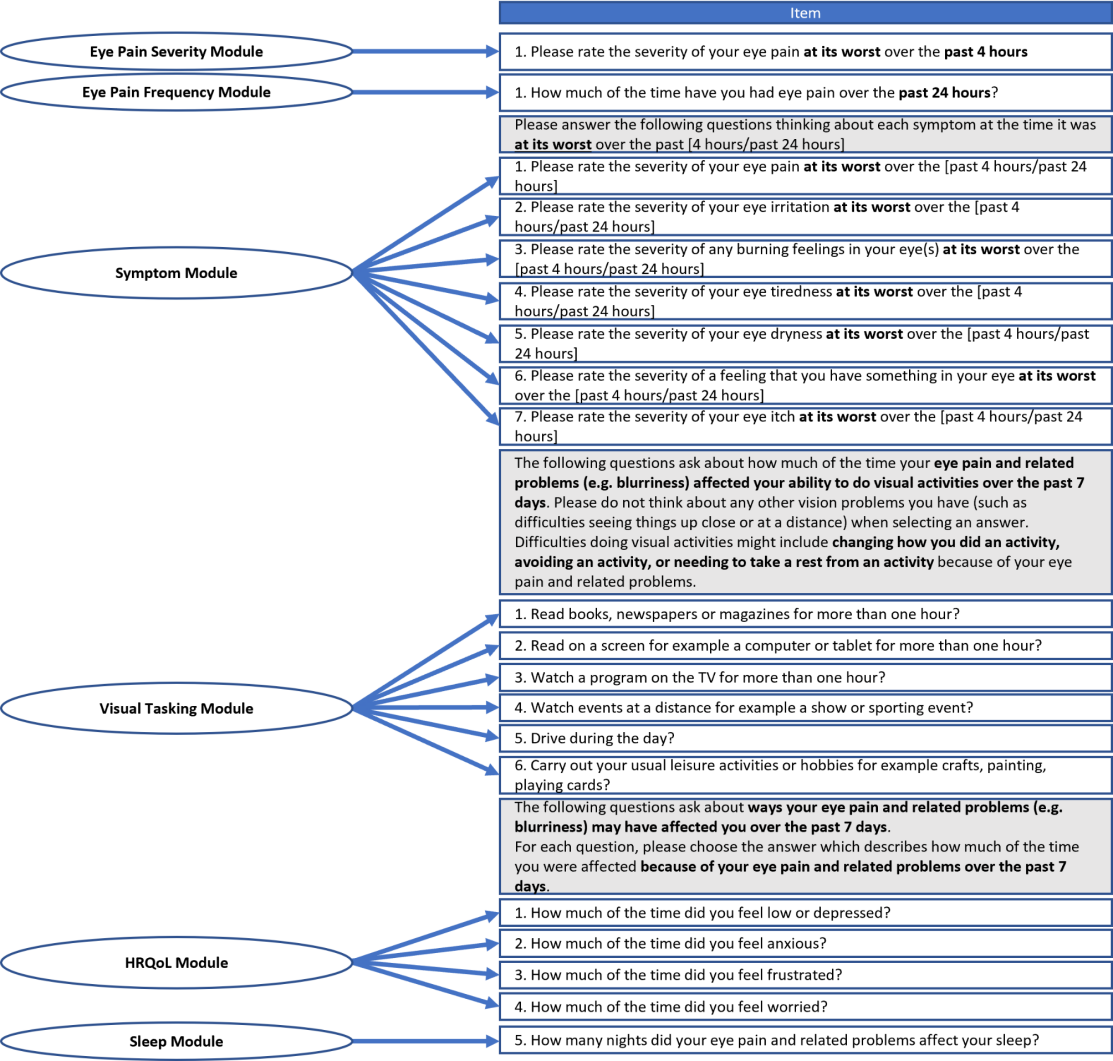
Updated conceptual framework of the COP-Q

Following consideration of results from the above item-level and dimensionality analyses, the study team discussed potential item deletion and finalization of COP-Q scoring, with input from two expert ophthalmologists in COSP (PH and PK; both authors of this paper). Ceiling effects observed in the item response distributions, residual correlations > 0.1 from the CFA, item overlap highlighted by the Rasch analysis, and input from clinical experts led to the decision to remove Item 5 (‘Drive at night’) and Item 7 (‘Looking in the mirror to shave or put make-up on’) from the VTM, resulting in a six-item VTM (Fig. 3). It was judged that all other items of the COP-Q were important to retain to assess a range of symptoms relevant to the COSP experience. Items discussed for removal are detailed in Supplementary File 14, with justification for deletion/retention.

CFA was then conducted again to assess the factor structure with these selected items removed from the VTM. With Items 5 and 7 removed, factor loadings were still > 0.40 and most fit indices were excellent, with the exception of RMSEA which was only marginally above the desired threshold (see Tables 8 and 9), indicating a good model fit for the unidimensional structure of the VTM.

**Table 8:** Post-item removal model fit indices for the VTM

Model fit index	VTM	A priori target model fit thresholds
	Post Item Removal	
CFI	0.993	> 0.95
TLI	0.988	> 0.95
RMSEA	0.105	< 0.08
SRMR	0.029	< 0.05

CFI = Comparative Fit Index; TLI = Tucker Lewis Index; RMSEA = Root Mean Square Error of Approximation; SRMR = Standardizes Root Mean Square Residual [34–36]

**Table 9:** VTM factor loadings following the removal of item 5 (‘drive at night’) and item 7 (‘look in the mirror for example to shave or put make-up on’)

VTM Items	Factor loading
Item 1. Read books, newspapers or magazines for more than one hour?	0.84
Item 2. Read on a screen or example a computer or tablet for more than one hour?	0.83
Item 3. Watch a program on the TV for more than one hour?	0.89
Item 4. Watch events at a distance for example a show or sporting event?	0.85
Item 6. Driving during the day?	0.82
Item 8. Carry out your usual leisure activities or hobbies for example crafts, painting, playing cards?	0.82

Following item deletion, a scoring algorithm for the COP-Q was established. Dimensionality analyses and empirical comparison between unconstrained and constrained factor loadings supported calculation of an unweighted sum score (i.e., the constrained model did not fit markedly worse than the unconstrained model) for each Symptom Module version (24-hour and 4-hour recall period versions), VTM, and HRQoL Module. For the Eye Pain Severity Module, Eye Pain Frequency Module and Symptom Module, a 7-day average score is recommended to define clinical trial endpoints. Such an approach to scoring has the benefit that it is intuitive, simple to implement and equally aids ease of interpretation. Such benefits are desirable for a trial endpoint but will be of even greater value if the COP-Q is used to monitor the severity of COSP symptoms and associated functional impacts in clinical practice in the future.

Additionally, to inform the maximum number of missing items acceptable to form a multi-item daily score, Cronbach’s alpha, standard error of measurement (SEM = SD$\:\surd\:[1-reliability]$) and the Spearman-Brown prophecy formula [48] were used. It was determined that no more than one item (for the HRQoL Module) or two items (for the VTM and Symptom Module) should be missing for a daily score to be calculated. For all 7-day average scores, no more than three days can be missing before reliability drops significantly. The ICC value of 7-day average scores was used with the Spearman-Brown prophecy formula, to estimate this (full results available in Supplementary File 15).

### Reliability and validity of scores

#### Reliability

All Cronbach’s alpha coefficients for the Symptom Module score (all recall periods) and the VTM and HRQoL Module scores were well above the a priori threshold of 0.70, providing evidence of very high internal consistency reliability (alpha range: 0.90–0.96). The VTM alpha coefficient prior to the removal of Item 5 and Item 7 was 0.92 and after removal was 0.90, indicating that the two items did not contribute significantly to the scale and their removal was not detrimental. All findings provide further support that the multi-item COP-Q modules scores are unidimensional and assess a singular latent trait.

Test-retest reliability (TRT) for the 7-day average scores of the daily modules was good to excellent (0.81–0.94; Table 10). For the VTM, the TRT over two weeks surpassed the threshold for good (ICC = 0.79) and was slightly superior to the ‘moderate’ results for the one-week interval (ICC = 0.72). Conversely the HRQoL Module demonstrated good TRT reliability over the one-week period (ICC = 0.86) and moderate reliability over two weeks (ICC = 0.68). The Sleep Module had only fair TRT reliability over both one- and two-week periods (ICC range: 0.65–0.67).

**Table 10:** Test-retest level of agreement/reproducibility for the COP-Q module scores

COP-Q domain score	Score type/Time points	*N*	Test-retest reliability results
			ICC coefficients (Kappa for single item Sleep Module*)
Eye Pain Severity Module (4-hour recall period AM)	7-day average	75	0.940
Eye Pain Severity Module (4-hour recall period PM)	7-day average	75	0.940
Eye Pain Frequency Module (24-hour recall period)	7-day average	73	0.814
Symptom Module (4-hour recall period version AM)	7-day average	67	0.916
Symptom Module (4-hour recall period version PM)	7-day average	67	0.915
Symptom Module (24-hour recall period version)	7-day average	61	0.917
VTM (7-day recall period)	Between Week 1 and Week 2	68	0.720
	Between Week 1 and Week 3	60	0.789
HRQoL Module (7-day recall period)	Between Week 1 and Week 2	75	0.864
	Between Week 1 and Week 3	60	0.681
Sleep Module (7-day recall)	Between Week 1 and Week 2	75	0.651
	Between Week 1 and Week 3	60	0.668

*The single-item Sleep Module score used weighted Kappa reliability coefficient instead of an ICC

ICC: Intraclass correlation; HRQoL: Health-related Quality of Life

#### Construct validity

Convergent validity was supported for all COP-Q modules with an adequate number of hypothesized relationships, in which correlations of *r* ≥ 0.50 were observed, and with a generally logical pattern of correlations, providing evidence that the measures are assessing the intended constructs of interest (full correlation results in Supplementary File 16).

All COP-Q module scores showed evidence of the ability for the module scores to distinguish between severity groups, demonstrating known-groups validity. Where groups were defined using PGI-S scores, mean scores increased monotonically across severity groups with statistically significant differences between all adjacent groups and moderate-large effect sizes between most adjacent groups (range: 0.52–2.97; see Table 11), with the exception of the PGI-S limitations in visual activities item for the Eye Pain Severity Module. For groups defined by patient- or clinician-reported COSP severity, differences between severity groups were statistically significant between most adjacent groups and the effect sizes indicated that the difference between the groups were mostly moderate-to-large in magnitude, although none of the modules were able to significantly distinguish between the ‘Mild’ and ‘Moderate’ COSP severity groups (full known-groups results provided in Supplementary File 17).

**Table 11:** Known-groups validity for COP-Q modules using PGI-S items

COP-Q Module	Item/Score Anchor	*n*	Median	Mean (SD)	Between groups effect size	Pairwise *p*-value
Eye Pain Severity Module (AM)	PGI-S Eye Pain*					
	1 – Mild (Reference group)	48	2.7	2.9 (1.87)	-	-
	2 – Moderate	45	5.8	5.6 (2.23)	1.29	< 0.001
	3 – Severe	9	8.0	7.6 (1.18)	2.62	< 0.001
	PGI-S Eye Pain and Related Problems*					
	1 - Mild (Reference group)	49	2.7	2.9 (2.09)	-	-
	2 - Moderate	46	5.3	5.1 (2.15)	1.03	< 0.001
	3 - Severe	11	8.2	7.9 (1.08)	2.56	< 0.001
	PGI-S Limitations in Visual Activities*					
	0 - None	25	2.0	2.8 (2.86)	-0.39	0.540
	1 - Mild (Reference group)	51	3.3	3.7 (2.18)	-	-
	2 - Moderate	28	5.4	5.1 (2.13)	0.65	0.062
	3 - Severe	8	8.6	8.2 (1.11)	2.16	< 0.001
Eye Pain Severity Module (PM)	PGI-S Eye Pain*					
	1 – Mild (Reference group)	48	3.3	3.4 (1.85)	-	-
	2 – Moderate	45	5.9	5.9 (1.77)	1.42	< 0.001
	3 – Severe	9	8.0	7.8 (1.08)	2.45	< 0.001
	PGI-S Eye Pain and Related Problems*					
	1 - Mild (Reference group)	49	3.0	3.2 (1.90)	-	-
	2 - Moderate	46	5.6	5.7 (1.78)	1.36	< 0.001
	3 - Severe	11	8.1	7.9 (1.10)	2.66	< 0.001
	PGI-S Limitations in Visual Activities*					
	0 - None	25	1.9	2.8 (2.61)	-0.60	0.047
	1 - Mild (Reference group)	51	4.0	4.1 (2.04)	-	-
	2 - Moderate	28	5.8	5.9 (1.65)	0.91	0.002
	3 - Severe	8	8.2	8.3 (0.63)	2.20	< 0.001
Eye Pain Frequency Module	PGI-S Eye Pain*					
	1 - Mild (Reference group)	42	1.6	1.5 (0.64)	-	-
	2 - Moderate	61	2.2	2.3 (0.65)	1.21	< 0.001
	3 - Severe	10	3.1	3.0 (0.42)	2.43	< 0.001
	PGI-S Eye Pain and Related Problems*					
	1 - Mild (Reference group)	38	1.3	1.4 (0.57)	-	-
	2 - Moderate	58	2.2	2.4 (0.64)	1.56	< 0.001
	3 - Severe	14	3.0	2.9 (0.48)	2.70	< 0.001
	PGI-S Limitations in Visual Activities*					
	1 - Mild (Reference group)	65	2.0	1.8 (0.73)	-	-
	2 - Moderate	35	2.3	2.5 (0.68)	0.92	< 0.001
	3 - Severe	5	3.0	3.0 (0.13)	1.68	0.003
Symptom Module (4-hour AM)	PGI-S Eye Pain*					
	1 - Mild (Reference group)	48	18.7	21.9 (12.81)	-	-
	2 - Moderate	45	34.4	35.5 (14.63)	0.99	< 0.001
	3 - Severe	9	52.4	48.4 (11.31)	2.10	< 0.001
	PGI-S Eye Pain and Related Problems*					
	1 - Mild (Reference group)	49	18.3	19.7 (11.66)	-	-
	Response of 2 – Moderate	46	33.9	34.5 (13.05)	1.20	< 0.001
	3 – Severe	11	57.3	53.5 (10.04)	2.97	< 0.001
	PGI-S Limitations in Visual Activities*					
	0 - None	25	17.1	17.4 (14.39)	-0.60	0.092
	1 - Mild (Reference group)	51	23.4	25.6 (13.33)	-	-
	2 - Moderate	28	35.1	35.1 (14.52)	0.69	0.024
	3 - Severe	8	58.9	55.8 (9.77)	2.33	< 0.001
Symptom Module (4-hour PM)	PGI-S Eye Pain*					
	1 - Mild (Reference group)	48	20.0	23.9 (12.44)	-	-
	2 - Moderate	45	38.7	38.1 (13.55)	1.09	< 0.001
	3 - Severe	9	52.4	49.9 (11.05)	2.13	< 0.001
	PGI-S Eye Pain and Related Problems*					
	1 - Mild (Reference group)	49	19.4	21.2 (11.05)	-	-
	2 – Moderate	46	39.1	38.1 (11.44)	1.50	< 0.001
	3 – Severe	11	58.8	53.6 (11.12)	2.93	< 0.001
	PGI-S Limitations in Visual Activities*					
	0 – None	25	15.0	17.9 (13.92)	-0.74	0.012
	1 - Mild (Reference group)	51	24.6	27.7 (12.81)	-	-
	2 - Moderate	28	40.4	39.3 (12.39)	0.92	< 0.001
	3 - Severe	8	59.2	57.4 (6.63)	2.44	< 0.001
Symptom Module (24-hour)	PGI-S Eye Pain*					
	1 – Mild (Reference group)	43	22.3	24.5 (12.47)	-	-
	2 – Moderate	54	37.4	37.2 (13.53)	0.97	< 0.001
	3 – Severe	13	48.9	49.1 (10.58)	2.04	< 0.001
	PGI-S Eye Pain and Related Problems*					
	1 – Mild (Reference group)	39	21.4	23.1 (11.06)	-	-
	2 – Moderate	54	37.9	37.9 (13.58)	1.17	< 0.001
	3 – Severe	11	49.0	51.4 (7.75)	2.70	< 0.001
	PGI-S Limitations in Visual Activities*					
	1 – Mild (Reference group)	55	29.1	28.9 (13.89)	-	-
	2 – Moderate	33	43.1	40.8 (13.16)	0.88	0.001
	3 – Severe	3	59.2	59.9 (1.31)	2.27	0.002
VTM	PGI-S Eye Pain*					
	1 - Mild (Reference group)	47	5.0	5.6 (4.24)	-	-
	2 - Moderate	61	9.0	9.3 (4.30)	0.86	0.002
	3 - Severe	10	12.5	13.5 (6.06)	1.73	< 0.001
	PGI-S Eye Pain and Related Problems*					
	1 - Mild (Reference group)	46	5.0	5.1 (3.67)	-	-
	2 - Moderate	58	9.5	9.5 (4.24)	1.11	< 0.001
	3 - Severe	14	12.0	12.7 (6.02)	1.78	< 0.001
	PGI-S Limitations in Visual Activities*					
	1 - Mild (Reference group)	78	6.0	6.1 (4.05)	-	-
	Response of 2 - Moderate	35	12.0	11.8 (4.24)	1.39	< 0.001
	3 - Severe	5	16.0	14.2 (4.71)	1.98	< 0.001
HRQoL Module	PGI-S Eye Pain*					
	1 - Mild (Reference group)	47	2.0	3.0 (3.04)	-	-
	2 - Moderate	61	5.0	4.7 (3.65)	0.52	0.093
	3 - Severe	10	9.5	10.0 (4.64)	2.10	< 0.001
	PGI-S Eye Pain and Related Problems*					
	1 - Mild (Reference group)	46	2.0	2.7 (2.79)	-	-
	2 - Moderate	58	5.0	4.9 (3.85)	0.64	0.023
	Response of 3 - Severe	14	8.0	8.3 (4.70)	1.67	< 0.001
	PGI-S Limitations in Visual Activities*					
	1 - Mild (Reference group)	78	2.0	3.1 (2.82)	-	-
	2 - Moderate	35	8.0	6.8 (4.42)	1.10	< 0.001
	3 - Severe	5	8.0	10.0 (4.00)	2.40	< 0.001
Sleep Module	PGI-S Eye Pain*					
	1 - Mild (Reference group)	47	0.0	0.6 (0.71)	-	-
	2 - Moderate	61	1.0	1.2 (1.16)	0.66	0.013
	3 - Severe	10	2.5	2.3 (1.16)	2.15	< 0.001
	PGI-S Eye Pain and Related Problems*					
	1 - Mild (Reference group)	46	0.0	0.5 (0.62)	-	-
	2 - Moderate	58	1.0	1.3 (1.15)	0.85	< 0.001
	3 - Severe	14	2.0	2.0 (1.24)	1.89	< 0.001
	PGI-S Limitations in Visual Activities*					
	1 - Mild (Reference group)	78	1.0	0.6 (0.68)	-	-
	2 - Moderate	35	2.0	1.8 (1.37)	1.22	< 0.001
	3 - Severe	5	3.0	2.4 (0.89)	2.53	0.001

Population includes all participants in the psychometric analysis population with at least one COP-Q item at any timepoint

The between-groups effect size is using Hedge’s g compared to the reference group (ref). Hedge’s g is calculated as the difference in means ((comparison group) - (reference group)) divided by the pooled standard deviation

Pair-wise p-values are from two-sample t-tests testing mean score differences between corresponding group and reference group. P-values are adjusted for multiple comparisons using Bonferroni correction

*Indicates a significant F-test value (*p* < 0.05)

SD: standard deviation; PGI-S: Patient Global Impression of Severity; COSP: Chronic Ocular Surface Pain

### Preliminary exploration of interpretation of scores

Distribution-based analyses were used to provide an initial indication of what level of score changes can be considered beyond measurement error (see Table 12). For the COP-Q modules, the distribution estimates ranged from 0.4 to 3.5 points change for the respective modules (changes of > 0.7 for the Eye Pain Severity AM and PM score, > 0.4 for the Eye Pain Frequency score, > 3.3 for the Symptom Module (4-hour AM) score, > 3.3 for the Symptom Module (4-hour PM) score, > 3.5 for the Symptom Module (24-hour) score, > 2 for the VTM score, > 2 for the HRQoL Module score and > 1 for the Sleep Module). These estimates can be used as context in the triangulation of future responder estimates generated using anchor-based approaches.

**Table 12:** Distribution -based estimates of meaningful change for COP-Q modules

COP-Q Module	*n*	½ SD	SEM
Eye Pain Severity Module AM [1] at Week 3 or 4	124	1.348	0.658
Eye Pain Severity Module PM [1] at Week 3 or 4	124	1.274	0.625
Eye Pain Frequency Module at Week 2	124	0.418	0.360
Symptom Module (with item 7 - Eye itch) at Week 3 or 4			
Symptom Module (4-hour recall AM)	124	8.567	3.262
Symptom Module (4-hour recall PM)	124	8.286	3.275
Symptom Module (24-hour recall)	124	8.097	3.464
Symptom Module (without item 7 - Eye itch) at Week 3 or 4			
Symptom Module (4-hour recall AM)	124	7.394	2.940
Symptom Module (4-hour recall PM)	124	7.116	2.978
Symptom Module (24-hour recall)	124	6.947	3.168
VTM at Week 2	118	2.506	1.540
HRQoL Module at Week 2	118	1.980	1.069
Sleep Module at Week 2	118	0.556	0.656

Abbreviations: COP-Q: Chronic Ocular Pain Questionnaire; HRQoL: Health Related Quality of Life; SEM: standard error of measurement; SD: standard deviation

The COP-Q psychometric analysis population includes participants enrolled into the study with at least one item completed on the COP-Q at any time point

The SEM is calculated as the standard deviation at Week 2 or Week 3 or Week 4 (depending on the score), multiplied by the square root of one minus the reliability of the score [SD * (1-r)1/2]. For single items, the ICC reliability (week 1 to week 3 or week 2 to week 4) will be used

[1] Eye Pain Severity Module AM and PM is made up of responses from Item 1 (Eye pain [4-hour recall Symptom Module]) and forms a single endpoint score ‘Eye Pain Severity Module’ as well as being included in the Symptom Module total score

### Recall period comparison

The different recall periods for the COP-Q Symptom Module were compared to assess if they capture similar or different information and to determine which recall period is recommended for use in clinical trials. Paired t-tests, Coefficient of Determination (CoD), and Concordance Correlation Coefficient (CCC) [46, 47] statistics results indicated that the 4-hour and 24-hour recall period versions of the Symptom Module show statistically significant differences, but the magnitude of the difference was small, and each version shares a high level of reproducibility in scores. These findings suggest that such differences could be due to real fluctuations in symptom severity throughout the day and not sub-optimal measurement properties. Irrespective, the symptom severity fluctuations are small in magnitude and are unlikely to be large enough to influence choosing one recall period over the other. Findings also indicate that the different recall period versions possess similar psychometric properties. Although the 24-hour recall period has slightly better CFA model fit and marginally better convergent validity results, the 4-hour PM recall period version has marginally better known-groups validity and a smaller mean number of missing values throughout the study period and all differences were negligible.

## Discussion

Initial development of the COP-Q as a COSP-specific PRO measure to assess symptoms and quality of life was informed by previous in-depth qualitative research with the population of interest (COSP patients) [15]. This prior research was conducted to ensure the items were relevant and worded in ways easily understood by patients and aligned with FDA guidance for the development of a fit-for-purpose PRO [15, 26–29]. The present study provides the first assessment of psychometric properties for the COP-Q using data from a longitudinal, observational study with COSP patients. Analyses were used to establish dimensionality and scoring of the instrument and provide good evidence supporting the construct validity and reliability of the COP-Q.

There were low levels of missing data throughout the data collection period, providing evidence that items were not confusing or considered inappropriate. Inter-item correlations illustrated that all items within each module converge to assess a similar unidimensional construction and no item redundancy was indicated. Further support for the underlying structure of the COP-Q was provided by acceptable CFA model fit indices and Rasch analysis. There was some deviation from acceptable fit indices for the VTM, where CFA highlighted residual correlations between Item 7 (‘Look in the mirror for example to shave or put make-up on’) and Item 5 (‘Drive at night’) with Item 2 (‘Read on a screen for example a computer or a tablet’), suggesting that these items shared variance not attributable to the common factor. When Items 5 and 7 were removed, fit indices indicated a good model fit for the unidimensional structure of the VTM. Removal of Item 5 was considered acceptable as another item covering the concept of impact on driving was retained, and similarly, other items were deemed to measure similar visual functioning to that assessed by Item 7. Thus, the decision was made to remove items 5 and 7, resulting in a six-item VTM.

Analyses provide evidence that each module of the COP-Q provides a valid and reliable assessment of the concept(s) it intends to measure. Internal consistency reliability was very high and not improved by removing any items in the scale, providing further support that each multi-item COP-Q module is unidimensional. TRT was generally good, indicating that 7-day mean scores remain consistent over time in stable patients, with the exception of the Sleep Module which had only ‘fair’ reliability. The lower TRT value for this module may be due to the fact that none of the anchors were specifically focused on sleep impact.

Convergent validity was supported for all COP-Q modules with the majority of hypothesized relationships observed. Importantly, there was a generally logical pattern of correlations with other measures or theoretically related concepts (namely the VFQ-25 and OPAS), providing evidence that the modules measure what they claim to. Hypothesized correlations with the EQ-5D-5 L were not observed, which is likely because the generic EQ-5D-5 L domains (mobility, self-care, usual activities, pain/discomfort, and anxiety/depression) do not assess visual functioning with respect to eye pain. All COP-Q modules showed evidence of known-groups validity. However, none of the modules were able to significantly distinguish between the ‘Mild’ and ‘Moderate’ COSP severity groups. This could be due to the small sample size within the ‘Mild’ severity group across the modules (n = < 16/124; 12.9%). In addition, the recall periods of the COSP severity measures did not exactly match the days/weeks being examined for the COP-Q scores, which might also account for this disconnect. Importantly, the differences between severity groups were statistically significant between most adjacent groups and effect sizes indicated the differences between the groups were mostly moderate to large. Distribution-based estimates suggested in this study can be used in conjunction with anchor-based analyses conducted in future studies to facilitate meaningful change definition and interpretation of change scores for future studies employing the COP-Q.

Two different recall period versions (4-hour administered twice-a-day and 24-hours) of the Symptom Module were validated and similar psychometric measurement properties were established; thus either recall period could be used in future clinical trials/studies to evaluate COSP symptoms and would provide consistent and equally robust measurements. While the 4-hour recall period version administered twice daily may be preferred due to likely increased accuracy of recall and greater ability to capture fluctuations, this must be balanced by considerations of respondent burden and minimizing missing data. Thus, depending partly on other aspects of study design (e.g., length of trial, timing of dosing, expected onset of effect), administering either the 4-hour recall period version only once a day (in the afternoon/evening) or use of the 24-hour recall period version may be optimal in some trials.

Study limitations should be considered when interpreting findings. The sample size for this study was at the lower end typically used for psychometric validation and while adequate, as supported by broadly similar magnitude and consistency of results across COP-Q modules, ideally a larger sample would have been preferrable. Notably, the sample size was not deemed large enough to support evaluation of Differential Item Functioning and so that analysis has not been included. As COSP is not yet an established diagnosis, this was the sample that was feasible to recruit. However, quota sampling ensured this was diverse in demographic and clinical characteristics, albeit 73.4% were female and mean age was 54.5 years old, indicating the sample response may be slightly skewed towards older females. Although prevalence is unknown for COSP (given limited research to-date), DED has a 14x higher rate among women than men and is estimated to affect 30% of the population aged over 50 years old [49], which may explain this sample composition. Another limitation related to the sample that should be acknowledged is that the sample was not also stratified by the clinic/site patients were recruited from. Confirmation of the psychometric properties in a larger sample in the future is recommended.

The proportion of participants reporting ‘eye itch’ in the study was quite high. As efforts were made to exclude patients with allergic conjunctivitis and seasonal ocular allergies from this study, these findings may suggest that the sensation of ‘eye itch’ is a prominent symptom of COSP. However, it is also possible that some of these participants could have had undiagnosed seasonal allergies which were not identified at screening. Although the study aimed to include patients with COSP, defining a COSP population is difficult considering COSP has no formal set of classification guidelines. Future work to establish consensus among leading experts regarding robust and appropriate diagnostic criteria for COSP would be valuable. For the comparison of the different recall period versions, it is acknowledged that what could be evaluated was limited by the data collected – making a comparison using data where the 4-hour recall version was completed multiple times within a 24-hour period would be more insightful, but that wasn’t possible within the study design employed here. In addition, assessment of the strength of association between the 24-hour and 4-hour (AM and PM) recall periods by way of ICC assessment would be beneficial to further assess suitability of recall periods.

Finally, while this study provides the first evidence of a range of psychometric properties for the COP-Q, as it was a non-interventional study it was not possible to evaluate ability to detect change over time or to perform anchor-based analyses to support estimation of meaningful change thresholds to aid interpretation of COP-Q scores. It is important that these gaps for the COP-Q modules are addressed using data from an interventional study in the future.

## Conclusion

Findings from this study provide evidence that the COP-Q is a valid and reliable measure of patient-reported COSP symptoms and HRQoL impacts. Psychometric validation results, including excellent internal consistency and test-retest reliability coefficients, strong convergent validity and known-groups evidence, and acceptable to good fitting CFA models provide strong support for the adequacy of the COP-Q as a ‘fit-for-purpose’ instrument to derive trial endpoints for future clinical studies and other observational research in COSP populations. The COP-Q may also be of value for tracking COSP severity in general clinical practice.

## Electronic supplementary material

Below is the link to the electronic supplementary material.

Supplementary Material 1

Supplementary Material 2

Supplementary Material 3

Supplementary Material 4

Supplementary Material 5

Supplementary Material 6

Supplementary Material 7

Supplementary Material 8

Supplementary Material 9

Supplementary Material 10

Supplementary Material 11

Supplementary Material 12

Supplementary Material 13

Supplementary Material 14

Supplementary Material 15

Supplementary Material 16

Supplementary Material 17

Supplementary Material 18

## Data Availability

The dataset generated and/or analyzed during the current study are available from the corresponding author on reasonable request.
