# Supplementary material for: Psychometric validation of the Chronic Ocular Pain Questionnaire (COP-Q)
Source: J Patient Rep Outcomes. 2025 Mar 12;9:32. doi: 10.1186/s41687-025-00862-9 (PMC11903982; doi:10.1186/s41687-025-00862-9)
Supplement: Supplementary file 1 — Supplementary Material 1 [file 41687_2025_862_MOESM1_ESM.docx]

| 1^st^ August 2023 |
| --- |
| NO8794D \| Version 3_0 |
| **Qualitative Research to Assess the Usability of an Electronic Version of the Chronic Ocular Pain Questionnaire (COP-Q)** |
| Report prepared for |
|  |
|  |

|  |
| --- |
|  |
|  |

Version control

| Version Number | Changes from previous version with reasons | Date delivered |
| --- | --- | --- |
| v1_0 | Original version | 6^th^ July 2023 |
| v2_0 | Novartis’ comments addressed after first round of review | 26^th^ July 2023 |
| v3_0 | Final version | 1^st^ August 2023 |

Table of contents

[Administrative structure 5](#_Toc141272559)

[List of tables 6](#_Toc141272560)

[List of figures 7](#_Toc141272561)

[List of appendices 8](#_Toc141272562)

[Abbreviations 9](#_Toc141272563)

[Executive summary 11](#_Toc141272564)

[1.1 Introduction and objectives 11](#_Toc141272565)

[1.2 Methods 11](#_Toc141272566)

[1.3 Results 12](#_Toc141272567)

[1.4 Conclusions 13](#_Toc141272568)

[2 Introduction 14](#_Toc141272569)

[3 Objectives 16](#_Toc141272570)

[3.1 Primary objectives 16](#_Toc141272571)

[4 Methodology 17](#_Toc141272572)

[4.1 Study design 17](#_Toc141272573)

[4.1.1 The COP-Q 17](#_Toc141272574)

[4.2 Study sample 18](#_Toc141272575)

[4.2.1 Inclusion criteria 18](#_Toc141272576)

[4.2.2 Exclusion criteria 18](#_Toc141272577)

[4.2.3 Patient quotas 19](#_Toc141272578)

[4.3 Recruitment 20](#_Toc141272579)

[4.4 Data collection 21](#_Toc141272580)

[4.5 Interview conduct 23](#_Toc141272581)

[**4.6** **Ethical conduct** 23](#_Toc141272582)

[4.6.1 Subject data protection 23](#_Toc141272583)

[4.6.2 Study withdrawal 24](#_Toc141272584)

[4.6.3 Adverse Event (AE) Reporting 24](#_Toc141272585)

[4.7 Analysis 25](#_Toc141272586)

[4.7.1 Participant identification codes 25](#_Toc141272587)

[4.7.2 Analysis of socio-demographic and clinical characteristics 25](#_Toc141272588)

[4.7.3 Analysis of the usability interview transcripts 25](#_Toc141272589)

[5 Results 27](#_Toc141272590)

[5.1 Participant demographic and clinical characteristics 27](#_Toc141272591)

[5.2 General usability of the tablet device 28](#_Toc141272592)

[5.2.1 Overall experience of tablet device 28](#_Toc141272593)

[5.3 Timing for completion of the ePRO 30](#_Toc141272594)

[5.3.1 Completion of the ePRO 31](#_Toc141272595)

[5.3.2 Time taken to complete the ePRO 31](#_Toc141272596)

[5.3.3 Preference on recall period of the Symptom Module 31](#_Toc141272597)

[5.3.4 Need to take breaks 32](#_Toc141272598)

[5.3.5 Time windows 32](#_Toc141272599)

[5.3.6 Missed questionnaires 33](#_Toc141272600)

[5.3.7 Alarms/reminders 34](#_Toc141272601)

[5.4 Visual aspects of the ePRO 35](#_Toc141272602)

[5.5 Technical aspects of the ePRO 35](#_Toc141272603)

[5.5.1 Technical difficulties 35](#_Toc141272604)

[5.5.2 Charging tablet device 36](#_Toc141272605)

[5.5.3 Touch screen 36](#_Toc141272606)

[5.5.4 Tablet device training session 36](#_Toc141272607)

[5.6 Accessing the ePRO 37](#_Toc141272608)

[5.7 Debriefing of the COP-Q items and ‘patient facing text’ 38](#_Toc141272609)

[5.7.1 COP-Q title page 38](#_Toc141272610)

[5.7.2 COP-Q Eye Pain Severity Module 39](#_Toc141272611)

[5.7.3 COP-Q Eye Pain Frequency Module 42](#_Toc141272612)

[5.7.4 COP-Q Symptom Module (4-hour recall period) 45](#_Toc141272613)

[5.7.5 COP-Q Symptom Module (24-hour recall period) 52](#_Toc141272614)

[5.7.6 COP-Q Visual Tasking Module (VTM) 52](#_Toc141272615)

[5.7.7 COP-Q Health-Related Quality of Life Module (HRQoL) 63](#_Toc141272616)

[5.7.8 COP-Q ‘Patient facing text’ 72](#_Toc141272617)

[5.7.9 Relevance of the COP-Q 74](#_Toc141272618)

[5.8 Debriefing of the PGI items 75](#_Toc141272619)

[5.8.1 PGI-S Items 75](#_Toc141272620)

[5.8.2 PGI-C Items 78](#_Toc141272621)

[5.9 Recommended changes 83](#_Toc141272622)

[6 Discussion and conclusion 99](#_Toc141272623)

[6.1 Usability interviews findings 99](#_Toc141272624)

[6.1.1 General usability of the ePRO 99](#_Toc141272625)

[6.1.2 Debriefing of the COP-Q and PGI items 100](#_Toc141272626)

[6.1.3 AV PCO recommendations 101](#_Toc141272627)

[6.2 Limitations 102](#_Toc141272628)

[6.3 Conclusions 102](#_Toc141272629)

[7 References 103](#_Toc141272630)

[Appendix A. ePRO screenshots 105](#_Toc141272631)

[Appendix B- COP-Q 140](#_Toc141272632)

[Appendix C. PGI items 148](#_Toc141272633)

List of tables

[Table 1. Sample quotas for the usability interviews participants (N=10). 19](#_Toc141271835)

[Table 2. Demographic and clinical characteristics (N=10). 27](#_Toc141271836)

[Table 3. Tablet device aspects that participants liked (N=10). 29](#_Toc141271837)

[Table 4. Tablet device aspects that participants disliked (N=10). 29](#_Toc141271838)

[Table 5. Recall period preference (N=10). 31](#_Toc141271839)

[Table 6. Missed diary entries (N=10). 33](#_Toc141271840)

[Table 7. Visual aspects of the ePRO (N=10). 35](#_Toc141271841)

[Table 8. COP-Q title page key findings (N=10). 38](#_Toc141271842)

[Table 9. Eye Pain Severity Module key findings (N=10). 40](#_Toc141271843)

[Table 10. Eye Pain Frequency Module key findings (N=10). 43](#_Toc141271844)

[Table 11. COP-Q Symptom Module (4-hour recall period) key findings (N=10)4-hour recall period). 46](#_Toc141271845)

[Table 12. VTM instructions key findings (N=10). 54](#_Toc141271846)

[Table 13. VTM items key findings (N=10). 56](#_Toc141271847)

[Table 14. HRQoL instruction key findings (N=10). 64](#_Toc141271848)

[Table 15. HRQoL items key findings (N=10). 66](#_Toc141271849)

[Table 16. ‘Patient facing text’ of the pop-up boxes key findings (N=10). 72](#_Toc141271850)

[Table 17. PGI-S items key findings (N=10). 75](#_Toc141271851)

[Table 18. PGI-C items key findings (N=10). 79](#_Toc141271852)

[Table 19. Recommended changes (N=10). 84](#_Toc141271853)

List of figures

[Figure 1. Stages of the development of the COP-Q. 17](#_Toc141271854)

[Figure 2. Overview of recruitment process 21](#_Toc141271855)

[Figure 3. Randomisation process during the data collection period. 22](#_Toc141271856)

[Figure 4. Example of participant ID code. 25](#_Toc141271857)

List of appendices

[Appendix A. ePRO screenshots 105](#_Toc141271858)

[Appendix B- COP-Q 140](#_Toc141271859)

[Appendix C. PGI items 148](#_Toc141271860)

Abbreviations

| AE | Adverse Event |
| --- | --- |
| AV PCO | Adelphi Values Patient-Centered Outcomes |
| CD | Cognitive Debriefing |
| CE | Concept Elicitation |
| COP-Q | Chronic Ocular Pain Questionnaire |
| COSP | Chronic Ocular Surface Pain |
| CRF | Case Report Form |
| CRO | Clinical Research Organization |
| DBS | Deep Brain Stimulation |
| DED | Dry Eye Disease |
| EphMRA | European Pharmaceutical Market Research Association |
| ePRO | Electronic Patient-Reported Outcome |
| FDA | US Food and Drug Administration |
| GvHD | Graft versus Host Disease |
| HCP | Health Care Professional |
| HRQoL | Health-Related Quality of Life |
| ICF | Informed Consent Form |
| ID | Identification |
| IRB | Independent Review Board |
| LASIK | Laser-Assisted in Situ Keratomileusis |
| MGD | Meibomian Gland Dysfunction |
| NRS | Numerical Rating Scale |
| PCIOL | Posterior Chamber Intraocular Lens |
| PGI-C | Patient Global Impression of Change |
| PGI-S | Patient Global Impression of Severity |
| PRK | Photorefractive keratectomy |
| PRO | Patient-Reported Outcome |
| rTMS | Repetitive Transcranial Magnetic Stimulation |
| TV | Television |
| US | United States |
| VTM | Visual Tasking Module |
| WPAI+CIQ | Workplace Productivity and Activity Impairment plus Classroom Impairment Questionnaire |

Executive summary

## Introduction and objectives

Ocular pain is considered a secondary symptom in various ophthalmic conditions and can also be associated with the consequences of recent eye surgery, recent eye trauma, or postulated mechanisms such as inflammation and sensory neuronal dysregulation[^1^](#_ENREF_1)^,^[^2^](#_ENREF_2). Chronic Ocular Surface Pain (COSP) is defined as persistent pain at the ocular surface lasting for more than three months[^1^](#_ENREF_1). Given a lack of existing patient-reported outcome (PRO) measures that are considered adequate for use in COSP clinical trials to support trial endpoints, Adelphi Values Patient Centred-Outcomes (AV PCO), in collaboration with Novartis, have developed the Chronic Ocular Pain Questionnaire (COP-Q) to measure the symptoms and impacts experienced by patients with COSP. The COP-Q includes five modules (Eye Pain Severity Module, Eye Pain Frequency Module, Symptom Module, Visual Tasking Module [VTM] and Health-related Quality of Life Module [HRQoL]) to assess eye pain and eye pain-related symptoms (such as discomfort and burning), difficulties with visual tasks (such as reading and driving) and wider Health-Related Quality of Life (HRQoL) impacts (such as mood and sleep). In addition to the COP-Q, patient global impression of change (PGI-C) and severity (PGI-S) items were developed in line with Food and Drug Administration (FDA) guidelines[^3-7^](#_ENREF_3) to be used to support psychometric evaluation analyses and estimation of meaningful change thresholds.

AV and Novartis have previously collaborated to conduct qualitative research to explore the patient experience and impact of COSP on patients’ HRQoL to assess the content validity of the COP-Q. Findings from this research informed minor modifications to the COP-Q to improve interpretation and relevance to COSP patients, which was further developed into an electronic version (ePRO).

To establish psychometric validation of the electronic version of the COP-Q, a longitudinal, observational study was conducted. Using a sub-sample of the observational study population (n=10), usability testing of ePRO and PGI items was conducted prior to the observational study to ensure equivalency to paper versions and to confirm that the electronic versions are suitable for use in the observational study, and in future clinical trials.

## Methods

The first ten participants enrolled into the wider observational study were recruited to participate in the usability interviews. The participants took part in a ‘pilot’ four-week data collection period in which they were provided with a tablet device to complete the ePRO daily, followed by a telephone usability interview. Upon receiving ethical approval, third-party vendors were contracted to recruit participants for the study. Eligible participants completed an Informed Consent Form (ICF) before enrolment into the study.

Participants completed two versions of the COP-Q Symptom Module; the 24-hour recall period version (to be completed once a day) and the 4-hour recall period version (to be completed twice a day, once in the morning and once in the evening), in consecutive weeks, in a randomized order. This enabled the comparison of each recall version in terms of measurement properties and score comparability.

After the completion of the four-week data collection period, participants took part in a 45-minute interview to assess the usability of the ePRO and the tablet device. The usability interviews consisted of two parts. In the first part of the interview, participants were asked about their overall experience of using the tablet device, the timing which the ePRO had to be completed in, and the visual and technical aspects of the ePRO. The second part of the interview involved debriefing participants on the COP-Q modules and the PGI items and exploring the relevance of these questions and response options to their experience of COSP. Participants were also asked about the ePRO’s ‘patient facing text’ included in pop-up boxes that explained how to quit, skip, or look for help during the completion of the ePRO and the accessibility of the ePRO. Participants were also asked if they had any recommendations for changes to the ePRO.

## Results

Overall, all participants reported that they found the ePRO and tablet device easy to use. Participants reported that it was easy to fit completion of the ePRO into their daily schedule, and the morning and evening response time windows were reported to be long enough and easy to comply with. However, the sample was divided on their preference for completing the COP-Q twice daily (4-hour recall period) or once daily (24-hour recall period). Participants liked having daily alarms/reminders to complete the ePRO.

Participants reported some aspects of ePRO that they disliked, including technical difficulties with the alarms/reminders not going off, the login pin not working on occasion and the longer time to complete the ePRO on days when the VTM, HRQoL Module and PGI items had to be completed (completed once a week in addition to daily or twice daily modules).

Overall, participants reported that the COP-Q items were easy to understand, and that it was easy to read the text and select a relevant response option. Participants also reported that the ‘patient facing text’ included within the quit, skip and look for help buttons were clear to understand and the quit button was easy to find. However, some participants reported finding it difficult to locate the skip and look for help buttons.

Seven participants suggested recommendations to improve the ePRO based on personal preferences, in terms of its visual aspects (for example, different font colours for item text and response options), the items and modules of the COP-Q (for example, using percentages as responses instead of a numerical or verbal rating scales) and the ‘patient facing text’ (for example, making the help and skip buttons bolder and larger so they are easy to locate). Notably, these recommendations were given as suggested improvements and the current format was not considered to be difficult to complete.

AV identified some potential areas for improvement for the benefit of future clinical trial participants. These included increasing the font size of the ‘patient facing text’ in the pop-up boxes for future studies and providing a more comprehensive training session upon participant enrolment to the study to resolve some of the technical issues reported by participants, such as changing the time and volume of the alarms and the location of the help, skip and quit buttons. Based on discussions with the third-party vendor responsible for developing the ePRO, the decision was made not to update the font size of the ‘patient facing text’ as this would have considerable implications on the study timelines and this update was recommended by participants thinking of the potential needs of future participants, rather than because they experienced issues with reading the text during the data collection period. Upon investigation, the third-party vendor confirmed that they could not replicate the technical issues faced by the participants during the usability interviews, and so it was decided that no further updates were required before moving into the next phase of the study. The development of two paper patient user guides, the Questionnaire Completion Patient User Guide and the Kayentis Web Platform Use Patient User Guide, which will provide guidance to participants in terms of completing the ePRO, navigating the tablet and setting up the alarms/reminders and internet connection, was highlighted as a solution to minimize compliance issues and to improve the overall experience of completing the ePRO.

## Conclusions

Findings from this study support the usability of the ePRO and PGI items and their suitability for use in future clinical trials. This study was the first part of a larger observational study specifically aiming to explore the usability of the ePRO. Further conclusions will be drawn from the next phase of the study aiming to evaluate the psychometric properties of the ePRO and PGI items.

# Introduction

Ocular pain is considered a secondary symptom in various ophthalmic conditions and can also be associated with the consequences of recent eye surgery, recent eye trauma, or postulated mechanisms such as inflammation and sensory neuronal dysregulation.[^1^](#_ENREF_1)^,^[^2^](#_ENREF_2) Chronic Ocular Surface Pain (COSP) is defined as persistent pain at the ocular surface lasting for more than three months. While ocular pain resulting from events such as those aforementioned is commonly reported, COSP manifests as either persistent pain-related symptoms that are out of proportion to the expected clinical signs[^1^](#_ENREF_1), or ocular pain that is experienced for a longer period of time than clinically anticipated, even when other signs of trauma/surgery have healed.[^1^](#_ENREF_1)^,^[^2^](#_ENREF_2) Due to limited research related to COSP, the prevalence of the condition is unknown. However, Dry Eye Disease (DED, which is a common underlying ophthalmic condition in COSP patients) is estimated to affect 30% of the population aged over 50 years old, with many of these patients continuing to experience ongoing ocular surface pain despite receiving treatment for DED.[^2^](#_ENREF_2)^,^[^8^](#_ENREF_8) Similarly, about 60% of patients who have had laser-assisted in situ keratomileusis (LASIK) surgery experience ocular pain lasting more than one-month post-surgery, and as many as 44% have pain lasting more than six months after surgery. Additionally, in ocular Graft versus Host Disease (GvHD), approximately half of patients develop severe ocular pain approximately six months after transplantation, which is difficult to manage despite treatments for their underlying condition.[^9-13^](#_ENREF_9)

As there are currently no diagnostic criteria for COSP, it is challenging to understand the prevalence of the condition and those affected by it. Treatment options are suggested from across the fields of neuroscience, neurology, and pain management. These options include local therapies, such as artificial tears, topical steroids, and topical immunomodulators; systemic treatments; stimulation therapies such as deep brain stimulation (DBS); repetitive transcranial magnetic stimulation (rTMS) and acupuncture. Psychological approaches include the use of behavioral therapies to help with pain management.[^1^](#_ENREF_1) At present, there is little research exploring ocular surface pain as a distinct condition in patients who experience neuropathic or nociceptive ocular surface pain. Further, it is relatively unknown how the available treatment options described above help alleviate the symptoms associated with COSP.

As part of a clinical development program in COSP, Novartis wish to capture changes in patient-reported health concepts which are relevant to the condition as part of their measurement strategy for future clinical trials. Due to a lack of published information regarding the patient experience of COSP, Adelphi Values Patient-Centered Outcomes (AV PCO) have collaborated with Novartis to conduct qualitative research exploring the patient experience and impact of COSP on patients’ Health-Related Quality of Life (HRQoL) and clinician perspectives of COSP. This qualitative research supported the development and evaluation of the content validity of new, fit-for-purpose, COSP-specific patient-reported outcome (PRO) measure. PRO measures are any measurements that are based on a report that comes directly from the patient (i.e., study subject) about the status of their health condition, without amendment or interpretation of the patient’s report by a healthcare professional (HCP) or anyone else. PRO measures can assess several aspects of a patients’ disease experience, including symptoms, impacts, functioning, and treatment satisfaction and adherence. The US Food and Drug Administration (FDA) PRO guidance outlines best practice for developing PROs intended for use to derive endpoints in clinical trials to support labelling claims.[^3-7^](#_ENREF_3)

A targeted literature and blog review was first conducted to identify important concepts related to the patient experience of COSP, to inform the development of a new PRO called the Chronic Ocular Surface Pain Questionnaire (COP-Q). Interviews were also conducted with three clinical experts with experience of treating patients with COSP (n=3), and two additional clinical expert advisors (n=2) were engaged to provide clinical input at key timepoints during the study to obtain feedback on the appropriateness of the COP-Q to support endpoints in COSP studies. Feedback was also sought from the FDA to support development of the PRO measurement strategy in COSP.

The COP-Q includes modules to assess eye pain and eye pain-related symptoms (such as discomfort and burning), difficulties with visual tasks (using the Visual Tasking Module [VTM] which was previously developed by Novartis based on research in DED), and wider HRQoL impacts (such as mood and sleep). In addition to the COP-Q, patient global impression of change (PGI-C) and severity (PGI-S) items were developed in line with FDA guidelines to be used to support psychometric evaluation analyses and estimation of meaningful change thresholds, and the Workplace Productivity and Activity Impairment plus Classroom Impairment Questionnaire (WPAI+CIQ)[^14^](#_ENREF_14) was identified as a well-validated PRO measure of work/school impacts.

To be accepted by the FDA, the content validity of PRO instruments must be assessed in the targeted clinical trial population (Context of Use) via the conduct of qualitative research with patients with the condition of interest. Therefore, as part of the previously conducted qualitative research, 24 COSP patients took part in combined concept elicitation (CE) and cognitive debriefing (CD) qualitative interviews to provide insight into the patient experience of living with COSP, and to assess the content validity of the COP-Q in this population. In addition, 15 of those patients also completed a daily diary app task across a seven-day period. This app task was designed to collect qualitative information about the symptoms and associated impacts of COSP using video, text, audio and photographic responses while patients went about their normal daily life. Findings from the patient interviews and daily diary app task informed a few modifications made to the COP-Q.

Novartis are now seeking to evaluate and document the psychometric properties of an electronic version (ePRO) of the COP-Q. Notably, the psychometric properties of the WPAI+CIQ will not be assessed given the WPAI+CIQ is already validated and well established (and is not likely to support a primary or secondary endpoint). The PGI items are intended for use as anchors only. Psychometric validation of the COP-Q will be performed via conduct of a longitudinal, observational study in COSP patients based in the US (N=124). Using a sub-sample of the observational study population (n=10), usability testing of ePRO and PGI items was conducted to ensure equivalency to paper versions and to confirm that the electronic versions are suitable for use in the observational study, and in future clinical trials. As the WPAI+CIQ has already been well validated and given the limited time during the usability interviews, this measure was not debriefed during the interviews.

This report will present the findings from the usability interviews that were performed with the first ten patients enrolled in the observational study in COSP.

# Objectives

## Primary objectives

The primary objective of this phase of the study was to:

- Conduct usability testing via telephone interviews with patients with COSP to assess usability and support refinement of the ePRO and PGI items.

# Methodology

## Study design

This was a non-interventional, qualitative semi-structured interview study conducted with COSP patients to assess the usability of the ePRO and PGI items. This was part of a larger observational study conducted to evaluate the psychometric properties of the COP-Q and to finalize scoring.

Figure 1 below summarizes the previous qualitative work and the usability and psychometric phases of the project. The blue arrow indicates the stage of the study presented within this report.

**
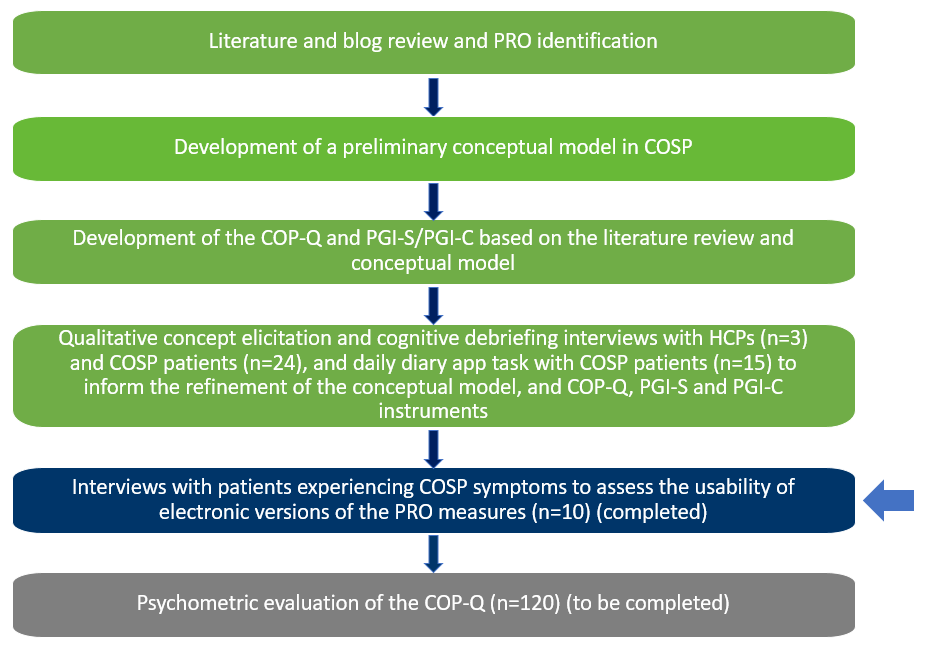
**

**Figure 1. Stages of the development of the COP-Q.**

### The COP-Q

The COP-Q consists of of five modules which assess eye pain and related symptoms, ability to carry out visual activities and HRQoL in COSP patients. These include two single-item modules, the Eye Pain Severity Module and Eye Pain Frequency Module. The seven-item Symptom Module assesses symptoms associated with COSP including ‘eye pain’, ‘eye irritation’, ‘burning of the eye’, ‘eye tiredness’, ‘eye dryness’, ‘feeling like there is something in your eye’ and ‘eye itch’. Two different recall period versions of the COP-Q Symptom Module have been developed (a 24-hour recall period version and a 4-hour recall period version), with the aim to compare these versions in terms of measurement properties and score comparability to help determine which recall period version Novartis should take forward into future clinical trials. The VTM consists of eight items which assess visual functioning in COSP patients over the ‘past seven days’. The HRQoL Module consists of five items which assess quality of life in COSP patients over the ‘past 7 days’. The COP-Q was completed by the participants as an ePRO via a tablet device.

## Study sample

A total of ten patients with COSP were targeted for usability interviews. These were recruited from the first patients enrolled into the wider observational study which aimed to recruit 120 patients in total. All study participants met the eligibility criteria outlined below. Eligibility for each participant was confirmed by a recruiting clinician at the study site. The first ten COSP patients enrolled took part in a ‘pilot’ four-week data collection period in which they were provided with a device to complete the ePRO, followed by a telephone usability interview.

It was expected that a sample size of ten patients would be sufficient to gain enough data to assess usability of the ePRO and PGI items based on published guidance.[^15^](#_ENREF_15)^,^[^16^](#_ENREF_16) Patients were required to meet the following inclusion and exclusion criteria to be eligible for inclusion in the study:

### Inclusion criteria

- Patient is an adult aged 18 or above.
- Patient has symptoms of COSP (chronic, persistent eye pain [can also be described as other symptoms e.g., burning, irritation, dryness etc.] at the ocular surface lasting for more than three months at screening, irrespective of treatments).
- The primary complaint is ocular pain coming from the surface of the eye [corneal or conjunctiva rather than systemic pain].
- On average, the patient experiences ocular pain at least four days per week in a typical week.
- Fluent speaker, literate and able to read and write in the English language.
- Willing and able to provide written or electronic informed consent and to perform all study activities.

### Exclusion criteria

- Patient has an active ocular infection.
- Patient is participating in another observational study or clinical trial.
- Patient experiences acute seasonal ocular allergies during the time they would be participating in the trial.
- Patient has any other physical or mental illness that might influence the responses they give to questions about their ocular pain or might impact the patient’s ability to participate in the study.

### Patient quotas

In addition to the above inclusion and exclusion criteria, the following patient quotas were implemented and monitored throughout recruitment to ensure that a diverse sample with representation of important demographic and clinical subgroups were recruited to the study. The aim was for the sample to include patients of both sexes with a range of COSP severity levels, ages and educational abilities, and care was taken to ensure ethnic and racial diversity within the study sample.

The sample quotas for the usability interviews are summarized below, with target figures displayed in Table 1.

- **Sex:** At least three participants of each sex to be enrolled so that there would be adequate representation of both males and females (as assigned at birth).
- **Age:** At least one participant aged 18-35 years of age, at least two participants aged 36-60 years of age, and at least two participants aged 61 years or above. Efforts were made to ensure that there were a range of severity levels within each age group.
- **Race:** At least two Caucasian/White and two non-Caucasian/White participants.
- **Educational attainment:** At least three participants who completed high school or less, and at least three participants who had some higher education beyond high school.
- **COSP severity:** At least three participants with mild COSP (those who score 1-3 on a 0-10 single item rating scale of pain intensity in the past week), at least three participants with moderate COSP (those who score 4-6 on a 0-10 single item rating of pain intensity in the past week), and at least one participants with severe COSP (those who score 7 or more on a 0-10 single item rating scale of pain intensity in the past week) as rated by the participant during the screening process. Although COSP is generally defined as patients who experience moderate to severe ocular pain, it was deemed important to include some mild patients to ensure the COP-Q is sensitive enough to capture milder symptoms.
- **Other diagnoses/symptoms**: At least three participants who have been diagnosed with an underlying ophthalmological condition that has caused COSP symptoms (e.g., DED, Sjögren's syndrome), at least three participants who experienced COSP symptoms following refractive eye surgery (e.g., PRK or LASIK), and at least three participants with a non-ophthalmological underlying condition that has caused COSP symptoms (e.g., diabetes, rheumatoid arthritis, systemic lupus erythematosus). Recruiting HCPs/clinicians at each site confirmed the pain type based on clinical notes and provision of clinical characteristics via completion of a case report form (CRF).

| **Table 1. Sample quotas for the usability interviews participants (N=10).** | | |  |
| --- | --- | --- | --- |
| **Patient characteristics** | | **Usability interviews sample quota (n=10)** |  |
| Sex | Female | ≥3 | |
|  | Male | ≥3 | |
| Age | 18-35 years old | ≥1 | |
|  | 36-60 years old | ≥2 | |
|  | >60 years old | ≥2 | |
| Race | Non-Caucasian | ≥2 | |
|  | Caucasian | ≥2 | |
| Level of education | High school or less | ≥3 | |
|  | More than high school | ≥3 | |
| COSP severity level | Severe (pain score of 7-10 on a 0-10 scale, past week recall) | ≥3 | |
|  | Moderate (pain score of 4-6 on a 0-10 scale, past week recall) | ≥3 | |
|  | Mild (pain score of 1-3 on a 0-10 scale, past week recall) | ≥1 | |
| Diagnosis | Diagnosed with an ophthalmological condition (e.g., DED, MGD, blepharitis etc.) and no history of refractive surgery or systemic comorbidities | ≥3 | |
|  | Has had refractive eye surgery (e.g., LASIK, PRK, etc.) | ≥3 | |
|  | Patients with non-ophthalmic underlying conditions (e.g., Sjögren's syndrome, diabetes, rheumatoid arthritis etc.) | ≥3 | |

## Recruitment

Participants were identified and recruited by a partner Clinical Research Organization (CRO), ORA Clinical, through four clinical sites in the US. The AV PCO team conducted site training with each of the sites, detailing the roles, responsibilities and processes involved in participant recruitment and data collection. The clinical sites were responsible for recruiting participants, confirming participant eligibility, scheduling site visits and providing training on how participants were to use the tablet device and complete the data collection period. The CRO was responsible for monitoring recruitment quotas via a tracker completed by each clinical site detailing participants’ key clinical and demographic information, as well as monitoring missed completion of any of the ePRO modules (referred to ‘diary entries’ from this point onwards). All data collected was shared with AV PCO via BOX (a secure file-sharing platform).

Patients were recruited via HCPs/clinicians at each clinical site who were asked to complete a screener form for each participant to determine eligibility for participation in the study. HCPs/clinicians then collected clinical and medical information from participants that met the eligibility criteria and patients would complete a demographic form. Based on recruitment quotas, eligible patients were recruited to the study and completed the informed consent form (ICF). An overview of the recruitment process is provided in Figure 2.


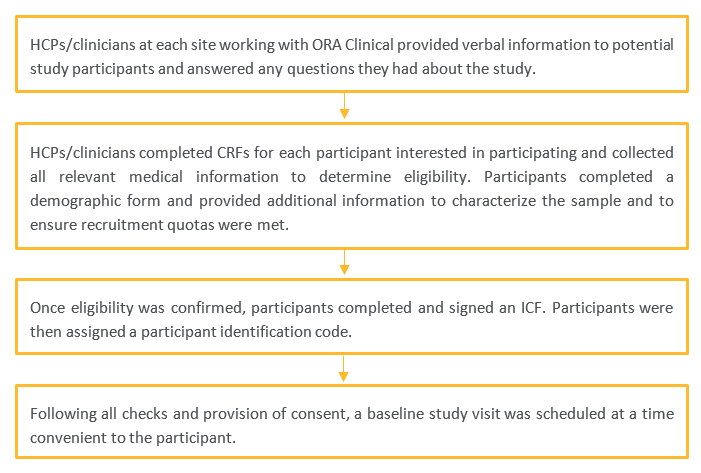


Figure 2. Overview of recruitment process

## Data collection

The first ten eligible participants were recruited to participate in a usability interview after completing the four-week data collection period. Participants completed two versions of the Symptom Module: a 24-hour recall period version and a 4-hour recall period version (Appendix B). Participants completed the 24-hour and 4-hour recall versions of the COP-Q Symptom Module in consecutive weeks, with the order randomized (see Figure 3). This enabled the comparison of each recall version in terms of measurement properties and score comparability, and ensured sufficient data was generated that both versions can be validated independently during the psychometric validation phase of the study. Participants were provided with a touch-screen tablet device during their first visit to the clinical site and were trained on how to log into the device and how to complete the questionnaire, prior to taking it home. Participants completed the COP-Q, PGI items and WPAI+CIQ at home during the four-week data collection period by tapping on the touch screen to select their responses to the items.


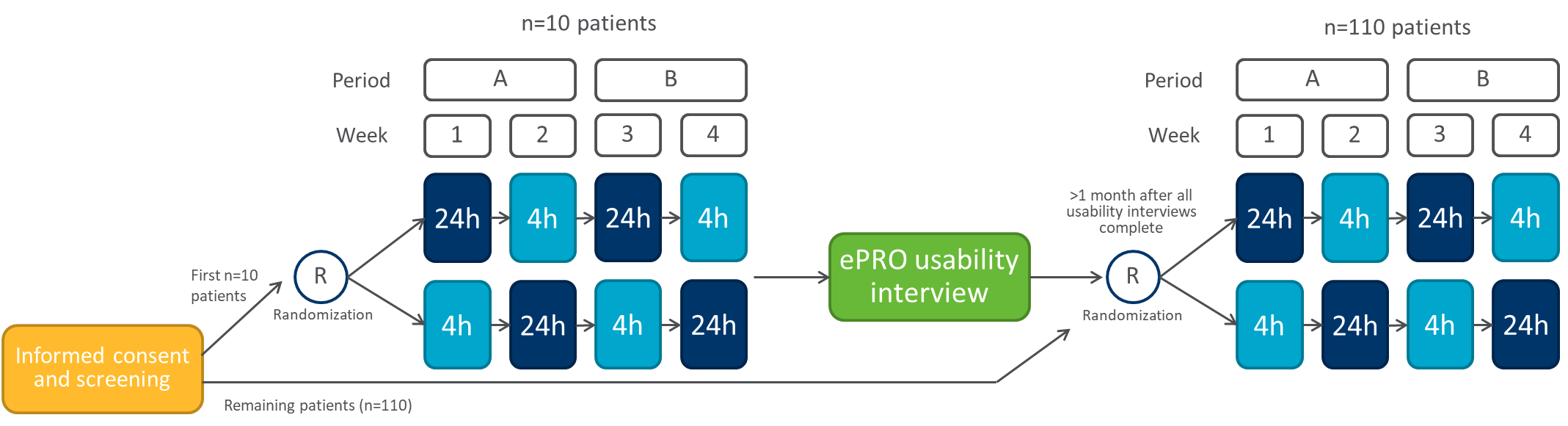


**Figure 3. Randomisation process during the data collection period.**

## Interview conduct

The usability interviews for the ePRO were conducted by telephone (using Microsoft Teams) at a time convenient for the patient and the interviewer, within seven days of them completing their four-week data collection period and were 45 minutes in duration. Trained members of the AV PCO team conducted the interviews. The purpose of the interview was to assess participants’ ability to complete the ePRO using the tablet device.

A semi-structured interview guide was used to guide the conduct of the interviews and to ensure that all topics of interest were discussed. The usability interviews consisted of two parts; during the first part of the interview, participants were asked open-ended questions, followed by specific probes about their overall experience of using the tablet device during the four week data collection period (see section 4.2), the timing of the COP-Q completion (see section 4.3), the visual aspects of the COP-Q (such as the layout of the questions, colour of text and background colour) (see section 4.4), and the technical aspects of the COP-Q and the tablet device (such as technical difficulties, charging the device, using the touch screen and helpfulness of the device training session) (see section 4.5) (approximately 10 minutes in duration).

During the second part of the interview, participants debriefed the COP-Q modules and the ‘patient facing text’ included in the pop-up boxes, and were also asked questions regarding their experience in accessing the ePRO and also the relevance of the COP-Q questions to their experience of COSP (see section 4.7). Participants also debriefed the PGI items (see section 4.8), and were given the opportunity to recommend any changes for either the ePRO and/or the tablet device (see section 4.9).

The second part of the interview was conducted using a think-aloud process where participants were asked to speak their thoughts aloud as they read and completed each item of the ePRO on the tablet device. This method allowed insight into patients’ thoughts as they completed the assessment, and thus identified any differences among participants in the way they understood and responded to the instructions, items, and response options of the ePRO.[^17^](#_ENREF_17)

- 1. **Ethical conduct**

This study was conducted in accordance with the principles outlined in the Declaration of Helsinki. All data was handled in accordance with privacy and data protection laws. The European Pharmaceutical Market Research Association (EphMRA) Code of Conduct was adhered to, and all participants provided written informed consent before completing any research activities and verbal consent at the beginning of the interview. Ethical approval was obtained from Alpha independent review board (IRB) (Reference number: NO8794D), and submission for ethical approval was made by the CRO, ORA Clinical.

### Subject data protection

All participants were provided with a paper ICF, which included a summary of the study and the study objectives, what the participant should expect if they consent to take part in the study (such as the study process, risk and benefits of participating and withdrawal from study), and how personal information would be stored and managed. A copy of the signed ICF was kept by each participant and clinical site. The ICFs were stored at the site securely, and a copy was retained by the patient only. The ICFs were not shared with AV or the study sponsor. ORA Clinical ensured to keep documentation of the appropriate completion and storage of all patient ICFs from the sites and shared written confirmation of this with AV. All paper files were stored in a locked file room at each site, and electronic and scanned versions of participants demographic and clinical information forms alongside with the ICFs completion confirmation, were shared with AV via a secure data-sharing platform (i.e., Box). This data was stored on a computer database, maintaining confidentiality in accordance with national data legislation. Study documents such as the CRF were not shared with AV until the ICF was signed by the participant.

Study data was de-identified; patients were assigned a unique patient ID number (see section 3.7.1) in place of their name and any identifiable data was redacted from interview transcripts – such as address, names, etc. This ID number was used to label the electronic and paper data files and documents relevant to each patient and was used to reference patient data in the reporting of the study findings (e.g., study reports, presentations, publications).

### Study withdrawal

Patients were informed when they were recruited, and at the beginning of the data collection period, that they were free to withdraw at any time without any penalty or loss of rights. Patients were informed when they were recruited that the CRO (ORA Clinical), AV PCO, the sponsor company (Novartis) or ethics committee have the right to stop their participation in the study at any time. This could be for one or more of the following reasons: if they do not keep appointments, if they arrive at a scheduled appointment unreasonably late, or if the sponsor company cancels the study. No participant withdrew from the study.

### Adverse Event (AE) Reporting

No solicited safety data capture is required for studies involving primary data collection without a Novartis drug of interest. However, if during the study, an adverse reaction was reported by a patient who happened to be receiving a Novartis product for their standard of care, this would need to be reported to Novartis as a spontaneous AE report, or to the local Health Authority if required by national regulatory requirements for individual case safety reporting.

Adverse reactions identified for non-Novartis products would also need to be reported to the local Health Authority in accordance with national regulatory requirements for individual case safety reporting or the Marketing Authorization Holder. All project team members who had the chance of identifying any AEs during the study completed Novartis’ adverse event reporting training prior to the study commencing.

No AEs were reported by any participant during the usability interviews, for either Novartis or non-Novartis products.

## Analysis

### Participant identification codes

Participants were provided with a unique identification (ID) code to anonymize their data. Figure 4 explains the format of the participants ID codes. The patient ID code contains information about the patient sex, age, underlying cause of COSP, COSP severity, site number, and participant number.


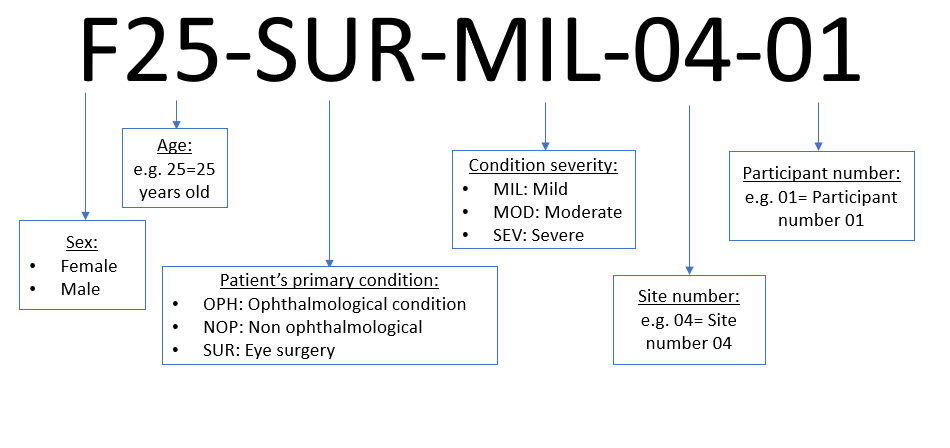


Figure 4. Example of participant ID code.

### Analysis of socio-demographic and clinical characteristics

Socio-demographic and clinical characteristics of the participants, collected from the demographic form and CRFs, were summarized descriptively. Ratio data (e.g., age) were summarized using totals (N values), means, and minimum/maximum (range) statistics. Similarly, categorical data (e.g., sex, ethnicity, race, education levels, work status) were summarized using totals (N values) to represent the sample in each sub-category. Clinical health information such as COSP severity, source of ocular pain, presence of other ophthalmological and non-ophthalmological diseases was summarized using totals (N values) and percentages to represent the sample in each sub-category. Descriptive data were summarized in tabular format.

### Analysis of the usability interview transcripts

Qualitative thematic analysis of verbatim transcripts for the usability interviews (N=10) was conducted, which involved sorting quotes by domain using Atlas.Ti software (Version 22).[^18^](#_ENREF_18)^,^[^19^](#_ENREF_19) The AV PCO project team reviewed the initial transcripts to familiarize themselves with the data and develop a coding scheme. The codes were developed based on the interview guide, to identify participants’ experience of using the ePRO device and their understanding of the COP-Q and PGI items.

Each transcript was assessed, and relevant passages of text were highlighted by assigning the relevant ‘code’. An iterative approach was taken to coding, with updates to the coding scheme being made throughout the coding process. As additional codes were added, previously coded transcripts were revisited and reviewed to identify any instances where the new code may apply, and any discrepancies were resolved through a discussion and consensus-building process.

The first two transcripts were coded by two members of the AV PCO team separately. The AV PCO project lead reviewed the analysis of the first interviews with the project researchers. Each transcript was then coded by one member of the research team.

Data was analyzed in Atlas.ti[^18^](#_ENREF_18)^,^[^19^](#_ENREF_19) using co-occurrence tables, which provided frequency counts of codes that co-occurred for each item property (for example, understanding and relevance of item) and item coded. For example, the number of participant quotes coded as ‘COPQ Eye Pain Frequency’ and ‘Understanding - Yes’, denotes the number of participants who understood the COP-Q Eye Pain Frequency item.

The data presented in this report provides frequency counts per property debriefed, alongside participant quotes to contextualize and illustrate the findings.

# Results

## Participant demographic and clinical characteristics

A total of 10 interviews with COSP patients in the US were conducted. Table 2 summarizes the demographic and clinical characteristics of the sample. Demographic characteristics (i.e., age, gender, race, ethnicity and education) were reported by the participants. The mean age of the total sample was 58 years (range: 33 to 79 years) and the majority of participants were female (n=6/10). Half the sample (n=5/10) described their race as White, four participants (n=4/10) as Black/African/Caribbean/Black British and one participant (n=1/10) as multi-racial. All participants (N=10) described their ethnicity as ‘non-Hispanic, non-Latino or of non-Spanish’ origin. In terms of education, the majority of the sample (n=8/10) completed more than high school, and the remaining two participants (n=2/10) completed high school or less.

The clinical characteristics summarized include the underlying cause of participant’s COSP, the type(s) of corneal surgery (if, any) participants had, the severity of COSP, and the ophthalmological condition(s) they have. These clinical characteristics were reported by the recruiting HCP/clinician, based on participants’ medical history.

Half of the sample (n=5/10) was reported to have ophthalmological conditions as the underlying cause of COSP, two participants (n=2/10) were reported to have non-ophthalmological conditions, and three participants (n=3/10) were reported to have surgical causes of COSP.

The majority of the participants (n=7/10) had not had corneal surgery. Two participants (n=2/10) were reported to have cataract surgery, two participants (n=2/10) were reported to have PCIOL insertion, and one participant (n=1/10) was reported to have LASIK surgery. Some participants had more than one surgery. Severity of participants’ COSP was rated by the recruiting HCP/clinician at the clinical site. Only two participants (n=2/10) had severe COSP, three had moderate COSP, and half of the sample (n=5/10) had mild COSP. All participants (N=10) had DED, one participant (n=1/10) had Blepharitis/Meibomitis and one participant (n=1/10) had MGD, as per HCP/clinican report. Some participants had more than one ophthalmological condition.

| Table 2. Demographic and clinical characteristics (N=10). | |
| --- | --- |
| Demographic characteristics (as reported by participants) | **Total (N=10)** |
| Age (n%)  Min, Max  Mean | 33,79  58 |
| Sex (n%)  Female  Male | 6 (60%)  4 (40%) |
| Race (n %)  White  Black/African/Caribbean/Black British  Multi-racial | 5 (50%)  4 (40%)  1 (10%) |
| Ethnicity (n%)  Non-Hispanic, Non Latino or Non-Spanish origin | 10 (100%) |
| Education (n%)  More than high school  High school or less | 8 (80%)  2 (20%) |
| Clinical characteristics (as reported by recruiting HCP/clinician) | **Total (N=10)** |
| Describing the patient (n%)  Ophthalmological  Non-Ophthalmological  Surgical | 5 (50%)  2 (20%)  3 (30%) |
| Form of corneal surgery (n%)*  No surgery  Cataract  PCIOL insertion  LASIK  **More than 1 surgery was selected* | 7 (70%)  2 (20%)  2 (20%)  1 (10%) |
| Severity of COSP (n%)  Severe  Moderate  Mild | 2 (20%)  3 (30%)  5 (50%) |
| Ophthalmological condition (n%)*  Dry eye disease (DED)  Blepharitis/Meibomitis  MGD  *More than 1 condition was selected | 10 (100%)  1 (10%)  1 (10%) |

## General usability of the tablet device

### Overall experience of tablet device

The first part of the interview explored participants’ overall experience of using the tablet device during the four-week data collection period. All participants (N=10/10) were asked if there were any specific aspects that they liked or disliked about the tablet device and if they would recommend any changes. Of note, all participants (N=10/10) reported the tablet device was easy to use.

*“Uh, well it was very easy to use. It was setup everything already for me, so, um, you know, there wasn’t a lot of instructions, which was good.”* (M56-OPH-MIL-01-07)

*“Um, it was easy to use. Um, the questions were easy to understand. And, um, it didn’t take long to complete the questions.”* (F64-OPH-MOD-01-01)

#### Likes

Over half of participants (n=6/10) reported a specific aspect of the tablet device they liked during the four-week data collection period (see Table 3 for detail). The most frequently reported aspects that participants liked about the tablet device were the alarms (n=2/6) and the instructions, specifically that these were concise and very easy to follow (n=2/6). Of note, two participants (n=2/6) reported more than one aspect they liked. Four participants (n=4/10) did not specify the aspects they liked the most about the tablet device.

| **Table 3. Tablet device aspects that participants liked (N=10).** | | |
| --- | --- | --- |
| Likes | Key findings | Example quotes |
| Alarms | - 2/6 participants reported that they liked the tablet device alarms. | *“It was great. Simple, the alarms were wonderful.”* (F54-OPH-MIL-01-08) |
| Instructions | - 1/6 participants (M56-OPH-MIL-01-07) reported that the instructions were concise. - 1/6 participants (M54-SUR-MOD-01-02) reported that the instructions were easy to follow. | *“Uh, well it was very easy to use. It was setup everything already for me, so, um, you know, there wasn’t a lot of instructions, which was good. Um, and, um, I think it was—it went fairly well.”* (M56-OPH-MIL-01-07) |
| Responsive interface | - 1/6 participants (M54-SUR-MOD-01-02) reported that the tablet’s interface was very responsive. | *"The, the tablet itself was, um, very, um—you know, had a nice, tactile, you know, feel to it. The buttons were responsive and easy to press.”* (M54-SUR-MOD-01-02) |
| Navigation | - 1/6 participants (F79-SUR-MIL-01-03) reported that the navigation on the tablet was easy. | *“I had no issues with the tablet…It was easy to navigate.”* (F79-SUR-MIL-01-03) |
| Large formatting | - 1/6 participants (M33-OPH-MIL-01-04) reported that the tablet’s overall formatting was quite large which made it easy for them to navigate. | *“Um, everything was pretty large. The, you know, the formatting of everything was large and clear, so, um, it was easy to see and easy to use and easy to click on, so I didn’t really have too many issues as far as the, uh, interface of it.”* (M33-OPH-MIL-01-04) |
| Long response time windows | - 1/6 participants (F54-OPH-MIL-01-08) reported that the response time windows were long. | *“…Um, the timeframes were easy 'cause they were large windows.”* (F54-OPH-MIL-01-08) |

#### Dislikes

Half of the participants (n=5/10) reported aspects that they disliked during the four-week data collection period (see Table 4 for detail). The most frequently reported aspect that participants disliked was the pin code sometimes not functioning properly (n=2/5). Of note one participant (n=1/5; F55-NOP-SEV-01-06) reported more than one aspect they disliked. Three participants (n=3/5) who expressed dislikes also recommended some changes to make to the tablet device (see section 4.9). Five participants (n=5/10) did not report any dislikes.

| **Table 4. Tablet device aspects that participants disliked (N=10).** | | |
| --- | --- | --- |
| Likes | Key findings | Example quotes |
| Pin code not functioning properly | - 2/5 participants reported they disliked that the pin code they used to log into the questionnaires sometimes didn’t work on the first attempt. | **Interviewer: *“Great. Thank you. And, um, what did you like or dislike about the tablet device?”*** *“Um, sometimes I had to—I would enter my pin number and it would say in—the pin number wasn’t correct and I would have to re-enter it. And I didn’t understand when it was the same number every time.”* (F64-OPH-MOD-01-01) |
| Alarm not functioning properly | - 1/5 participants (F55-NOP-SEV-01-06) reported that the alarm would not work sometimes. | *“Um, it was okay using it, but the alarm didn’t work, so I missed a couple, couple, um, sessions because the alarm didn’t go off. It only went off for one day. And like coming in late, I missed it I think twice in the evening because I got off work, went to the grocery store, came back, and forgot. So it would be like the alarm is the main issue that I had.”* (F55-NOP-SEV-01-06) |
| Tablet device was heavy | - 1/5 participants (M33-OPH-MIL-01-04) reported that the tablet device was heavy. | **Interviewer: *“And, um, was there anything that you disliked about the tablet device?”*** *“Um, honestly no, not that I can think of. I mean it could be maybe a little more lightweight. It was heavy. But other than that, no.”* (M33-OPH-MIL-01-04) |
| Tablet device was big | - 1/5 participants (F55-NOP-SEV-01-06) reported they disliked the size of the tablet device as they felt it was too big to carry with them. | *“Also if, also if it was like a smaller—not, not the big tablet, like a small tablet like the size of a, um, a cell phone, then it would have been easier because I could carry it with me.”* (F55-NOP-SEV-01-06) |
| Questionnaires took longer time to complete on some days | - 1/5 participants (F49- NOP-SEV-01-05) reported that on some days it would take longer than usual to complete the questionnaires. | *“Um, the only thing that I disliked about it was I thought the questions would be the same or similar every day and every once in a while I think it seemed like, um, once a week or so or every two weeks, um, the questions were slightly different. So where I expected it to be quicker, it was actually a little bit longer. So that's the only thing I disliked.”* (F49- NOP-SEV-01-05) |

## Timing for completion of the ePRO

Participants were asked about their experience of completing the ePRO in regard to timing. Participants were specifically asked about the following: their ability to complete the ePRO alone at home, fitting the ePRO into their daily schedule and completing this twice a day, the overall time it took them to complete the ePRO, whether they missed any sessions, their preference on recall period (4-hour recall period/24-hour recall period), whether they needed to take a break while completing the ePRO, and their experience of the daily alarms/reminders.

### Completion of the ePRO

All participants (N=10/10) reported that they were able to complete the ePRO alone at home and that it was easy fitting the task twice a day into their daily schedule (N=10/10).

**Interviewer: *“Okay. Thank you. And, um, and were you able to complete the questionnaires on the device by yourself whilst at home?”*** *“Yes. I was able to do all the questionnaires by myself.”* (F64-OPH-MOD-01-01)

**Interviewer: *“Great. Thank you. And, um, how easy or difficult was it to fit completing the questionnaire twice a day into your daily routine?”*** *“Uh, very easy. Uh, I set an alarm on the device and, um, also on my phone.”* (M56-OPH-MIL-01-07)

### Time taken to complete the ePRO

Participants were asked (N=10/10) to estimate the approximate time it took them to complete the ePRO daily. The reported duration for each participant varied between a few minutes to five minutes maximum (n=5/10), six to eight minutes maximum (n=2/10) and 10 to 15 minutes maximum (n=3/10), indicating that completion of the ePRO was generally a quick task.

**Interviewer: *“And approximately how long did it take you each day to complete the questionnaires?”*** *“Um, depending on the, the length of the questionnaire, I would say five minutes and under.”* (M56-OPH-MIL-01-07)

### Preference on recall period of the Symptom Module

All participants were asked about their preference on having to complete the COP-Q twice a day (4-hour recall period) or if they preferred completing the COP-Q once a day (24-hour recall period) during the four-week data collection period. Participants responses were divided equally, with half of them (n=5/10) reporting that they preferred completing the COP-Q twice a day as it was easier to remember and the other half (n=5/10) reporting that they preferred completing the COP-Q once a day as it was less bothersome (see Table 5 for detail).

| **Table 5. Recall period preference (N=10).** | | |
| --- | --- | --- |
| Recall period | Key findings | Example quotes |
| Completing the COP-Q twice a day (4-hour recall period Symptom Module) | - 5/10 participants reported they preferred completing the questionnaires twice a day as it was easier to recall their symptoms over the past 4-hours. | *"Um, I think I liked it with the past four hours… Um, because it was easier to recall what was happening in the last four hours than in 24 hours.”* (F64-OPH-MOD-01-01) |
| Completing the COP-Q once a day (24-hour recall period Symptom Module) | - 5/10 participants reported they preferred completing the questionnaires once a day as this was less bothersome and time consuming. | *“Um, probably once a day…Um, well it's, it's easier to answer like for the, the 24 hours 'cause you're doing the four hours and then it goes to 24. So it's like, okay, so how far do I go back. Um, and it was just—it wasn’t confusion…”* (F55-NOP-SEV-01-06) |

### Need to take breaks

All participants (N=10/10) reported that they did not need to take breaks while completing the ePRO during the four-week data collection period.

**Interviewer: *“…did you ever need to pause or take a break whilst completing the questionnaires?”*** *“No. They were so short that I was able to complete them in, in one sitting.”* (M54-SUR-MOD-01-02)

### Time windows

All participants (N=10/10) were also asked about the time windows they had to complete the ePRO within in the morning (7:00am to 10:00am) and in the evening (18:00pm to 23:00pm). Most participants (n=7/10) reported that the time windows were easy to comply with.

*“Um, I think that was good. Uh, you know, it's—it gives you plenty of time to, to sit and get them done and work it around your schedule.”* (M56-OPH-MIL-01-07)

One participant (n=1/10; F55-NOP-SEV-01-06) reported that the time windows were not easy to comply with. This participant would have preferred the ability to set the time windows on their own instead of complying with the default ones, as this would fit better within their daily schedule.

**Interviewer: *“Thank you. And, um, what did you think about the time windows that you had to complete the questionnaires within?”*** *“Um, not good because everybody is not on the same schedule, so it's like, um, I, I think this…Like set the, the schedule instead of it already being set.”* (F55-NOP-SEV-01-06)

It was unclear if the remaining two participants found the time windows easy or difficult to comply with (n=2/10) as they did not provide a clear answer when asked. However, one of these participants (n=1/2; F79-SUR-MIL-01-03) mentioned that the time window in the evening was larger (18:00pm to 23:00pm) than the one in the morning (7:00am-10:00am) and inferred that it was easier to complete the questionnaire within the longer timeframe. The other participant (n=1/2; M33-OPH-MIL-01-04) mentioned that the time windows should be of the same duration for the morning and the evening assessments to avoid confusion. Of note, this was also mentioned by another participant (n=1/10; F64-OPH-MOD-01-01; see section 5.3.7).

*“Uh, the only thing that I noticed, and this might—is that for the morning questions, I had three hours. I had to complete the questions in, you know, that three-hour window. But the evenings, there was a much—there was a larger window timeframe to complete the questions. And I don’t—I'm just curious as to why that was.”* **Interviewer: *“Okay. Thank you. And did you find that it was easy to complete them when you had a longer timeframe or did you prefer completing them in the morning?”*** *“I guess easier because of the timeframe.”* (F79-SUR-MIL-01-03)

*“Um, I think the ending hours should be like consistent. So like instead of it ending at 10:00 a.m. and 11:00 p.m., it should both end at 10:00 a.m.—10:00 p.m. or 11:00 a.m. and 11:00 p.m. just so that eliminates a little bit of confusion.”* (M33-OPH-MIL-01-04)

Three participants recommended changes for the time windows in which the questionnaires had to be completed (n=3/10) (please see section [5.9](#_Toc137131479)).

### Missed questionnaires

All but one participant was asked if they had missed completion of any of the ePRO entries during the four-week data collection period (n=9/10). The majority of participants asked reported that they had missed at least one diary session (n=8/9), while one participant reported that they did not miss any during the data collection period (n=1/9; F74-SUR-MOD-01-09). One participant (n=1/10; M56-OPH-MIL-01-07) was not asked about missed questionnaires due to time constraints during the interview. Reasons for missed completion of the ePRO are summarized in Table 6.

| Table 6. Missed diary entries (N=10). | | |
| --- | --- | --- |
| Reasons for missing entries | Key findings | Example quotes |
| Family commitments/emergency | - 3/9 participants reported they missed a diary entry due to family commitments/emergency. | *"Uh, I think one time it did because I wasn’t at home with the device, so I wasn’t able to complete the questionnaire one particular night. I had a family event.”* (F79-SUR-MIL-01-03) |
| Forgot to complete | - 3/9 participants reported they missed diary entries because they forgot to complete them. Of note, one participant (F64-OPH-MOD-01-01) reported they missed a second diary entry without realizing as they thought they had previously completed it. | *“Well there was one morning I just forgot... And then there was another day where I thought I had completed all the questionnaire, I had answered that morning. And then I got a call that said, you didn’t finish the questionnaire…So I don’t know if I just didn’t hit the right button or what happened, but I missed that. But otherwise, I completed them all.”* (F64-OPH-MOD-01-01) |
| Overslept | - 1/9 participants (M54-SUR-MOD-01-02) reported that they overslept one morning and therefore they missed one morning diary entry. | *“And it was of course it's a national holiday. I didn’t wake up until about three minutes after ten, so I guess I slept through the alarm in my other room and when I woke up about three minutes after ten, I'm like, oh my gosh…Of course it was already after ten and I had missed it”* (M54-SUR-MOD-01-02) |
| Alarm volume too low | - 1/9 participants (F49-NOP-SEV-01-05) reported that they missed a diary entry at the beginning of the data collection period because the alarm volume was too low, and they didn’t hear the notification. | *“The day actually after I got the tablet, because the volume, um, was too low... So the next morning, I didn’t hear it when it rang to alert me to use it. So I missed that day. But after that, I was fine. I got it every single day.”* (F49- NOP-SEV-01-05) |
| Alarm did not go off | - 1/9 participants (F55-NOP-SEV-01-06) reported that they missed a couple of diary entries because the tablet device alarm did not go off. | *“...but the alarm didn’t work, so I missed a couple, couple, um, sessions because the alarm didn’t go off. It only went off for one day. And like coming in late, I missed it I think twice in the evening because I got off work, went to the grocery store, came back, and forgot.”* (F55-NOP-SEV-01-06) |

### Alarms/reminders

All but one participant (n=9/10) were asked about the daily alarms/reminders. Most participants asked (n=6/9) reported that the daily alarms/reminders of the tablet device were very helpful in reminding them to complete the questionnaires on a daily basis.

*“…I mean if there weren't any alarms, I might have forgotten to do the questionnaire, but I actually, um, like that they had the alarms in place.”* (F49- NOP-SEV-01-05)

However, three participants reported that they experienced some issues with the alarms/reminders (n=3/9). Of note, all three participants (n=3/3) reported that the alarm did not always go off.

*“…but the alarm didn’t work, so I missed a couple, couple, um, sessions because the alarm didn’t go off.”* (F55-NOP-SEV-01-06)

One of these participants (n=1/3; M54-SUR-MOD-01-02) also mentioned that the alarm would not go off the time that they set it for, and that noted that this happened a couple of times.

*“Um, I, I did notice that it seemed like when you set the alarm that the alarm didn’t always go off at the time you set it for…And that seemed like it—I, I can think, you know, if it happened once, maybe it was just a one-time thing, but it seemed, it seemed to happen a couple of times…”* (M54-SUR-MOD-01-02)

Another participant (n=1/3; F79-SUR-MIL-01-03) reported that the alarm wasn’t reliable, as sometimes it wouldn’t go off and at other times the volume of the alarm would be too low.

*“Uh, that was, uh, not a reliable alarm. Uh, it could be the way it was set. I don’t know. But it wasn’t reliable. And, uh, some days I never—I'm not going to say it didn’t go off, but some days I did not even hear the alarm.”* (F79-SUR-MIL-01-03)

Two participants (n=2/10) recommended changes for the alarms/reminders (please see section section 4.9).

## Visual aspects of the ePRO

All participants (N=10/10) were also debriefed on the visual aspects of the ePRO. In general, all participants (N=10/10) were happy with the layout, colour of the text, and colour of the background on the tablet device and reported that it was easy to navigate the questionnaires and to read and answer the questions (see Table 7 for further detail).

Although all participants (N=10/10) reported that they liked the visual aspects of the tablet and the ePRO, three participants (n=3/10) made recommendations for potential improvements to the visual aspects of the ePRO (please see section [5.9](#_Toc137131479)).

| **Table 7. Visual aspects of the ePRO (N=10).** | | |
| --- | --- | --- |
| Item properties | Key findings | Example quotes |
| Layout of the screen | - 10/10 participants reported that the layout of the screen was appropriate as they found it easy to navigate through the questionnaires and the response options. | *"I think it was a very good layout. I think it made it very easy to find everything. Made it very easy to, to see which, you know, everything was all in order.”* (M56-OPH-MIL-01-07) |
| Colour of the text | - 10/10 participants reported that the colour of the text was appropriate as all questions were clear to read. | *“Uh, that was fine. It was easy to read, easy to see…and to respond.”* (M62-OPH-MIL-01-10) |
| Background colours | - 10/10 participants reported that the background colours were appropriate as they could read the text clearly. | *“Yeah. Uh, again whatever colour scheme or palette that was used I found it, um, it was not distracting. It made the text and questions easy to read.”* (M54-SUR-MOD-01-02) |

## Technical aspects of the ePRO

### Technical difficulties

All participants (N=10/10) were asked if they had experienced any technical difficulties while using the tablet device during the four-week data collection period. Half of the participants (n=5/10) experienced at least one technical issue using the tablet device.

Specifically, two participants (n=2/5) reported issues when entering the pin code to log in to the device as it did not always work the first time (please see section 4.2.1). One participant (n=1/5; F55-NOP-SEV-01-06) reported that the alarms did not always go off at the time that they had set them, resulting in them missing completion of the questionnaires a couple of times (please see section 4.3.7).

Another participant (n=1/5; M62-OPH-MIL-01-10) reported that the tablet would reset itself or that they would have to reboot it themselves sometimes.

*“Uh, I found it, you know, very easy to do so. Uh, um, sometimes it would, uh, reset itself or, or I guess I had to reboot it. That happened two or three times. Um, but otherwise, um, very much like an iPad. It was, it was fairly simple and fairly easy to, to utilize.”* (M62-OPH-MIL-01-10)

One participant (n=1/5; M54-SUR-MOD-01-02) reported that when they received the tablet, they didn’t realize that they had to sync the tablet with Wi-Fi/internet, and as a result they thought that they missed a diary entry.

*“And then I realized that you have to setup your wireless access to sync with the tablet. And once I figured out that that had to be done, it worked flawlessly from then on. So for anyone else who's not, you know, computer literate or might not think about it, it might be helpful to have a, a basic set of instructions of how to set it up at the house, even if—even in terms of simple like, you know, make sure that your Wi-Fi, you know, can connect with the tablet, something like that.”* (M54-SUR-MOD-01-02)

Of note, this should have not led to missing diary entries as the device would automatically update the entries once connected to the internet. As it was only one participant who reported confusion with syncing the tablet with their internet and given that a user guide was provided to the participants when they enrolled in the study explaining the process of connecting the tablet device to the internet and this was also mentioned in the tablet device training session provided by the sites, this is not an area of major concern.

### Charging tablet device

All participants asked (N=10/10) reported that charging the tablet devices at home was easy.

*“Yeah. Extremely easy. I had it sitting right next to my desktop Windows computer, which has a USB port on the front. So I literally would set the tablet next to it and, uh, you know, charge it when needed.”* (M54-SUR-MOD-01-02)

### Touch screen

Participants were also asked (N=10/10) about their experience with the tablet device touch screen. Most participants reported that the touch screen was very easy to use, and it responded quickly with the first touch/try (n=9/10).

*“It was easy. Um, it's a large touch screen, so that made it easy.”* (M33-OPH-MIL-01-04)

Only one participant (n=1/10; M54-SUR-MOD-01-02), reported that sometimes they had to press the screen for a few times in order to record their responses, however they mentioned that they weren’t sure if this happened because they did not press the response buttons firmly enough or if there was a fault with the tablet device’s touch screen.

**Interviewer: *“And did the tablet always respond to your selection first time when you would select an answer?”*** *“Uh, yeah. That's, that's a good question. Um, I would say, hmm, I'm going to say 80% of the time… sometimes, you know, I might feel like it was a bit delayed or, or not responsive, but it could have just been that I had not pressed it as firmly or specifically as I thought I had.”* (M54-SUR-MOD-01-02)

### Tablet device training session

All participants (N=10/10) reported that the training session provided on how to use the tablet device was helpful.

**Interviewer: *“And was the training session on how to log-in to the tablet device and access the questionnaire helpful?”*** *“Yes. Extremely. Uh, because they were able to, you know, show me, you know, let's say that the, the actual questionnaire somehow closes. They were actually able, they were able to show me on the device itself, kind of like the device's desktop which icon that I would choose to start it back up again. So that was a good process that they walked through with me in setting up the pin in the, in the doctor's office.”* (M54-SUR-MOD-01-02)

## Accessing the ePRO

Participants were asked about their experience of turning on the tablet device, the login process and accessing the ePRO. Two participants (n=2/10) recommended changes to be made to improve the accessibility of the questionnaire (please see section 4.9).

#### Turning on the tablet device and locating the questionnaires

All participants asked about the process of turning on the tablet device and locating the required ePROs for completion during each session, reported that this was an easy task to do (n=6/10). Four participants (n=4/10) were not debriefed on this.

*“It was very easy. I mean it, it pretty much just presented itself, so, um, I had no difficulties, yeah.”* (M33-OPH-MIL-01-04)

#### Login process

Participants were asked about the login process (N=10/10) and loading the questionnaires. Most participants asked (n=8/10) reported that the login process to the ePRO during the four-week data collection period was easy and straightforward.

*“Not difficult. Was easy…It was very straightforward… I would not change anything.”* (F79-SUR-MIL-01-03)

Just two participants (n=2/10) reported having difficulties with the login process during the four week data collection period. One participant (n=1/2; F64-OPH-MOD-01-01) reported that their tablet would sometimes take time to synchronize, and when this happened, they would have to enter their pin code a few times for it to work.

*“Um, the synchronization took a while. Like I just tried to log-in and it said, you know, wrong pin number.”* (F64-OPH-MOD-01-01)

The other participant (n=1/2; F74-SUR-MOD-01-09) reported that their pin code did not always work; however, they mentioned that this only happened at the beginning of the study because they had forgotten their code. This issue was resolved once they requested a new pin.

**Interviewer:** ***“Did you have any difficulties at all?”*** *“I did when I first started because I had forgot my code, but after then, you know, after I remembered everything, so I didn't have no problem with that.”* (F74-SUR-MOD-01-09)

#### Active sessions

All participants (N=10/10) reported that it was easy and clear to know which questionnaires they had to complete in each time window during the four-week data collection period.

**Interviewer: *“And was it clear which session was active and which was not?”*** *“Yes, it was…You couldn’t do it if it wasn’t active.”* (F49- NOP-SEV-01-05)

#### Loading questionnaires

All participants (N=10/10) reported that the questionnaires would load easily and quickly during the four-week data collection period.

*“Uh, extremely easy because everything was listed sequentially.”* (M54-SUR-MOD-01-02)

One participant (n=1/10; F74-SUR-MOD-01-09) mentioned that it was easy to load the questionnaires during the four-week data collection period, although noted that sometimes they would lose the screen they had been looking at and had to return to the main screen to reload it. It wasn’t clear, however, if this was an error caused by the tablet’s software or due to misuse by the participant.

*“It was easy, but then sometimes the screen, I would lose it, then I’d have to go back and log back in, but you know, and that may be the way I was holding it, but I didn't have that much problem with that.”* (F74-SUR-MOD-01-09)

#### Getting to the next screen

All participants asked (n=9/10) reported that it was easy and clear what to do if they wanted to get to the next screen of the ePRO during the four-week data collection period. One participant (n=1/10; F54-OPH-MIL-01-08) was not debriefed on this topic.

**Interviewer: *“And, um, is it clear how you would get to the next screen?”*** *“Yes. It says next at the bottom.”* (F49-NOP-SEV-01-05)

## Debriefing of the COP-Q items and ‘patient facing text’

### COP-Q title page

Participants were presented with the COP-Q title page and were debriefed on accessing and loading the COP-Q on the tablet screen, reading the text and navigating to the next screen (Appendix A). Overall, participants reported that it was easy to access and load the COP-Q on the screen (n=8/10), easy to read the text (n=8/10) and that it was clear what to do to get to the next screen (n=8/10) (see Table 8 for further detail).

| Table 8. COP-Q title page key findings (N=10). | | |
| --- | --- | --- |
| Item properties | Key findings | Example quotes |
| Accessing and loading COP-Q | - 8/10 participants reported it was easy to access and load the COP-Q on the tablet device. - 2/10 participants were not asked whether it was easy to access and load the COP-Q. | **Interviewer: *“Um, so how easy or difficult did you find it to access and load this screen during the four-week period?”*** *“Easy. Very easy.”* (F79-SUR-MIL-01-03) |
| Navigating to the next screen | - 8/10 participants reported it was clear how to navigate to the next screen. - 2/10 participants were not asked whether it was clear how they would get to the next screen. | **Interviewer: *“And is it clear how you can get to the next screen?”*** *“Yes. It says next at the bottom.”* (M62-OPH-MIL-01-10) |
| Reading text | - 8/10 participants reported it was easy to read the text presented on the COP-Q title page. - 2/10 participants were not asked whether the text was easy to read. | *“... Uh, easy because it's, uh, you know, black background and white lettering, so it's clear and, yeah, clear and easy.”* (F79-SUR-MIL-01-03) |

### COP-Q Eye Pain Severity Module

The COP-Q Eye Pain Severity Module is a single item designed to capture patients’ perception of eye pain severity during the ‘past 4 hours’. Patients respond to the item using a 0–10 numerical rating scale (NRS) ranging from ‘No eye pain’ (0) to ‘Worst possible eye pain’ (10).

Overall, participants reported that it was easy to read the text (N=10/10), understand the item (n=9/10), select a response (n=8/10), think back over the specified recall period (n=7/10) and that the response options were appropriate (n=7/10). Not all participants were debriefed on these properties due to time constraints. Please see Table 9 below for more details and example quotes. Four participants (n=4/10) recommended changes to be made to this module. These are summarized in section 4.9).

| Table 9. Eye Pain Severity Module key findings (N=10). | | | |
| --- | --- | --- | --- |
| Item | Item property | Key findings | Example quotes |
| Eye Pain Severity  Please rate the severity of your eye pain at its worst over the past 4 hours:  Participants respond to the item using a 10-point numerical rating scale (NRS), ranging from ‘no eye pain’ (0) to ‘worst possible eye pain’ (10) | **Reading text and font size** | - 10/10 participants reported that the text was easy to read. - 8/10 participants reported that the font size was large enough to read. - 1/10 participant (M33-OPH-MIL-01-04) reported that the font size could be larger, although noted that they had no issues reading the text. - 1/10 participant (M56-OPH-MIL-01-07) was not asked whether the font size was large enough. | **Interviewer: *“And how easy or difficult do—did you find it to read and answer that question?”*** *“Extremely easy.”* (M54-SUR-MOD-01-02)  *“Um, it's very easy to answer the question. Um, it's pretty easy to read it as well. Um, maybe make it a little larger. I don’t know. I'm thinking a little larger.”* (M33-OPH-MIL-01-04) |
|  | **Understanding** | - 9/10 participants reported that the item was easy to understand. - 1/10 participant (M54-SUR-MOD-01-02) was not asked whether the item was easy to understand. | *“Um, I think pretty easy. It's self-explanatory... I think it's very understandable.”* (M56-OPH-MIL-01-07) |
|  | **Selecting a response** | - 8/10 participants reported that selecting a response was easy. - 1/10 participant (F55-NOP-SEV-01-06) reported that selecting an answer was sometimes hard. - 1/10 participant (M62-OPH-MIL-01-10) was not asked about ease of selecting a response. | **Interviewer: *“And how easy or difficult was it to select an answer for that question?”*** *“Um, well sometimes it was a little hard. I had to kind of guess. Um, like how it says the, the worst possible eye pain. So I mean it's just, I don’t know...Sometimes it's, it's just kind of hard to figure out which one.”* (F55-NOP-SEV-01-06) |
|  | **Recall period** | - 7/10 participants reported that the recall period was easy to think back over. - It was not clear whether 2/10 participants found the recall period easy or difficult to think back on. - 1/10 participant (M54-SUR-MOD-01-02) was not asked about ease of recall. | **Interviewer: *“And how easy or difficult it was to think about your eye pain over the past four hours****?” “Um, very easy.”* (F49-NOP-SEV-01-05) |
|  | **Response options** | - 7/10 participants reported that the response options were appropriate. - 1/10 participant (F55-NOP-SEV-01-06) reported that they found it sometimes difficult to know which response option to select and having the response options written out (e.g., no pain, a little pain etc.) instead of the numeric values, might have made the selection of responses easier for them. - 2/10 participants were not asked about the appropriateness of the response options. | *“That's, that's the tricky part because it's like, okay, I have eye pain, but to rate it on a scale from one to ten, sometimes it's like a little hard to figure out which one, which one it is… I mean if it's like some—if instead of the numbers like some eye pain, a lot of eye pain, like, like that instead of the numbers.”* (F55-NOP-SEV-01-06) |

### COP-Q Eye Pain Frequency Module

Participants were debriefed on the COP-Q Eye Pain Frequency Module, a single item designed to capture the frequency of patients’ eye pain within the ‘past 24 hours’. Patients respond to the item using a five-point verbal descriptor scale, ranging from ‘None of the time’ (0) to ‘All of the time’ (4).

Overall, all participants reported that it was easy to read the text and select an answer (N=10/10 each), that they understood the item (n=9/10), that the response options were appropriate (n=7/10) and that the recall period was easy to think back over (n=6/10). Not all participants were debriefed on these properties due to time constraints. Please see below Table 10 that summarizes the key findings. Three participants (n=3/10) recommended changes to this module. These can be found in section 4.9.

| Table 10. Eye Pain Frequency Module key findings (N=10). | | | |
| --- | --- | --- | --- |
| Item | Item property | Key findings | Example quotes |
| Eye Pain Frequency  How much of the time have you had eye pain over the past 24 hours?  Participants respond to the item using a 10-point numerical rating scale (NRS), ranging from ‘none of the time’ (0) to ‘all of the time’ (10) | **Reading text and font size** | - 10/10 participants reported that the text was easy to read. - 3/10 participants reported that the font size was large enough. - 7/10 participants were not asked about the font size. | **Interviewer: *“Okay. And, uh, could you please tell me again how easy or difficult it was to read this question?”*** *“Uh, very easy.”* (M62-OPH-MIL-01-10)  **Interviewer: *“Great. And, um, do you think the font size is large enough?”*** *“Yes, I do.”* (F64-OPH-MOD-01-01) |
|  | **Understanding** | - 9/10 participants reported that the item was easy to understand. - 1/10 participant (M62-OPH-MIL-01-10) was not asked whether the item was easy to understand. | *“… I think it's, um, very readable and very easy to understand.”* (M56-OPH-MIL-01-07) |
|  | **Selecting a response** | - 10/10 participants reported that selecting a response was easy. | **Interviewer: *“Great. And how easy or difficult was it to select an answer for that question?”*** *“Very easy.”* (F54-OPH-MIL-01-08) |
|  | **Recall period** | - 6/10 participants reported that the recall period was easy to think back over. - 2/10 participants reported that the recall period was difficult to think back on. - It was unclear if 1/10 participants (F79-SUR-MIL-01-03) found the recall period easy or difficult to think back on. - 1/10 participant (M54-SUR-MOD-01-02) was not asked about ease of recall period. | **Interviewer: *“How easy or difficult was it to think about how much of the time you've had eye pain over the past 24 hours?”*** *“Very easy.”* (F49- NOP-SEV-01-05)  *“I think that was a little bit more difficult than the past four hours.”* (F64-OPH-MOD-01-01) |
|  | **Response options** | - 7/10 participants reported that the response options were appropriate. - 1/7 participant (M33-OPH-MIL-01-04) however reported that the response options were ambiguous and that it would be helpful to add time specific recall periods that participants would have to think back at for each response option. - 3/10 participants were not asked about the appropriateness of the response options. | *“Uh, I think they give you, um, enough information that you should be able to answer easily and, and it makes it where it gives you a lot of different things. So I, I think they're good.”* (M56-OPH-MIL-01-07)  *“Um, they're very—they're a little ambiguous. Maybe a little subjective. But at the same time, it kind of covers all your bases too, so sorry…Maybe keep these answers like they are, but maybe like put in parentheses like all of the time it's like in that 24 hours, is that like at least 20 plus hours. Like is a little of the time two hours a day at least? Like what is some of the time, like kind of clear it up a little bit there.”* (M33-OPH-MIL-01-04) |

### COP-Q Symptom Module (4-hour recall period)

The COP-Q Symptom Module consists of seven items which assess symptoms associated with COSP. All items have a 0–10 NRS, ranging from not experiencing the symptom at all (0) to experiencing the symptom at its worst (10) (e.g., the response scale for ‘eye irritation’ is ‘No eye irritation’ [0] to ‘Worst possible eye irritation’ [10]). Two different recall period versions of the COP-Q Symptom Module have been developed (a 24-hour recall period version and a 4-hour recall period version), to compare these versions in terms of measurement properties and score comparability.

The majority of the participants found the item easy to read and understand and were able to select an appropriate response option for each item in the module. Due to time constraints, not all participants were debriefed on each item and item property. Please see Table 11 which summarizes the key findings for the 4-hour recall period Symptom Module.

Three participants (n=3/10) recommended changes that could be made to specific items within the Symptom Module. These are detailed in section 4.9.

#### 4-hour recall period

Participants (n=4/10) reported that it was easy to select a response option thinking back over the past 4 hours.

**Interviewer: *“And was it easy to recall an answer about your eye pain at its worst over the past four hours?”*** *“It was, especially as I mentioned in the morning because I would do this very soon after, you know, when I awoke. And I generally have, you know, minimal eye pain, you know, in the morning compared to the rest of the day. So doing this answer—doing the answers in the morning, typically was—you know, there wasn’t much to think about in general and that's why my scores are generally low.”* (M54-SUR-MOD-01-02)

One participant (n=1/10; F74-SUR-MOD-01-09) noted that it would have been easier to recall answers within a 24-hour period, rather than a 4-hour period, particularly when thinking back over the past 4-hours early in the morning. This participant noted that they would not have any eye pain, just a little irritation early in the morning, making it challenging to respond to the items.

*“I would, would change some of it, because it says the past 24 hours, you know, over the past 4 hours, with your eye pain, so you know, you’re not having too much eye pain until you’re up focused and see what’s going on in the morning times, and at 7 o’clock, you know, you might have just a little bit of irritation, but you won’t have eye pains until you really focus on what you are doing.”* (F74-SUR-MOD-01-09)

Five participants (n=5/10) were not asked whether it was easy to think back over a 4-hour recall period.

| Table 11. COP-Q Symptom Module (4-hour recall period) key findings (N=10)4-hour recall period). | | | |
| --- | --- | --- | --- |
| Symptom 4-hour module item | Item property | Key findings | Example quotes |
| Please answer the following questions thinking about each symptom at the time it was at its worst over the past 4 hours. | | | |
| 1. Eye Pain   Please rate the severity of your eye pain at its worst over the past 4 hours:  Participants respond to the item using a 10-point numerical rating scale (NRS), ranging from ‘no eye pain’ (0) to ‘worst possible eye pain’ (10) | **Reading text** | - 9/10 participants reported that they found the item easy to read. - 1/10 participant (F64-OPH-MOD-01-01) was not asked whether the text was easy to read. | **Interviewer: *“And, um, how easy or difficult do you find it to read and answer that question?”*** *“Extremely easy.”* (M54-SUR-MOD-01-02) |
|  | **Understanding** | - 5/10 participants reported that the item was easy to understand. - 5/10 participants were not asked whether the item was easy to understand. | **Interviewer: *“And is there anything that you would reword to make it easier to understand at all?”*** *“No. The wording is fine.”* (F79-SUR-MIL-01-03) |
|  | **Selecting a response** | - 6/10 participants reported that it was easy to select a response to the item. - 2/10 participants reported that it was not easy to select a response. For one participant, this was because they found it hard to think about the severity scale (F55-NOP-SEV-01-06). The other participant reported it wasn't easy for her to answer this question as they experienced eye irritation from trying to focus during the morning 4-hour period, and that factored into their higher rating in the morning. The participant also reported that they would give a lower rating if they were thinking back over a 24-hour period, as their eyes would have adjusted to the daylight. (F74-SUR-MOD-01-09). - 2/10 participants were not asked about ease of selecting a response. | **Interviewer: “*Okay. Thank you. And, um, how easy or difficult was it to select an answer for that question?”*** *“It was easy.”* (F79-SUR-MIL-01-03)  **Interviewer: “*Great. Thank you. And, um, and how easy or difficult is it to read and answer that question?”*** *“This is another one that, it's just hard to try to think about like, yeah, about the, hmm, severity.”* **Interviewer: *“And is that because of the, the scale that we've got there?”*** *“Yes.”* (F55-NOP-SEV-01-06)  *“It wasn’t that easy to answer that question because, like I said, you rate it, I rate it between a 4 and a 5.” “And it wasn’t that easy because, you know, like I said, irritation come in when you’re trying to focus and, and rubbing your eyes, so that has a lot to do with it.”*  **Interviewer: *“…So you said now between a 4 and a 5, but if it was asking you about the past 24 hours, what would you select?”*** *“If it was asking me about the, ah, 24 hours, and I’m just being alert now, I would change that to about a 3 or 4, somewhere like that, because I’m more alert and my eyes are adjusting to the daylight and the light in the house."* (F74-SUR-MOD-01-09) |
| 1. Eye irritation   Please rate the severity of your eye irritation at its worst over the past 4 hours:  Participants respond to the item using a 10-point numerical rating scale (NRS), ranging from ‘no eye irritation’ (0) to ‘worst possible eye irritation’ (10) | **Reading text** | - 9/10 participants reported that the item text was easy to read. - 1/10 participant (F79-SUR-MIL-01-03) was not asked whether the text was easy to read. | **Interviewer: *“And, um, how easy, um, or difficult do you find it to read and understand that question?”*** *“Um, it, it reads very easily.”* (M56-OPH-MIL-01-07) |
|  | **Understanding** | - 10/10 participants reported that the item was easy to understand. | *“Please answer the following questions thinking about each symptom at the time it was at its worst over the past four hours. Two, eye irritation. Please rate the severity of your eye irritation at its worst over the past four hours. Easy to understand, easy to read, very clear, easy to answer.”* (F54-OPH-MIL-01-08) |
|  | **Selecting a response** | - 8/10 participants reported that it was easy to select a response for this item. - 2/10 participants were not asked about ease of selecting a response. | **Interviewer: *“…And is it easy or difficult to select an answer for that question?”*** *“Uh, I think easy.”* (M56-OPH-MIL-01-07) |
| 1. Burning of the eye   Please rate the severity of any burning feelings in your eye(s) at its worst over the past 4 hours:  Participants respond to the item using a 10-point numerical rating scale (NRS), ranging from ‘no eye burning feeling’ (0) to ‘worst possible eye burning feeling’ (10) | **Reading text** | - 8/10 participants reported that it was easy to read this item. - 2/10 participants were not asked whether the text was easy to read this item. | *“Uh, it's easy to read and, uh, easy to follow.”* (M56-OPH-MIL-01-07) |
|  | **Understanding** | - 9/10 reported that the item was easy to understand. - 1/10 participant (F79-SUR-MIL-01-03) was not asked whether the item was easy to understand. | **Interviewer: *“And is that question, um, easy to read and understand?”*** *“It is, yes.”* (M33-OPH-MIL-01-04) |
|  | **Selecting a response** | - 8/10 participants reported that it was easy to select a response for this item. - 2/10 participants were not asked about ease of selecting a response. | **Interviewer: “*Great. And how easy is it to, um, understand and select an answer for that question?”*** *“It's very easy.”* (F49- NOP-SEV-01-05) |
| 1. Eye tiredness   Please rate the severity of your eye tiredness at its worst over the past 4 hours:  Participants respond to the item using a 10-point numerical rating scale (NRS), ranging from ‘no eye tiredness’ (0) to ‘worst possible eye tiredness’ (10) | **Reading text** | - 8/10 participants reported that they found the item text easy to read. - 2/10 participants were not asked whether the text was easy to read. | **Interviewer: “*Great. And is it easy to read and understand that question?”*** *“Yes.”* (F55-NOP-SEV-01-06) |
|  | **Understanding** | - 7/10 participants reported that they found the item text easy to understand. - 1/10 participants (F79-SUR-MIL-01-03) reported that they did not find the item text easy to understand as they were unsure what was meant by the term ‘eye tiredness’. - 2/10 participants were not asked whether the text was easy to read or understand. | **Interviewer: *“And do you find that question, um, easy or difficult to read and understand?”*** *“Yes. Easy to read and understand.”* (M33-OPH-MIL-01-04)  *“Eye tiredness. Uh, I think I always chose zero because I wasn’t sure how to answer that question. Maybe it should have, uh—could have had an option for not applicable*… *Because, uh, eye tiredness. I'm not sure what that means.”* (F79-SUR-MIL-01-03) |
|  | **Selecting a response** | - 5/10 participants reported it was easy to select a response for this item. - 1/10 participants (F79-SUR-MIL-01-03) reported that it was not easy for them to select a response because they did not understand the term ‘eye tiredness’ and so were unsure how to respond to the question. - 2/10 participants were not asked about ease of selecting a response. | **Interviewer: *“And how about to select an answer for that question?”*** *“Um, very easy.”* (F49- NOP-SEV-01-05)  *“I chose zero because, uh, I didn’t feel that my eyes were tired. But, uh, you know, I—but again, the, uh—you know, what that meant, I wasn’t 100% clear.”* (F79-SUR-MIL-01-03) |
| 1. Eye dryness   Please rate the severity of your eye dryness at its worst over the past 4 hours:  Participants respond to the item using a 10-point numerical rating scale (NRS), ranging from ‘no eye dryness’ (0) to ‘worst possible eye dryness’ (10) | **Reading text** | - 10/10 participants reported that they found the item easy to understand. | *“So for eye dryness, um, it's been easy to answer, easy to read, easy to navigate to.”* (M33-OPH-MIL-01-04) |
|  | **Understanding** | - 10/10 participants reported that they found the item easy to read. | *“Number 5, eye dryness. About a three in the morning, about a seven in the afternoon. Easy to, you know, understand and easy to use.”* (M54-SUR-MOD-01-02) |
|  | **Selecting a response** | - 10/10 participants reported that they found selecting a response to be easy. | **Interviewer: *“And how about to select an answer?”*** *“It was very easy.”* (F49- NOP-SEV-01-05) |
| 1. Feeling like there is something in your eye   Please rate the severity of a feeling that you have something in your eye at its worst over the past 4 hours:  Participants respond to the item using a 10-point numerical rating scale (NRS), ranging from ‘no feeling that there is something in my eye’ (0) to ‘worst possible feeling that there is something in my eye’ (10) | **Reading text** | - 10/10 participants reported that it was easy to read the text. | *“Number 6, feeling like there is something in your eye, um, this one varied for me because I fairly rarely had something in my eye, so generally it was in the zero to two range both times of the day. And it was always easy to understand and easy to use.”* (M54-SUR-MOD-01-02) |
|  | **Understanding** | - 9/10 participants reported that they found the item easy to understand. - 1/10 participant (M56-OPH-MIL-01-07) was not asked whether the text was easy to understand. | **Interviewer: “*Okay. How easy, was it easy to read and understand this question?”*** *“Yes.”* (F74-SUR-MOD-01-09) |
|  | **Selecting a response** | - 10/10 participants reported that it was easy to select a response. | “*Okay. Feeling like there is something in your eye. Please rate the severity of a feeling that you have something in your eye at its worst over the past four hours. And no, it's not difficult to decide to answer the question. Again, I do like that it starts with no feeling or worst possible* *feeling, so that it's a little bit easier to rate and it's easy to answer and easy to read.* (F64-OPH-MOD-01-01) |
| 1. Eye itch   Please rate the severity of your eye itch at its worst over the past 4 hours:  Participants respond to the item using a 10-point numerical rating scale (NRS), ranging from ‘no eye itch’ (0) to ‘worst possible eye itch’ (10) | **Reading text** | - 10/10 participants reported that the text was easy to read. | **Interviewer: “*Okay. How easy, was it easy to read and understand this question?”*** *“Yes.”* (F55-NOP-SEV-01-06) |
|  | **Understanding** | - 9/10 participants reported that the item was easy to understand. - 1/10 participant (F49- NOP-SEV-01-05) was not asked whether the item was easy to understand. | “*Eye itch. Please rate the severity of your eye itch at its worst over the past four hours. Very easy to read the question, very easy to select a response.”* (F54-OPH-MIL-01-08) |
|  | **Selecting a response** | - 10/10 participants reported that it was easy to select a response. | *“Eye, eye itch. Please rate the severity of your eye itch at its worst over the past four hours. And again, I like how it says no eye itch or worst possible eye itch. Easy to select, um, the severity and easy to read.”* (F64-OPH-MOD-01-01) |

### COP-Q Symptom Module (24-hour recall period)

All but one participant (n=9/10) reported that their answer would change when thinking back over the past 24-hours rather than the past 4-hours when responding to item one of the Symptom Module (eye pain). Specifically, four participants (n=4/10) reported that their rating on the response scale would be higher if they had to think back on the past 24-hours compared to the past 4-hours as their eyes would be more tired by the end of the day compared to the start of the day.

**Interviewer: *“And if the question asked about the past 24 hours, would your answer change for that question?”*** *“Absolutely. Because when I would answer this during the day, my eye pain would be, you know, maybe six, seven, eight. Where in the morning, it might just be a two, three, or four. So after I've used my eyes, you know, sat in front of a computer for, you know, 10-12, you know, hours or so, there's definitely a, a, a significant increase.”* (M54-SUR-MOD-01-02)

For two participants (n=2/10), the rating on the response scale would be lower when responding thinking about the past 24-hours compared to the past 4-hours as their eye symptoms tend to get better as the day progresses.

*“Um, personally—well personally for me, my eye pain is worse in the mornings and the eye irritation is worse in the mornings. So, um, you know, the, the, the pain and the irritation, um, is different. So 12—four hours, when I do it in the morning, it's going to show a lot more and when I say, you know, the last 24 hours how was it, again my most is in the morning, so as the day goes on, I'm usually a lot better throughout the day, so it's not as severe over 24 as it is over four, if that makes sense.”* (M56-OPH-MIL-01-07)

One participant (n=1/10; F49- NOP-SEV-01-05) reported that their selected response for the 24-hour period recall period would be either lower or higher than when thinking about the past 4-hours, although did not specify what this would depend on, or which would be more likely. For one participant (n=1/10; F55-NOP-SEV-01-06), it was unclear how the rating would change on the 24-hour recall period Symptom Module compared to the 4-hour recall period Symptom Module.

**Interviewer: *“And how would that change?” “****Well within four hours, um, my eyes would be either less dry or dryer or have different symptoms within 24 hours.”* (F49- NOP-SEV-01-05)

**Interviewer: *“And how would that change?”*** *“One reason is like when you're asking for the past 24 hours, okay, so if I'm asleep like eight or six hours, how will I know how much pain I had if I'm sleeping?”* (F55-NOP-SEV-01-06)

Only one participant (n=1/10; F54-OPH-MIL-01-08) reported that they answer would not change depending on the recall period.

### COP-Q Visual Tasking Module (VTM)

The COP-Q Visual Tasking Module (VTM) consists of eight items which assess visual functioning in COSP patients over the ‘past 7 days’. Patients respond to the item using a seven-point verbal descriptor scale, ranging from ‘None of the time’ (0) to ‘All of the time’ (6). Two additional response options ‘I avoided or was completely unable to do this activity due to my eye problems’ (7) and ‘I did not do this for reasons unrelated to my eye problems’ (not applicable) were also included.

Participants were debriefed in the ease of reading the text, understanding the item, selecting a response, appropriateness of the response options, the recall period, and if they would make any changes. Of note, due to time constraints during the interviews, participants were only asked about the font size of the VTM instruction text and about the recall period and response options on the first item in this module, given that these properties are consistent throughout the module.

#### VTM Instruction

Overall, participants found the VTM instruction easy to read, reporting that the font size was large enough (n=9/10 each) and easy to understand (n=8/10). Three participants (n=3/10) suggested some changes regarding the text font size (see section [5.9](#_Recommended_changes)). One participant (n=1/10; F79-SUR-MIL-01-03) was not debriefed on any of the VTM instruction properties and one further participant (n=1/10; F74-SUR-MOD-01-09) was not debriefed on understanding of the VTM instructions (see Table 12 for further detail).

| Table 12. VTM instructions key findings (N=10). | | | |
| --- | --- | --- | --- |
| VTM instruction | Instruction properties | Key findings | Example quotes |
| The following questions ask about how much of the time your eye pain and related problems (e.g., blurriness) affected your ability to do visual activities over the past 7 days. Please do not think about any other vision problems you have (such as difficulties seeing things up close or at a distance) when selecting an answer.  Difficulties doing visual activities might include changing how you did an activity, avoiding an activity, or needing to take a rest from an activity because of your eye pain and related problems. | **Reading text and font size** | - 9/10 participants reported the instruction was easy to read. - 9/10 participants reported that the font size was large enough. - 1/10 participants (F74-SUR-MOD-01-09) reported that the font size could be a slightly larger, however they had no issues reading the text. - 1/10 participant (F79-SUR-MIL-01-03) was not asked about reading the VTM instruction. | **Interviewer: *"Great. Thank you. And, um, and how easy is it to read that instruction?”*** *“Very easy..”* (F49- NOP-SEV-01-05)  *“It was easy, but you know, like I said, sometimes it needs just, just a little bit larger.”* (F74-SUR-MOD-01-09) |
|  | **Understanding** | - 8/10 participants reported that the instruction was easy to understand. - 2/10 participants were not asked whether they understood the VTM instruction. | **Interviewer: *“Okay. Is there anything that you would, uh, maybe change to the text to make it easier to read or understand?”*** *“No. I think it's, uh, very clear.”* (M62-OPH-MIL-01-10) |

#### VTM Items

Almost all participants reported that the VTM items were easy to understand, easy to read and easy to select a response. The font size of the text, recall period and response options were properties that were consistent throughout the module and were debriefed only in the first VTM item due to time constraints (please see Table 13 for further detail).

| Table 13. VTM items key findings (N=10). | | | |
| --- | --- | --- | --- |
| VTM item | Item properties | Key findings | Example quotes |
| Over the past 7 days how much of the time did your eye pain and related problems affect your ability to… | | | |
| 1. Read books, newspapers or magazines for more than one hour?   Participants respond to the item using a 5-point verbal rating scale (VRS), ranging from ‘none of the time’ to ‘all of the time’. Participants were also provided two other response options (‘I avoided or was completely unable to do this activity due to my eye problems’ and ‘I did not do this for reasons unrelated to my eye problems’) | **Reading text and font size** | - 10/10 participants reported that the text was easy to read. - 1/10 (F79-SUR-MIL-01-03) participant reported that the font size was large enough. - 9/10 participants were not asked about the font size. | *“Very easy to read and answer. Like it gives you all the options you could possibly want to choose, so easy…”* (M33-OPH-MIL-01-04)  **Interviewer: *“Great. Thank you. And, um, do you think the font size is large enough?”*** *“Yes..”* (F79-SUR-MIL-01-03) |
|  | **Understanding** | - 10/10 participants reported that the item was easy to understand. | **Interviewer: *“And is there anything you would reword to make it easier to understand at all?”*** *“No.”* (F55-NOP-SEV-01-06) |
|  | **Selecting a response** | - 9/10 participants reported that it was easy to select a response. - It was unclear if 1/10 participant (F74-SUR-MOD-01-09) found it easy or difficult to select a response. | **Interviewer: *“And, um, how easy or difficult was it to select an answer for that question?”*** *“Very easy.…”* (M54-SUR-MOD-01-02) |
|  | **Recall period** | - 5/10 participants reported that it was easy to recall the activity asked about over the past 7 days. - For 3/10 participants, it was not clear if it was easy or not to recall the activity. - 1/10 participant (M54-SUR-MOD-01-02) reported that it was not easy to recall the activity asked about over the past 7 days. - 1/10 participant (F79-SUR-MIL-01-03) was not asked if the activity was easy to recall in the specified time period. | **Interviewer: *“Yeah. And, um, was it easy or difficult to think about this activity over the past seven days?”*** *“Uh, very easy.”* (M56-OPH-MIL-01-07) |
|  | **Response options** | - 8/10 participants reported that the response options were appropriate. - 2/10 participants were not asked whether the response options were appropriate. | *“Yep. Uh, I think it, you know, gave, uh, you know, a nice wide range from all to, to none or it doesn’t apply, so I, I can't think of any, anything else I'd add.”* (M54-SUR-MOD-01-02) |
| 1. Read on a screen for example a computer or tablet for more than one hour?   Participants respond to the item using a 5-point verbal rating scale (VRS), ranging from ‘none of the time’ to ‘all of the time’. Participants were also provided two other response options (‘I avoided or was completely unable to do this activity due to my eye problems’ and ‘I did not do this for reasons unrelated to my eye problems’) | **Reading text** | - 10/10 participants reported that the text was easy to read. | *“Okay. So the next question, read on a screen, for example, a computer or tablet for more than one hour. That was very, uh, easy to read and definitely easy to answer because I do that often.”* (M33-OPH-MIL-01-04) |
|  | **Understanding** | - 10/10 participants reported that the item was easy to understand. | *“Okay. Read on a screen for, for example, a computer or tablet for more than an hour. That's an easy question. It's easy to understand and it's also easy to answer.”* (F55-NOP-SEV-01-06) |
|  | **Selecting a response** | - 10/10 participants reported that it was easy to select a response. | **Interviewer: *“Yeah. Great. And how easy or difficult to select an answer?”*** *“Uh, very easy. It gives you a lot of different answers and a lot of—it, it covers pretty much everything.…”* (M56-OPH-MIL-01-07) |
| 1. Watch a program on the TV for more than one hour?   Participants respond to the item using a 5-point verbal rating scale (VRS), ranging from ‘none of the time’ to ‘all of the time’. Participants were also provided two other response options (‘I avoided or was completely unable to do this activity due to my eye problems’ and ‘I did not do this for reasons unrelated to my eye problems’) | **Reading text** | - 10/10 participants reported that the text was easy to read. | *"Um, so watch a program on the TV for more than one hour. Um, so that was easy to read, easy to answer.”* (M33-OPH-MIL-01-04) |
|  | **Understanding** | - 10/10 participants reported that the item was easy to understand. | *“Watch a program on, on the TV for more than an hour. Okay. That one is an easy one too…It's, um, easy to understand also. Easy to answer.”* (F55-NOP-SEV-01-06) |
|  | **Selecting a response** | - 10/10 participants reported that it was easy to select a response. | *“Okay. Watch a program on TV for more than an hour. I think it reads well and, um, again the questions or the answers, um, give you enough variance that, you know, it gives you plenty of different answers, so you should be able to find one that fits.”* (M56-OPH-MIL-01-07) |
| 1. Watch events at a distance for example a show or sporting event?   Participants respond to the item using a 5-point verbal rating scale (VRS), ranging from ‘none of the time’ to ‘all of the time’. Participants were also provided two other response options (‘I avoided or was completely unable to do this activity due to my eye problems’ and ‘I did not do this for reasons unrelated to my eye problems’) | **Reading text** | - 10/10 participants reported that the text was easy to read. | *“Okay. Watch events at a distance, for example, a show or sporting event. Uh, again it reads very easily...”* (M56-OPH-MIL-01-07) |
|  | **Understanding** | - 10/10 participants reported that the item was easy to understand. | *“Watch events at a distance, for example, a show or sporting event. Um, that one is easy also. It's, it's easy to understand and easy to answer.”* (F55-NOP-SEV-01-06) |
|  | **Selecting response** | - 10/10 participants reported that it was easy to select a response. | *“…watch events at a distance, for example, a show or sporting event. Easy to read, easy to understand, and definitely easy to, to answer.…”* (M33-OPH-MIL-01-04) |
| 1. Drive at night?   Participants respond to the item using a 5-point verbal rating scale (VRS), ranging from ‘none of the time’ to ‘all of the time’. Participants were also provided two other response options (‘I avoided or was completely unable to do this activity due to my eye problems’ and ‘I did not do this for reasons unrelated to my eye problems’) | **Reading text** | - 10/10 participants reported that the text was easy to read. | *“Drive at night. Easy to read...”* (F54-OPH-MIL-01-08) |
|  | **Understanding** | - 10/10 participants reported that the item was easy to understand. | *“Drive at night…It's easy to understand and easy to answer.”* (F55-NOP-SEV-01-06) |
|  | **Selecting a response** | - 10/10 participants reported that it was easy to select a response. | *“Next, driving at night… It was easy to understand…”* (F74-SUR-MOD-01-09) |
| 1. Drive during the day?   Participants respond to the item using a 5-point verbal rating scale (VRS), ranging from ‘none of the time’ to ‘all of the time’. Participants were also provided two other response options (‘I avoided or was completely unable to do this activity due to my eye problems’ and ‘I did not do this for reasons unrelated to my eye problems’) | **Reading text** | - 10/10 participants reported that the text was easy to read. | *“Drive during the day, same feedback. Clear, easy to read...”* (M33-OPH-MIL-01-04) |
|  | **Understanding** | - 10/10 participants reported that the item was easy to understand. | *“Drive during the day. Easy to read, easy to understand, easy to select an answer...”* (F54-OPH-MIL-01-08) |
|  | **Selecting a response** | - 10/10 participants reported that it was easy to select a response. | *“Drive during the day. Okay. That's an easy question to answer…”* (F55-NOP-SEV-01-06) |
| 1. Look in the mirror for example to shave or put your make up on?   Participants respond to the item using a 5-point verbal rating scale (VRS), ranging from ‘none of the time’ to ‘all of the time’. Participants were also provided two other response options (‘I avoided or was completely unable to do this activity due to my eye problems’ and ‘I did not do this for reasons unrelated to my eye problems’) | **Reading text** | - 10/10 participants reported that the text was easy to read. | *“Look in the mirror, for example, to shave or put your makeup on. Um, again it reads very easily and it...”* (M56-OPH-MIL-01-07) |
|  | **Understanding** | - 9/10 participants reported that the item was easy to understand. - 1/10 participants (F74-SUR-MOD-01-09) that the item was not easy to understand as the activity was not relatable to them. | *“Look in the mirror, for example, to shave or put your makeup on. Easy to read, easy to understand…”* (F54-OPH-MIL-01-08)  *“No, it wasn’t, because I was trying to figure out what it really was asking, because I didn't understand clearly exactly what it was asking me about doing, because I don't shave and I don't apply that much makeup on.”* (F74-SUR-MOD-01-09) |
|  | **Selecting a response** | - 9/10 participants reported that it was easy to select a response. - 1/10 participants (F74-SUR-MOD-01-09) reported that it was not easy to select a response as the item was not relatable to them. | *“Look in the mirror, for example, to shave or put your makeup on… easy to answer…”* (M33-OPH-MIL-01-04) |
| 1. Carry out your usual leisure activities or hobbies for example crafts, painting, playing cards?   Participants respond to the item using a 5-point verbal rating scale (VRS), ranging from ‘none of the time’ to ‘all of the time’. Participants were also provided two other response options (‘I avoided or was completely unable to do this activity due to my eye problems’ and ‘I did not do this for reasons unrelated to my eye problems’) | **Reading text** | - 10/10 participants reported that the text was easy to read. | *“Carry out your usual leisure activities or hobbies, for example, crafts, painting, or playing cards. Easy to understand, easy to read, easy to answer as well…”* (M33-OPH-MIL-01-04) |
|  | **Understanding** | - 10/10 participants understood the item. However, one participant (M56-OPH-MIL-01-07) noted that they did not think a lot of people would word the item in this way, although did not provide any further explanation or suggest an alternate way to word this. | *“Over the past seven days, how much of the time did your eye pain and related problems affect your ability to carry out your usual leisure activities or hobbies, for example, crafts, painting, playing cards? Uh-huh. Clear.”* (F79-SUR-MIL-01-03)  *“Carry out your usual leisure activities or hobbies, for example, crafts, painting, playing cards. Um, it reads well, but, um, I don’t know—it, it reminded me of my wife who is [REDACTED] um, when it said leisure activities. Um, I don’t know if, um, a lot of people in [REDACTED] would put it quite that way, so it may be a wording thing, but that's just my opinion. Um, yeah. I, I would think just, um, downtime or, you know, maybe looking at that wording might, might be a thing for some people. Um, and, um, you know, uh, but I think other than that it reads fine.”* (M56-OPH-MIL-01-07) |
|  | **Selecting a response** | - 10/10 participants reported that it was easy to select a response. | *“Over the past seven days, how much of the time did your eye pain and related problems affect your ability to carry out your, um, usual leisure activities or hobbies, for example, crafts, painting, playing cards? Very easy [to answer].”* (F49- NOP-SEV-01-05) |

### COP-Q Health-Related Quality of Life Module (HRQoL)

The COP-Q Health-Related Quality of Life Module (HRQoL Module) consists of five items which assess quality of life in COSP patients over the ‘past 7 days’. These items are responded to on a five-point verbal descriptor scale, ranging from ‘None of the time’ to ‘All of the time’. The final item, which asks about how many nights patients’ eye pain and related problems affected their sleep over the past 7 days, is responded to on a five-point verbal descriptor scale, ranging from ‘0 nights’ to ‘Every night’.

Participants were debriefed on ease of reading the text, font size, understanding of the item, selecting a response, appropriateness of the response options, the recall period and if they would make any changes. Of note, due to time constraints during the interviews, font size and recall period were debriefed only during the HRQoL instruction and item one respectively, given that these properties remained consistent throughout the module.

#### HRQoL Instruction

Overall, all participants demonstrated a good understanding of the HRQoL instruction (N=10/10) and almost half reported that the text was easy to read (n=4/10) (see Table 14 below for further detail).

| Table 14. HRQoL instruction key findings (N=10). | | | |
| --- | --- | --- | --- |
| HRQoL instructions | Instruction properties | Key findings | Example quotes |
| The following questions ask about ways your eye pain and related problems (e.g., blurriness) may have affected you over the past 7 days.  For each question, please choose the answer which describes how much of the time you were affected because of your eye pain and related problems over the past 7 days. | **Reading text and font size** | - 4/10 participants reported the instruction was easy to read. - 6/10 participants were not asked if it was easy to read the instruction. - 2/10 participants reported that the font size was large enough. - 8/10 participants were not asked about the font size. | *“Easy because some words are in bold, which is good...”* (F79-SUR-MIL-01-03)  *“Easy to read, font is perfect. No problem...”* (F54-OPH-MIL-01-08) |
|  | **Understanding** | - 10/10 participants reported that the instruction was easy to understand. | **Interviewer: *“Okay. Great. Thank you. And is there any way that you would change that text to make it easier to read or understand at all?”*** *“No. It's fairly easy to understand…”* (F49- NOP-SEV-01-05) |

#### HRQoL Items

Generally, participants reported that the HRQoL items were easy to understand, easy to read and easy to select a response. The font size of the text and recall period were properties that remained consistent throughout the module and were debriefed only during the HRQoL instruction and item one due to time constraints (please see Table 15 for further detail). Three participants (n=3/10) recommended changes to this module. These can be found in section 4.9.

| Table 15. HRQoL items key findings (N=10). | | | |
| --- | --- | --- | --- |
| HRQoL item | Item properties | Key findings | Example quotes |
| Over the past 7 days… | | | |
| 1. How much of the time did you feel low or depressed?   Participants respond to the item using a 5-point verbal rating scale (VRS), ranging from ‘none of the time’ to ‘all of the time’ | **Reading text and font size** | - 8/10 participants reported that it was easy to read the text. - 2/10 participants were not asked whether the text was easy to read. - 1/10 participants (F79-SUR-MIL-01-03) reported that the font size was large enough. - 9/10 participants were not asked about the font size. | **Interviewer: *"And how easy or difficult is it to read and answer that question?”*** *“It wasn’t difficult at all..”* (F64-OPH-MOD-01-01)  **Interviewer: *“Great. Made a note of that. Brilliant. Thank you. And, um, and do you think that the font size is large enough on that screen?”*** *“Yes.”* (F79-SUR-MIL-01-03) |
|  | **Understanding** | - 9/10 participants reported that the item was easy to understand. - 1/10 participants (F74-SUR-MOD-01-09) reported that it was difficult to understand if the item was referring to feeling low or depressed as related to their eye pain or the item was asking feeling low or depressed more generally. | **Interviewer: *“Okay. Um, and just to confirm… was it easy to understand what the question was asking you?”*** *“Uh, yes, it was.…”* (M62-OPH-MIL-01-10)  **Interviewer: *“Is it easy to understand this question?”*** *“Well, not really, because I was trying to figure out, um, with all of the activities that's been going on in the world, and everything that's going on with you and stuff and, and your household, was it concerning and asking about my health and my eyes or was it just about the other problems that I was going through.”* (F74-SUR-MOD-01-09) |
|  | **Selecting a response** | - 10/10 participants reported that it was easy to select a response. | **Interviewer: *“Um, and how easy or difficult was it to select an answer for that question?”*** *“Very easy.”* (M54-SUR-MOD-01-02) |
|  | **Recall period** | - 1/10 participants (M56-OPH-MIL-01-07) reported that the recall period was easy to think back over. - 1/10 participants (F49- NOP-SEV-01-05) reported that the recall period was difficult to think back over. - 8/10 participants were not asked about the recall period of the module. | **Interviewer: *“Okay. Thank you. And, um, and how—was it easy or difficult to think back over the past seven days for that question?”*** “*Very easy.”* (M56-OPH-MIL-01-07)  *“It was a little more difficult over seven days. That was a little more difficult. But my eyes are pretty consistent when it comes to dryness, so over seven days is usually worse in the mornings and lighter at night. Easier—well they feel better at night.”* (F49- NOP-SEV-01-05) |
|  | **Response options** | - 2/10 participants reported that the response options were appropriate. - 8/10 participants were not asked whether the response options were appropriate. | **Interviewer: “*And, um, and what do you think of the response options?”*** *“Very easy as well.”* (F49- NOP-SEV-01-05) |
| 1. How much of the time did you feel anxious?   Participants respond to the item using a 5-point verbal rating scale (VRS), ranging from ‘none of the time’ to ‘all of the time’ | **Reading text** | - 8/10 participants reported that it was easy to read the text. - 2/10 participants were not asked whether the text was easy to read. | *“Okay. How much of the time did you feel anxious? Easy to read and understand..”* (M33-OPH-MIL-01-04) |
|  | **Understanding** | - 7/10 participants found the item easy to understand. - 3/10 participants were not asked whether the item was easy to understand. | *“How much of the time did you feel anxious? It's easy to understand and, um, easy to answer.”* (F55-NOP-SEV-01-06) |
|  | **Selecting a response** | - 9/10 participants reported that it was easy to select a response. - 1/10 participant (M62-OPH-MIL-01-10) was not asked whether it was easy to select a response to the item. | *“How much of the time did you feel anxious? Um, uh, I think it's a very good worded question, um, and the answers are fine. It, it, you know, gives you everything you need.”* (M56-OPH-MIL-01-07) |
|  | **Response options** | - 2/10 participants reported that the response options were appropriate. - 8/10 participants were not asked about the response options. | *“How much of the time did you feel anxious? None of the time. Easy to read, easy to answer, responses were clear and appropriate.”* (F54-OPH-MIL-01-08) |
| 1. How much of the time did you feel frustrated?   Participants respond to the item using a 5-point verbal rating scale (VRS), ranging from ‘none of the time’ to ‘all of the time’ | **Reading text** | - 7/10 participants reported that it was easy to read the text. - 3/10 participants were not asked about ease of reading the text. | *“Uh, Number 3. How much time did you feel frustrated? Again, very easy to read and answer.”* (M54-SUR-MOD-01-02) |
|  | **Understanding** | - 7/10 participants reported that the item was easy to understand. - 1/10 participant (F64-OPH-MOD-01-01) reported that it was difficult to understand if the item was referring to frustration related to their eye pain or if the item referred to frustration more generally. - 2/10 participants were not asked about ease of understanding the item. | *“How much of the time did you feel frustrated? That one is easy to understand and easy to answer.”* (F55-NOP-SEV-01-06)  *“Um, how much of the time did you feel frustrated? Um, it wasn’t hard to answer, but I did have to think about why, why this had to do what it had to do with my vision.”* (F64-OPH-MOD-01-01) |
|  | **Selecting a response** | - 9/10 participants reported that it was easy to select a response to the item. - 1/10 participant (M62-OPH-MIL-01-10) was not asked whether they found it easy to select a response. | *“How much of the time did you feel frustrated? But, uh, none of the time. And that was easy… easy to answer, and appropriate responses.”* (F54-OPH-MIL-01-08) |
|  | **Response options** | - 3/10 participants reported that the response options were appropriate. - 7/10 participants were not asked whether the response options were appropriate. | *“Um, how much of the time did you feel frustrated? Um, so I think that's well-written. I think it makes people think more. And, um, and again, the questions—the answers are good.”* (M56-OPH-MIL-01-07) |
| 1. How much of the time did you feel worried?   Participants respond to the item using a 5-point verbal rating scale (VRS), ranging from ‘none of the time’ to ‘all of the time’ | **Reading text** | - 8/10 participants reported that it was easy to read the text. - 2/10 participants were not asked about ease of reading the text. | *"How much of the time did you feel worried? Again, very easy to read and answer.”* (F64-OPH-MOD-01-01) |
|  | **Understanding** | - 8/10 participants reported that the item was easy to understand. - 1/10 participant (F64-OPH-MOD-01-01) reported that it was difficult to understand if the item was referring to feeling worried in relation to their eye pain or if the item referred feeling worried more generally. - 1/10 participant (M54-SUR-MOD-01-02) was not asked whether the item was easy to understand. | *“How much of the time did you feel worried? Uh, it's easy to understand also and easy to answer.…”* (F55-NOP-SEV-01-06)  *“Um, how much time do you feel you worried? Um, again, it was easy to answer, but then the question was is, is it worried about the dryness or vision issues or is this just my general well being. I don’t think it really explained that reason for asking that question.”* (F64-OPH-MOD-01-01) |
|  | **Selecting a response** | - 10/10 participants reported that it was easy to select a response to the item. | *“How much of the time did you feel worried? None of the time. Easy to read, easy to answer, appropriate selections for me.”* (F54-OPH-MIL-01-08) |
|  | **Response options** | - 2/10 participants reported that the response options were appropriate. - 8/10 participants were not asked whether the response options were appropriate. | *“How much of the time did you feel worried? None of the time. Easy to read, easy to answer, appropriate selections for me.”* (F54-OPH-MIL-01-08) |
| 1. How many nights did your eye pain and related problems affect your sleep?   Participants respond to the item using a 5-point verbal rating scale (VRS), ranging from ‘0 nights’ to ‘every night’ | **Reading text** | - 8/10 participants reported that it was easy to read the text. - 2/10 participants were not asked about ease of reading the text. | *“How many nights did your eye pain and related problems affect your sleep?...And that's obviously very easy to read.”* (F64-OPH-MOD-01-01) |
|  | **Understanding** | - 8/10 participants reported that it was easy to understand the item. - 2/10 participants were not asked whether it was easy to understand the item. | *“How many nights did your eye pain and related problems affect your sleep? Um, that one is easy to understand. Uh, I guess that one was a little—it's not hard to answer that one. It's, it's easy.”* (F55-NOP-SEV-01-06) |
|  | **Selecting response** | - 10/10 participants reported that it was easy to select a response. | *“How many nights did your eye pain and related problems affect your sleep? Zero nights. Easy to read, easy to answer, easy to recall, appropriate responses.…”* (F54-OPH-MIL-01-08) |
|  | **Response options** | - 2/10 participants reported that the response options were appropriate. - 8/10 participants were not asked whether the response options were appropriate. | *“How many nights did your eye pain and related problems affect your sleep? Zero nights. Easy to read, easy to answer, easy to recall, appropriate responses.”* (F54-OPH-MIL-01-08) |

### COP-Q ‘Patient facing text’

#### ‘Patient facing text’ debriefing findings

Participants were asked (N=10/10) about the ‘patient facing text’ included in the pop-up boxes that explained how to quit, skip, or look for help during the completion of the questionnaires (Appendix A). All participants were asked about the location of the buttons, the understanding of the instructions and text, ease of reading the text and font size, and if they had ever needed to use any of them during the four-week data collection period. The key findings for the quit, skip, and help buttons can be found in Table 16. Six participants (n=6/10) recommended changing some aspects of these buttons. These recommendations can be found in section 4.9.

| Table 16. ‘Patient facing text’ of the pop-up boxes key findings (N=10). | | | |
| --- | --- | --- | --- |
| Pop-up box | Key findings |  | Example quotes |
| Quit button | | | |
| Location | - 10/10 participants reported that the quit button was easy to locate^[[1]](#footnote-2)^. | **Interviewer:** *“****Perfect. Okay. Now if I could please ask you is it clear of what to do if you want to quit this questionnaire?” “****Yes. There is a quit button.”* (F54-OPH-MIL-01-08) | |
| Understanding | - 10/10 participants demonstrated good understanding of the quit button instructions. | *“Uh, basically it's saying if you want to, uh, quit, uh, you can confirm that you want to quit and then all your answers will be saved.”* (M62-OPH-MIL-01-10) | |
| Use during data collection period | - 10/10 participants reported that they did not have to use the quit button during the four-week data collection period. | *“I have not used the quit button.”* (F54-OPH-MIL-01-08) | |
| Reading text and font size | - 4/10 participants reported that the font size of the text in the quit box was large enough. - 4/10 participants reported that the font size could be a bit larger; however, it is worth noting that these participants had no issues with reading the text. - 1/10 participant (M56-OPH-MIL-01-07) reported that it wasn’t clear if the font size was large enough as they could not remember; however, they had no issues reading the text. - 1/10 participant (M33-OPH-MIL-01-04) was not asked about ease of reading the text or the font size. | *“Uh, I would say easy. Um, you know, instead of extremely easy, might make the print just a little bigger. But I mean I didn’t have any trouble reading it, but that's the only thought that I could say about that.”* (M54-SUR-MOD-01-02)  **Interviewer: *“Great. Thank you. And is the font size large enough in that box?”*** *“Um, I'm sorry, I honestly don’t remember. I went through it so quickly.”* (M56-OPH-MIL-01-07) | |
| Skip button | | | |
| Location | - 6/10 participants reported that the skip button was not easy to locate. - 3/10 participants reported that the skip button was easy to locate. - It was unclear if 1/10 participant (F79-SUR-MIL-01-03) found the skip button easy to locate. | *"Um, no. It's not clear what you would need to do. Um, a few clicking around would probably easily get someone there, but there's no, you know, dialogue that says click next to skip, which could be added to make that clear*.” (M33-OPH-MIL-01-04) | |
| Understanding | - 10/10 participants demonstrated a good understanding of the skip button instructions. | *“That now notifies us that we are able to skip this question by clicking confirm or cancel to go back and answer.”* (M33-OPH-MIL-01-04) | |
| Use during data collection period | - 10/10 participants reported that they did not have to use the skip button during the four-week data collection period. | **Interviewer: *“Okay. Great. And have you ever needed to use this button at all during the past four-weeks?”*** “*No, I have not.”* (M62-OPH-MIL-01-10) | |
| Reading text and font size | - 3/10 participants reported that the font size of the text in the skip box was large enough and that they had no issues reading the text. - 3/10 reported that the font size could be a bit larger; however, these participants had no issues reading the text. - 4/10 participants were not asked about the font size; however, they did not have any issues reading the text inside the skip button. | *“Uh, very easy. It could be larger. It's just that since it's in that box.”* (M33-OPH-MIL-01-04) | |
| Help button | | | |
| Location | - 7/10 participants reported that the help button was not easy to locate however over half of these participants (n=4/7) noted that they were not looking for this button and which may be why they could not locate it. - 3/10 participants reported that the help button was easy to locate. | *“Um, it's a little difficult to find, but again I haven't looked for it, so I guess that's one of the reasons.”* (M56-OPH-MIL-01-07) | |
| Understanding | - 10/10 participants demonstrated a good understanding of the help button instructions. | *“It’s telling me what seems pretty obvious. Previous is previous, next is next. The only thing I learned was that I can skip and go to next, but you taught me that on the last screen, so it says what it says and I don’t know why anyone wouldn’t understand it.”* (F49- NOP-SEV-01-05) | |
| Use during data collection period | - 10/10 participants reported that they did not have to use the help button during the four-week data collection period. | **Interviewer: *“And have you needed to use that button at all during the past four-weeks?”*** “*I have not.”* (M33-OPH-MIL-01-04) | |
| Reading text and font size | - 1/10 participant (F54-OPH-MIL-01-08) reported that the font size of the text in the help box was large enough and they had no issues reading it. - 1/10 participant (F74-SUR-MOD-01-09) reported that the font size should be a bit larger; however, this participant had no issues reading the text. - 8/10 participants were not asked about the font size of the help button; however, the size of the text inside the box was the same as the quit and skip button and these participants reported no issues with reading the text in these pop-up boxes. | **Interviewer: *“Okay, great, thank you so much, and was it easy to read this information?”*** *“Yes, but I feel like the print needs to be just a little larger.”* (F74-SUR-MOD-01-09) | |

### Relevance of the COP-Q

When asked whether the COP-Q questions captured their experience of COSP over a four-week period, all participants asked reported that the questions were relevant to their experience of COSP (n=7/10). Due to time constraints, three participants were not asked this question (n=3/10).

**Interviewer: *“…do you think that they capture your experience of your chronic eye pain well, all these questions?”*** *“Yeah, I think it, it helps me to be more alert what was going on with my eyes… I think everything that it asked me was fine, because like I said, it made me more observant or more alert what was going on and everything, and what would cause some of the problems that I were having and everything, if I were doing too much of this and if I were doing too much of that.”* (F74-SUR-MOD-01-09)

**Interviewer: *“Across all of these questionnaires, do you feel that they capture your experience of chronic eye pain during the four-week period?”*** *“Yes.”* (F79-SUR-MIL-01-03)

## Debriefing of the PGI items

### PGI-S Items

Several item properties were debriefed for the PGI-S items (severity of eye pain, severity of eye pain and related problems and severity of limitations in carrying out visual activities). These included ease of reading the text, understanding of the item, selecting a response, appropriateness of the response options, the recall period, and recommended changes. Due to time constraints, not all participants were debriefed for each item and item property. No participants recommended changes to these items.

The majority of the participants found it easy to recall severity of their eye pain and related symptoms over a 7-day recall period. However, one participant (n=1/10; F49- NOP-SEV-01-05) reported that it was challenging to recall symptoms like itchiness and blurriness over a 7-day period.

*“Um, it was a little more challenging to remember. If, if you have eye pain, that's a little easier to remember. Remembering each day if you—if your eyes were itching or blurry or irritated, irritated maybe, but, um, itchy or blurriness is a little more challenging.”* (F49- NOP-SEV-01-05)

The key findings are summarized below in Table 17.

| Table 17. PGI-S items key findings (N=10). | | | |
| --- | --- | --- | --- |
| PGI-S item | Item properties | Key findings | Example quotes |
| 1. Severity of eye pain   Please choose the response that best describes the overall severity of your eye pain over the past 7 days (select one response).  Participants respond to the item using a 4-point verbal rating scale (VRS), ranging from ‘none’ to ‘severe’ | **Reading text** | - 7/10 participants reported that it was easy to read the text. - 3/10 participants were not asked about ease of reading text. | *“Please choose the response below that best describes the overall severity of your eye pain over the past seven days and select one response. Moderate. It's easy to understand. It was easy to answer.”* (F64-OPH-MOD-01-01) |
|  | **Understanding** | - 10/10 participants demonstrated that they understood the question as intended. | *“Please choose the response below that best describes the overall severity of your eye pain over the past seven days. Select one response. I would say mild. Easy to read, easy to select, easy to recall.”* (F54-OPH-MIL-01-08)  **Interviewer: *“…And what were you thinking about when answering that question?”*** *“How, how much my eye pain has, you know, bothered me over the last week. I mean, uh, I guess I should say the, the level of the eye pain, i.e., the severity. How, how high it's been, which to me also means how much it bothers me.”* (M54-SUR-MOD-01-02) |
|  | **Selecting a response** | - 9/10 participants reported that it was easy to select a response. - 1/10 participant (M56-OPH-MIL-01-07) was not asked about ease of selecting a response. | **Interviewer: *“And how easy or difficult is it to select an answer for that question?”*** “*Very easy. It's easier than the last set of questions because this kind of gives you a, um, a broader answer. You know, mild, moderate, severe. You know, and that is something I can say, okay, over the past seven days my eyes were pretty moderate with pain as opposed to, you know, breaking it down a little more. It was easier.”* (F49- NOP-SEV-01-05) |
|  | **Response options** | - 6/10 participants reported that they found the response options appropriate. - 4/6 participants demonstrated that they understood the difference between the response options. - 2/6 participants were not asked whether they understood the difference between the response options. - 4/10 participants were not asked whether the response options were appropriate. | *“I mean I feel like those are—that's sufficient answers, answer choices for a seven-day time period. It's just kind of like an average, so those are good choices.”* (M33-OPH-MIL-01-04)  **Interviewer: *“...Could you please, uh, tell me—could you please describe the difference between, uh, mild and none?”*** *“None would be I didn’t experience anything at all. Mild is that I experienced it slightly. Like maybe once a day, maybe, you know, a couple times, but not ridiculous.”* (F54-OPH-MIL-01-08) |
| 1. Severity of eye pain and related problems   Please choose the response below that best describes the overall severity of your eye pain and related problems (e.g., itch, irritation, blurriness) over the past 7 days (select one response).  Participants respond to the item using a 4-point verbal rating scale (VRS), ranging from ‘none’ to ‘severe’ | **Reading text** | - 5/10 participants reported that it was easy to read the item text. - 5/10 participants were not asked about ease of reading the item. | **Interviewer: *“… And, um, and how easy is it to read that question?”*** *’It's okay.’It's fine.”* (F79-SUR-MIL-01-03) |
|  | **Understanding** | - 8/10 participants demonstrated that they understood the item as intended. - 2/10 participants were not asked about understanding of the item. | **Interviewer: *“Okay. And what were you thinking about when answering that question?”*** *“I was just thinking about, um, you know, over the past seven days if I had any itch or irritation or blurriness.”* (F49- NOP-SEV-01-05)  *“That was, uh, very easy to understand and read and answer.”* (M33-OPH-MIL-01-04) |
|  | **Selecting a response** | - 8/10 participants reported that it was easy to select a response option. - 2/10 participants were not asked about ease of selecting a response. | *“Okay. So, um, let's see. Number 2 on eye pain and related problems, um, yeah. Again the severe, moderate, mild, none. Um, yep. I mean very, very easy to understand. Very easy to answer.”* (M54-SUR-MOD-01-02) |
|  | **Response options** | - 2/10 participants demonstrated understanding of the response options. - 8/10 participants were not asked about the appropriateness of the response options. | **Interviewer: *“Okay. And could you please describe the difference between let's say mild and moderate?”*** *“Moderate would mean to me closer to half the time. Mild is just occasionally… Like I experienced it in a noticeable way three or four days out of seven would make it more moderate.”* (F54-OPH-MIL-01-08) |
| 1. Severity of limitations in carrying out visual activities   Please choose the response below that best describes the overall severity of your limitations in carrying out visual activities (e.g., reading, watching the TV) over the past 7 days (select one response).  Participants respond to the item using a 4-point verbal rating scale (VRS), ranging from ‘none’ to ‘severe’ | **Reading text** | - 5/10 participants reported that it was easy for them read the item text. - 5/10 participants were not asked about ease of reading the item. | *“That's also easy to read and think back on and answer as well… thinking about like some of the most common activities, which like eye irritation really impacts me and how often that happens.”* (M33-OPH-MIL-01-04) |
|  | **Understanding** | - 8/10 participants demonstrated that they understood the question as intended - 2/10 participants were not asked | “*I would again select none for this…* *I was thinking about whether my eye irritation over the past seven days caused me to not want to do these things or to make them difficult and that did not occur.”* (F54-OPH-MIL-01-08)  “*That was—Number 3, limitations on carrying out visual activities. Severe, moderate, mild, none. Again, very simple to understand. Easy to answer that question.”* (M54-SUR-MOD-01-02) |
|  | **Selecting a response** | - 8/10 participants reported that it was easy to select a response for this item. - 2/10 participants were not asked about ease of selecting a response. | **Interviewer: *“Okay. And, um, was it easy or difficult to select your answer?”*** *“Very easy.”* (F54-OPH-MIL-01-08) |
|  | **Response options** | - 1/10 participant (F74-SUR-MOD-01-09) demonstrated understanding of the response options. - 9/10 participants were not asked about the appropriateness of the response options. | “*I would select 7, I mean I would select mild, if I’m going to watch television like, and read.”* **Interviewer: *“Yeah, and what were you thinking when you were answering this question?”*** *“I have no idea. Um, well, I probably was thinking about at that time, I didn't have any irritation, nor blurriness, or eye itching at that time when I selected that answer.”* (F74-SUR-MOD-01-09) |

### PGI-C Items

Several item properties were debriefed for the PGI-C items (change in eye pain, change in eye pain and related problems and change in limitations in carrying out visual activities). These included ease of reading the text, understanding of the item, selecting a response, appropriateness of the response options, and the recall period (please see Table 18 for further detail). No participants recommended making any changes to the PGI-C items. Due to time constraints, not all participants were debriefed for each item and item property.

Five participants (n= 5/10) reported that it was easy to recall changes in their eye pain over a four-week period. Five participants were not asked (n=5/10).

**Interviewer: *“And, um, was it easy or difficult to think back over the past four-weeks for that question?”*** “*Um, it was, it was easy because had it been a marked change one way or the other, I would definitely have remembered.”* (M54-SUR-MOD-01-02)

| Table 18. PGI-C items key findings (N=10). | | | |
| --- | --- | --- | --- |
| Item | Item properties | Key findings | Example quotes |
| 1. **Change in eye pain**   **Please choose the response that best describes the overall change in your eye pain compared to when you started this study (select one response)**  Participants respond to the item using a 5-point verbal rating scale (VRS), ranging from ‘much better’ to ‘much worse’ | **Reading text** | - 7/10 participants reported that it was easy to read the item text. - 3/10 participants were not asked about ease of reading the item. | *“So please choose the response below that best describes the overall change in your eye pain compared to when you started this study. Easy to read and understand.”* (M33-OPH-MIL-01-04) |
|  | **Understanding** | - 9/10 participants demonstrated that they understood the item as intended. - 1/10 participant (M56-OPH-MIL-01-07) was not asked about understanding of the item. | “*No change would be my answer. It was easy to read, easy to understand, and nothing has changed about my eyes during this study.”* (F54-OPH-MIL-01-08)  **Interviewer: *“And what, and what were you thinking about when answering that question?”*** *“Um, thinking about like when I first was given a tablet compared to where I am now, has, has there been any difference in my eyes.”* (F49- NOP-SEV-01-05) |
|  | **Selecting a response** | - 8/10 participants reported that it was easy to select a response for this item. - 2/10 participants were not asked about ease of selecting a response. | **Interviewer: *“And, um, how easy or difficult was it to select an answer for that*** ***question?”*** *“Very easy.”* (F49- NOP-SEV-01-05) |
|  | **Response options** | - 4/10 participants reported that the response options were easy to understand and demonstrated an understanding of the difference between them. - 6/10 participants were not asked about the response options. | **Interviewer: *“…what does, um, let's say much better mean to you, if you could describe that?”*** “*That since I started this study, my eyes are less itchy and irritated*.” **Interviewer: *“And what would much worse mean to you?”*** “*That my eyes are more itchy and irritated than they were before I started the study.”* (F54-OPH-MIL-01-08) |
| 1. **Overall change in eye pain and related problems**   **Please choose the response below that best describes the overall change in your eye pain and related problems (e.g., itch, irritation, blurriness) compared to when you started this study (select one response)**  Participants respond to the item using a 5-point verbal rating scale (VRS), ranging from ‘much better’ to ‘much worse’ | **Reading text** | - 4/10 participants reported that it was easy to read the item text. - 6/10 participants were not asked about ease of reading the text. | *“Um, easy to read and understand. Um, what I think about, you know, I was just thinking about from when I first started, you know, any—versus now that has developed or has it maintained.”* (M33-OPH-MIL-01-04) |
|  | **Understanding** | - 5/10 participants demonstrated that they understood the question as intended. - 5/10 participants were not asked about understanding of the item. | *“Mild is more of a, you know, just a, um, a realization that there is a, uh, you know, something is not quite normal or quite right with your eye, your eyes, whereas moderate is a definite, uh, a definite feeling. It's not just a matter of it being a more—a little bit of an irritation or an inconvenience, but it's actually something that's starting to cause you, uh, problems with, uh, performance and, and being able to see and do things.”* (M62-OPH-MIL-01-10)  **Interviewer: *“And what were you thinking about when answering that question?”*** “*I was thinking about, um, when I started this study, if I had, you know, any itch or irritation or blurriness.”* (F49- NOP-SEV-01-05) |
|  | **Selecting a response** | - 5/10 participants reported that they found it easy to select a response for this item. - 5/10 participants were not asked about selecting a response option. | **Interviewer: *“…And could you tell me what were you thinking about when you were answering this question?*** *“Um, again with the overall, uh, feeling of my eyes, uh, dryness, irritation, itchiness. Uh, and how they generally felt, uh, during that period.”* **Interviewer: *“And, um, was it easy for you to, to select an answer?”*** *“Yes, it was.”* (M62-OPH-MIL-01-10) |
|  | **Response options** | - 1/10 participant (F54-OPH-MIL-01-08) demonstrated that the response options were easy to understand and could differentiate between the response options - 9/10 participants were not asked | **Interviewer: *“And could you please describe the difference between let's say, uh, no change and a little better, the difference between these two options?”*** *“A little better would mean I could notice a slight change in my itch and irritation today than previously before I started the study.”* (F54-OPH-MIL-01-08) |
| 1. **Overall change in limitations in carrying out visual activities**   **Please choose the response below that best describes the overall change in your limitations in carrying out visual activities (e.g., reading, watching the TV) compared to when you** **started this study (select one response)**  Participants respond to the item using a 5-point verbal rating scale (VRS), ranging from ‘much better’ to ‘much worse’ | **Reading the text** | - 3/10 participants reported that it was easy to read the item text. - 7/10 participants were not asked | *“Easy to read, easy to think back on, thinking about, you know, uh, the change over the past, you know, month or so if one has occurred, and it's easy to answer.”* (M33-OPH-MIL-01-04) |
|  | **Understanding** | - 5/10 participants demonstrated that they understood the item as intended. - 5/10 participants were not asked about whether they understood the item. | *Again my answer would be no change… If I read less, watched less TV, and if I did, was it a result of any eye issues and was it better or worse this current month than it was previous to starting the study. The answer was no.”* (F54-OPH-MIL-01-08)  *“Easy to read, easy to think back on, thinking about, you know, uh, the change over the past, you know, month or so if one has occurred, and it's easy to answer.”* (M33-OPH-MIL-01-04) |
|  | **Selecting a response** | - 5/10 participants reported that it was easy to select a response for this item. - 5/10 participants were not asked about ease of selecting a response. | **Interviewer: *“And what were you thinking about when answering that question?”*** *“If there was any change in my, um, vision or eyes, um, compared to when I started the study as opposed to now.”*  **Interviewer: *“And how easy or difficult was it to select an answer for that question?”*** *“Very easy.”* (F49- NOP-SEV-01-05). |
|  | **Response options** | - 1/10 participant (F54-OPH-MIL-01-08) demonstrated that it was easy to understand the response options and differentiate between them. - 9/10 participants were not asked about the response options. | **Interviewer: *“And could you please just describe the difference between no change and a little worse?”*** *“A little worse would mean that I didn’t read and watch TV quite as much over the past month.”* (F54-OPH-MIL-01-08) |

## Recommended changes

A total of seven (n=7/10) participants recommended changes to be made to: the tablet device (n=3/7), ePRO interface (n=3/7), and items of the COP-Q (N=7). Three participants did not recommend any changes to be made (n=3/10). Most participants recommended changes as personal preferences and few participants reported an impact on their ability to use the tablet and/or respond to the ePRO (see Table 19 for further detail)

| Table 19. Recommended changes (N=10). | | | |
| --- | --- | --- | --- |
| Theme | Recommended changes | Key findings | Example quotes |
| Changes to the tablet device | **Protective case** | - 1/10 participant (F64-OPH-MOD-01-01) recommended that a protective case was provided with the tablet device so that it could be stored safely. | *“Um, no. Maybe put a case in the, um, box, some kind of padded case to keep it a little safer. Not that I dropped the tablet or anything, but I usually just kept it in my purse and my bag.”* (F64-OPH-MOD-01-01) |
|  | **Tablet stand** | - 1/10 participant (M33-OPH-MIL-01-04) mentioned that the tablet device was heavy, and recommended that participants are provided with a stand to alleviate any issues with holding the device when completing the COP-Q. | *“Um, the tablet itself, um, maybe it's a little heavy. Basically, I'm not older, but maybe for someone older who has like osteoporosis or something like that or arthritis, it might pose an issue like trying to hold it. So maybe if it had like some type of stand, that could help.”* (M33-OPH-MIL-01-04) |
|  | **Tablet size** | - 1/10 participant (F55-NOP-SEV-01-06) recommended the tablet device to be smaller in size, so it is easier for future participants to carry around if needed. | *“Also if, also if it was like a smaller—not, not the big tablet, like a small tablet like the size of a, um, a cell phone, then it would have been easier because I could carry it with me.”* (F55-NOP-SEV-01-06) |
| Changes related to timing of completing the ePRO | **Time windows** | - 3/10 participants recommended changes to be made to the time windows in which the COP-Q was to be completed. - 2/3 participants noted that the morning and evening time windows should be of the same duration. - 1/3 participants (F55-NOP-SEV-01-06) recommended that the evening time window should be slightly longer so that it allows for more flexibility. | *“…So maybe the time should be the same. Four hours for the morning, four hours for the evening.”* (F64-OPH-MOD-01-01)  *“Um, I think the ending hours should be like consistent. So like instead of it ending at 10:00 a.m. and 11:00 p.m., it should both end at 10:00 a.m.—10:00 p.m. or 11:00 a.m. and 11:00 p.m. just so that eliminates a little bit of confusion.”* (M33-OPH-MIL-01-04)  *“Like maybe the morning one wasn’t too bad. The, the afternoon—the one for night, that was the one that was a little hard because I would have to rush to get home. Um, maybe it should last until 12:00 at night. Like maybe from 7:00 to 12:00 or 6:00 to 12:00. And maybe in the morning from 6:00 to 12:00.”* (F55-NOP-SEV-01-06) |
|  | **Alarm/reminders** | - 2/10 participants recommended making changes to the alarms/reminders. - 1/2 participant (F64-OPH-MOD-01-01) recommended that it would be helpful to have a second alarm following the first one, in case future participants are unable to complete the ePRO after the first alarm goes off. - 1/2 participant (M54-SUR-MOD-01-02) noted that the alarm was unreliable and did not always ring at the time it was set for. Though they did not find it to be a major issue, they recommended fixing it as it is an important function for anyone who wants to closely track their timings. | *“I would recommend that if the time is running out that maybe you have a second alarm to remind me if I'm busy or haven't answered the questionnaire to remind me that I need to do that. I guess, if that makes sense.”* (F64-OPH-MOD-01-01)  **Interviewer: *“… is there anything that you would change about the tablet device at all?”*** *“Um, I, I did notice that it seemed like when you set the alarm that the alarm didn’t always go off at the time you set it for…And that seemed to happen a couple of times. That's not really a major, you know, issue, but if somebody was really trying to track and they wanted to do it very close to the correct time, you know, each day or to be reminded to it at all then…that might be important to note.”* (M54-SUR-MOD-01-02) |
| Changes to visual aspects of the ePRO | **Layout** | - 3/10 participants recommended making changes to the visual aspects of the ePRO. - 1/3 participant (M54-SUR-MOD-01-02) recommended that there should be less white space between the questions and the response options; however they noted that this was a personal preference. - 1/3 participant (M33-OPH-MIL-01-04) recommended that the text referring to the recall period in each question (i.e., days and hours) could be made bold so that it is clearer for participants to read. - 1/3 participant (F64-OPH-MOD-01-01) recommended that an extra screen be added at the end of the four-week data collection period indicating that there are no other questionnaires to be completed, as this would be very helpful for future participants. | *“Uh, it seemed like there was a lot of white space between the question at the very top and the scale, which was in the lower half. I would have personally preferred the question and the Likert scale boxes to be closer to each other. That's just a personal preference.”* (M54-SUR-MOD-01-02)  *“Uh, very straightforward. Maybe perhaps like the font for the times and the date a little larger. Other than that, it's very clean and easy to read.”* (M33-OPH-MIL-01-04)  **Interviewer: *“So do you think it would be useful if, if perhaps, um, it said something like that on the screen maybe?”*** *“Yes. To say you have no further questionnaires to complete. This is the end of your study or of this phase of your study.”* (F64-OPH-MOD-01-01) |
|  | **Colour of text** | - 1/10 participant (M33-OPH-MIL-01-04) noted that the colour of the question text should be different to the colour of the response options so that participants can spot them easily when responding to the items. However, this participant reported no issues answering the questions. | *“Again, I would just make the question, um, a different colour… It's just something about it. The question itself doesn’t stand out to me that much. Like I can clearly see it, but I can see someone just simply looking at the answers and they're like, what am I answering.”* (M33-OPH-MIL-01-04) |
|  | **Background colours** | - 1/10 participant (M33-OPH-MIL-01-04) noted that a different colour scheme rather than black and white might have been a better choice for the background colours; however they did not provide any alternative colour scheme recommendations. This participant did not experience any issues answering the questions. | *“Um, it's very easy to answer the question. Um, it's pretty easy to read it as well. Um, maybe make it a little larger. I don’t know. I'm thinking a little larger. It's just something about—or a different colour scheme actually now that I'm looking at it. It's just something about that black and white that's, I don’t know.”* (M33-OPH-MIL-01-04 |
| Changes to the accessibility of the ePRO | **Getting to the next screen** | - 2/10 participants recommended changes for how to navigate to the next screen of the ePRO. - 1/2 participant (M33-OPH-MIL-01-04) recommended that the ‘Next’ and ‘Previous’ buttons on the screen could be made a different colour from the background to stand out. This participant also suggested that the font size of COP-Q title page could be larger, however they did not have an issue reading the text or locating the buttons. - 1/2 participant (M54-SUR-MOD-01-02) recommended that the ‘Next’ button could be higher up on the tablet screen. | **Interviewer: *“Okay. Great. Thank you. And is it clear how you would get to the next screen?”*** *“It is. It is. I would probably, um, like make some type of border around the previous and the next. Like you know how it's just like that grey bar that's like really the same colour as the background and just the text was next? Maybe if we like put like a blue box or* *like some type of box around the next and the previous to make it stand out.” (M33-OPH-MIL-01-04)*  *“And now that we’re talking about next, um, you know, to me, I would put the next higher up on the screen…But that’s just a personal thing. It’s not any flaw in the device or the program.”* (M54-SUR-MOD-01-02)  **Interviewer: *“And how easy or difficult is it to read that text?”*** *“It's, um, it's very easy but, I don’t know, maybe make it a little larger.”* (M33-OPH-MIL-01-04) |
| Changes to the COP-Q items | **COP-Q Eye Pain Severity Module** | - 4/10 participants recommended changes on various properties of the item.   **Recall period**   - 1/4 participant (M54-SUR-MOD-01-02) recommended highlighting the recall period per module item.   **Timing of completion**   - 1/4 participants (F79-SUR-MIL-01-03) recommended completing item one of the eye severity module later in the day instead of first thing in the morning for more accurate responses.   **Font size**   - 1/4 participants (M33-OPH-MIL-01-04) recommended making the font size bigger and a different colour from the rest of the text and also to add a phrase that prompts participants to select a response per item.   **Response scale**   - 1/4 participants (F55-NOP-SEV-01-06) recommended a different response scale instead of the numeric one. - 7/10 participants did not recommend any changes. | *“Um, no. The only thing I might, uh, say is that as you mention, you know, sometimes the question was, you know, four hours, twelve hours. To make sure that people are reading, you know, the number correctly, maybe make that number a little bigger or have it in, you know, bright red or bright, bright blue or something just to make sure that people—because, you know, when you do something frequently day in, day out, after a while you don’t really read it. You don’t really pay attention. You just kind of do it. And if the numbers are going to change, then it might be good to make that bold or big or colourful just to help people make sure that they are really, um, answering the questions correctly.”* (M54-SUR-MOD-01-02)  *“Um, there's one thing. It's not so much the wording, but it's the timing. Because this is, uh, the first interview of the day. Well for me, it was when I was waking up, getting up. And when I wake—get up, I don’t have any eye pain because I've been resting. You know, my eyes have been resting. So it's, uh, is—I always answer that question, excuse me, no eye pain because I'm just waking up.”* **Interviewer: *“Okay. Thank you. And do you think if you were to, um, answer that question, um, a couple of hours after waking up that your response would be different?”*** *“Yes. It could—it might, it might be different.”* (F79-SUR-MIL-01-03)  **Interviewer: *“And, and if you could just let me know, um, how easy or difficult do you find it to read and answer that question?”*** *“Um, it's very easy to answer the question. Um, it's pretty easy to read it as well. Um, maybe make it a little larger. I don’t know. I'm thinking a little larger. It's just something about—or a different colour scheme actually now that I'm looking at it. It's just something about that black and white that's, I don’t know.”* (M33-OPH-MIL-01-04)  **Interviewer: *“Okay. Thank you. And, um, is there—do you think there would have been, um, kind of better options that we could have included there instead?”*** *“Hmm, well I don’t know. I mean if it's like some—if instead of the numbers like some eye pain, a lot of eye pain, like, like that instead of the numbers.”* (F55-NOP-SEV-01-06) |
|  | **COP-Q Eye Pain Frequency Module** | - 3/10 participants recommended changes to this module.   **Response options**   - 1/3 participants (F64-OPH-MOD-01-01) reported that they would replace the ‘a lot’, ‘some’, ‘a little’ response options on the screen with percentages instead as this would make the selection of the responses a bit easier. - 1/3 participants (M33-OPH-MIL-01-04) mentioned that the response options were ‘ambiguous’ and they recommended to make them more defined, however they did not report having any issues selecting a response.   **Font**   - 1/3 participants (M54-SUR-MOD-01-02) mentioned that they would highlight, or bolden, the recall period required per module so that it is easier for participants to notice it.   **Text colour**   - 1/3 participants (M33-OPH-MIL-01-04) recommended that the questions could be a different colour from the rest of the text throughout so that it is easier for the participants to spot them, however this participant did not have any issues answering the questions. - 7/10 participants did not recommend any changes. | **Interviewer: *“And is there anything that you would reword, um, about that question to make it easier to understand?”*** *“I think instead of using none of the time, a little of the time, some of the time, a lot of the time, all of the time, I might have used percentages. Like 100% of the time, 75% of the time, 50% of the time, 25% of the time, or none of the time—or zero of the time.”*  **Interviewer: *“…And you think that would have been, um, easier?”*** *“Yeah. 'Cause I was a lot of times, well some of the time or a little of the time. Probably not all of the time or none of the time and maybe not a lot of the time.”* (F64-OPH-MOD-01-01)  **Interviewer: *“…And is there anything that you would change about that screen at all, um, and how you select an answer?”*** *“Nothing that I would change except—and again, I kind of mentioned this before, that if you're going to have switching from four hours to 12 hours or 24 hours, if the numbers are going to change in the question at the top, perhaps there would be a good way to highlight that in a colour or size or boldness. Just again as a reminder to people that, you know, look here because the number may or will change based on the question.”* (M54-SUR-MOD-01-02)  **Interviewer: *“Yeah. Perfect. So, um, how easy or difficult, um, do you find it to read and answer that question?”*** *“Um, same as before. Again, I would just make the question, um, a different colour. Like I feel like it's possible that someone just like might not even look up there or think it's part of the other, um, information, like the log-in information. I don’t know. It's just something about it. The question itself doesn’t stand out to me that much. Like I can clearly see it, but I can see someone just simply looking at the answers and they're like, what am I answering.”* (M33-OPH-MIL-01-04)  **Interviewer: *“And what do you think of those response options?”*** *“Um, they're very—they're a little ambiguous. Maybe a little subjective. But at the same time, it kind of covers all your bases too, so sorry. I know that was a bad answer… Maybe, maybe—sorry. Maybe keep these answers like they are, but maybe like put in parentheses like all of the time it's like in that 24 hours, is that like at least 20 plus hours. Like is a little of the time two hours a day at least? Like what is some of the time, like kind of clear it up a little bit there.”* (M33-OPH-MIL-01-04) |
|  | **COP-Q Symptom Module (4-hour recall period) - Eye pain** | - 2/10 participants recommended changes to be made.   **Recall period**   - 1/2 participants (F74-SUR-MOD-01-09) recommended changing the recall period to 24 hours as they didn’t experience much eye pain over the past 4 hours, until they ‘focus’ on what they are doing.   **Visual aspects of item text**   - 1/2 participants(M33-OPH-MIL-01-04) recommended adding more space between the item stem and the item itself, as some readers may ignore the stem. They also recommended changing the colours from black and white. - 8/10 participants did not recommend any changes. | *“I would, would change some of it, because it says the past 24 hours, you know, over the past 4 hours, with your eye pain, so you know, you’re not having too much eye pain until you’re up focused and see what’s going on in the morning times, and at 7 o’clock, you know, you might have just a little bit of irritation, but you won’t have eye pains until you really focus on what you are doing.”* **Interviewer: *“Okay, I see what you mean, okay, so maybe, okay, so then you would change the recall period then, is that what you’re saying, yeah?”*** *“Yes.”* (F74-SUR-MOD-01-09)  *“...Um, it was very easy to read it, but maybe put less space between the first portion and where it says one, eye pain. Um, because just like then, maybe someone would just totally ignore the part where it says please answer the following questions thinking about the symptoms at the time at its worst and just read one, eye pain. Please rate the severity.”* (M33-OPH-MIL-01-04)  **Interviewer: *“Great. Thanks. And is there anything you would change about that screen or how you select an answer at all?”*** *“Um, other than the colour, the black and white colour that I mentioned earlier and putting less space between the first portion of the question and the second portion, um, no.”* (M33-OPH-MIL-01-04) |
|  | **COP-Q Symptom Module (4-hour recall period) - Eye tiredness** | - 1/10 participants (F79-SUR-MIL-01-03) recommended making a change to the item.   **Response options**   - The participant (F79-SUR-MIL-01-03) recommended including an additional response option, ‘not applicable’ as they were unsure what eye tiredness meant. - 9/10 participants did not recommend any changes. | **Interviewer: *“And is there anything that you would change about, um, the question you've currently got on your screen and how you select an*** ***answer?”*** *“Uh, the only, uh, question I had about, uh, the question was not burning of the eye. Eye irritation. Eye tiredness. Uh, I think I always chose zero because I wasn’t sure how to answer that question. Maybe it should have, uh—could have had an option for not applicable.”* (F79-SUR-MIL-01-03) |
|  | **Visual Tasking Module (VTM) - Instructions** | - 3/10 participants recommended changes to be made to the VTM.   **Font size**   - 3/10 participants recommended that the font size could be a bit larger, however they did not have any problem reading the text. - 7/10 participants did not recommend any changes. | *“I mean to me bigger is always better. But, you know, I… perhaps would..make, you know, all of these, you know, a, a little bigger, just to make it more readable. But, but it's, it's fine the way it is..”* (M54-SUR-MOD-01-02) |
|  | **Health-related Quality of Life (HRQoL) Module – Low or depressed** | - 3/10 participants recommended changes to make to the item.   **Reword item text**   - 3/3 participants reported that they would reword the text of item one. - 1/3 participants (F79-SUR-MIL-01-03) noted that they would substitute the words ‘*low’* and *‘down’* of the item with the word *’down’*. - 2/3 participants mentioned that they would make the wording of the item more specific to eye pain. - 7/10 participants did not recommend any changes. | **Interviewer: *“And is there anything that you would reword to make it easier to understand?”*** *“Hmm, I guess instead of the word low, down. I would substitute it for down.”* (F79-SUR-MIL-01-03)  *“Cause I was just wondering what did the feeling low and depressed, what would that have to do with eye pain?”* **Interviewer: *“So do you think—would you say that that's something that's to relevant to your eye pain?”*** *“Yes. That's what I think.”* (F55-NOP-SEV-01-06) |
|  | **Health-related Quality of Life (HRQoL) Module- Frustrated** | - 1/10 participants recommended changes to make to the item.   **Reword item text**   - The participant reported that they would reword the text of item three and make it more specific to eye pain (F74-SUR-MOD-01-09). - 9/10 participants did not recommend any changes. | *“How much of the time do you feel frustrated? Um, that was a little of the time, because I feel like problems come up and everything, and I’m just trying to figure out, well, how can I do this, how can I do that, and everything, so it did, you know, frustrate me a little bit and everything, but still like I said, with this question and everything, it should be more of your problem with your eyes than with everyday living life.”* (F74-SUR-MOD-01-09) |
|  | **Health-related Quality of Life (HRQoL) Module- Worried** | - 1/10 participants recommended a change to be made to the item (F74-SUR-MOD-01-09).   **Wording of the item**   - 1/10 participants reported as a personal preference for the item to be more specific to eye pain. However, they noted that they would not reword the item (F74-SUR-MOD-01-09). - 9/10 participants did not recommend any changes. | *“Well, because, you know, some people do worry about their eyes and everything, because sometimes I worry about mine, why I’m having, you know, eye irritation and why my eyes are getting so tired and all that and everything, then it dawned on me, well, because I’m trying to do too much with my eyes sitting there, which I need to sit down and close my eyes or rest my eyes some, so I think, um, that question should be more like are you worried, worried about your eyesight or what’s going on with your eyes… So I would, I would leave that just like that, um, but then too, everybody might not be thinking the way I’m thinking.”* (F74-SUR-MOD-01-09) |
| Changes to patient-facing text of the ePRO | **Quit button** | - 4/10 participants recommended changes to be made to the quit button.   **Text**   - 4/4 participants recommended that the text of the quit button could be larger, however these participants did not experience any issues reading the text inside the quit button.   **Button size**   - 1/4 participants (M62-OPH-MIL-01-10) recommended that the quit button should be made either bigger or brighter to be easier to locate. However, this participant had no issue locating the quit button once they were asked to by the interviewer   **Response options**   - 1/4 participants (M33-OPH-MIL-01-04) also recommended that the two response options of ‘Confirm’ or ‘Cancel’ that are given to the user once they click on the quit button could be made bolder so that it is easier for the user to select a response. However, this participant had no issue selecting one of these two response options. - 6/10 participants did not recommend any changes for the quit button. | *“…But maybe like put a bolder box around those options just to make it stand out that there are options that are present that might be important.”* (M33-OPH-MIL-01-04)  **Interviewer: *“Okay. Um, so was it, was it easy or hard to find that quit button?”*** *“Uh, it was—looked quite small up at the top, um, so I had to kind of, you know, look over the screen to see where there might be the opportunity to, you know, maybe cancelling and stuff. Maybe not quite too—it's not really the clearest.”* **Interviewer: *“Okay. So, okay. So would, would you recommend that to be a bit bigger then or…?”*** *“Either bigger or maybe a little brighter.”* (M62-OPH-MIL-01-10) |
|  | **Skip button** | - 3/10 participants recommended changes to the skip button.   **Text size**   - 3/3 participants recommended that the text of the skip button could be larger, however they did not experience any issues reading the instruction.   **Instructions**   - 1/3 participants (M33-OPH-MIL-01-04) also recommended that it would be useful to be provided with some information about where the participant should click to skip a question. - 7/10 participants did not recommend any changes. | **Interviewer: *“Okay. Great. And, um, is the font size large enough in that textbox?”*** *“Um, it could be a little larger, yes. I think it would help, especially for an eye study.”* (M56-OPH-MIL-01-07)  **Interviewer: *“And, um, if you wanted to skip this question, um, is it clear what you would need to do?”*** *“Um, no. It's not clear what you would need to do. Um, a few clicking around would probably easily get someone there, but there's no, you know, dialogue that says click next to skip, which could be added to make that clear.”* (M33-OPH-MIL-01-04) |
|  | **Help button** | - 1/10 participants (F64-OPH-MOD-01-01) recommended making a change to the help button.   **Instructions**   - 1/10 participants (F64-OPH-MOD-01-01) recommended that it would be helpful if there was an instruction or guidance provided explaining that the question mark on the screen was the help button, as this was not clear. - 9/10 participants did not recommend any changes. | **Interviewer: *“Great. Thank you. And, um, have you needed to use that button during the past four-weeks at all?”*** *“No. Cause I didn’t know what it was for.”* **Interviewer: *“Yeah. And do you think it would have been helpful to, to have known that that button was there?”*** *“Um, yeah, I do.”* (F64-OPH-MOD-01-01) |

# Discussion and conclusion

The overall objective of this study was to conduct qualitative usability interviews with COSP patients to assess usability and support refinement of the ePRO and PGI items on a tablet device. This was the first part of a larger observational study, designed to evaluate and document the psychometric properties of the ePRO and PGI items with consideration of item reduction.

To achieve this aim, N=10 COSP patients were recruited in the US to take part in a 45-minute usability interview, after completing a four-week data collection period, during which they had to complete daily and weekly assessments of the ePRO and PGI items using a tablet device.

## Usability interviews findings

### General usability of the ePRO

All participants reported that the ePRO tablet device was easy to use and reported that the aspects they liked the most were the daily alarms/reminders, the concise and clear instructions, the responsive interface and ease of navigation, the large formatting of the text and the long response windows. All participants also reported they were able to complete the COP-Q at their home and that it was easy fitting the completing of the questionnaires twice a day on their daily schedule. The completion of the COP-Q was a quick task to do, as half of the participants reported that the maximum time it would take them to complete it on a daily basis was five minutes or less and the other half reported that it would be up to 10-15 minutes. No participants reported that they needed to take a break while completing the questionnaires.

Participants were also asked about their preference in completing the COP-Q either twice a day (4-hour recall period) or once a day (24-hour recall period). Half of participants reported that they preferred completing the COP-Q twice a day as it was easier to remember their eye pain during the past 4 hours, and half reported that they preferred completing the COP-Q once per day as this proved less bothersome to fit into their daily schedule. Of note, the two recall period versions of the COP-Q (4-hour and 24-hour) will be compared in terms of measurement properties and score comparability and it will be ensured sufficient data is generated that both versions can be validated independently further in the psychometric validation stage of the observational study. The ultimate aim is to select one of the two recall period versions for use in future clinical studies.

Participants reported that the morning (7:00am to 10:00am) and the evening (18:00pm to 23:00pm) response time windows were quite long and easy to comply with. The daily alarms/reminders were described as very helpful, although the majority of participants reported that they had missed at least one data entry during the four-week data collection period. Reasons for missing dairy entries including forgetting or family emergencies. Most participants had not missed more than one entry.

All participants reported that it was easy to charge the tablet device at home during the four-week data collection period and the majority reported that the tablet’s touch screen was very responsive. Furthermore, all participants thought that the training provided to them by the recruitment site on how to use the tablet device upon their enrolment in the study was useful and informative.

However, despite the completion of the ePRO and PGI items on the tablet device being an overall positive experience, some participants reported aspects of the tablet device they disliked, technical difficulties they experienced during the four-week data collection period and provided recommendations on the tablet device. Aspects of the tablet device that more than one participant disliked included the login pin code, the alarm/reminders (specifically that these did not function properly on occasion), and that the completion of the ePRO would take a longer time to complete some days (on the days that the VTM, HRQoL modules and PGI items were scheduled to be completed alongside the Eye Pain Severity, Eye Pain Frequency and Symptom Module).

Issues with alarm/reminders on the tablet device and the login pin code were reported by half of the participants. Specifically, participants reported that the alarms/reminders would not always go off at the time they were set for, or the volume would be too low to hear. Participants therefore recommended that a second alarm should be provided. Participants reported that the login pin code would not always work the first time they entered this, and they had to re-enter it a few times for it to provide them with access to the ePRO.

Some participants noted that the response time windows for the evening assessments of the COP-Q were shorter in duration than the morning ones and suggested that this should be changed so that they are of the same duration. However, none of these participants reported missing a diary entry or any issues completing the ePRO due to the difference in duration between the morning and evening time windows.

### Debriefing of the COP-Q and PGI items

For all COP-Q modules, the majority of participants reported that it was easy to read the text, understand the instructions or item and select a response. It should be noted that the purpose of this report was to evaluate the usability of the ePRO and not to explore it’s content validity as content validity of the COP-Q was explored during the previous qualitative work. Therefore, some item properties such as response options and recall period were not always debriefed for each item of the COP-Q or for each participant.

The following recommendations were made in relation to the COP-Q modules or items themselves by more than one participant: providing an option to select percentages rather than responding on a numerical scale or a verbal rating scale; making the font size of the text on the VTM slightly larger and rewording some of the HRQoL questions to make it a bit clearer that the items are referring to their eye pain. Again, none of these participants reported experiencing any issues when selecting a response for the items.

Participants were also debriefed on the ‘patient facing text’ from the pop-up boxes which explained how to quit, skip, and look for help when using the ePRO. All participants reported that the pop-up boxes were clear to understand and that they did not need to use them during the four-week data collection period. For all participants, the quit button was easy to find. However, a few participants reported that the skip and help buttons were not easy to locate. These participants recommended making the buttons bolder, larger or of a different colour so that future participants can spot them easily. Participants also recommended that prompts for how to skip a question and additional explanation that the help button is represented with a question mark on the screen could be added to the ePRO to aid participants locating the skip and help buttons. It is worth noting that no participants report difficulties with reading the ‘patient facing text’. Recommendations were mostly based on personal preferences and considering what could be helpful for future participants.

### AV PCO recommendations

Although the majority of participants’ recommended changes did not impact their ability to use the tablet device and/or respond to the ePRO, AV PCO identified some areas which could potentially be improved for the benefit of future clinical trial participants:

- Increasing the font size of the ‘patient facing text’ in the pop-up boxes for future studies, given that some future participants might have issues reading the text within the boxes, as noted by participants of this study.
- Providing a more comprehensive training session upon participant enrolment to the study to resolve some of the technical issues reported by participants, such as establishing an internet connection on the tablet device, changing the time and volume of the alarms and the location the help, skip and quit buttons. Consideration could be given to providing a recorded version of the training session that participants can refer back to if needed.

To avoid delays in shipping out tablet devices for the next phase of the wider observational study planned to evaluate and document the psychometric properties of the COP-Q and PGI items, AV PCO propose that this more thorough participant training is developed on paper and provided to participants. This is expected to aid participants by allowing them to refer back to this document when needed during the four-week data collection period.

In addition, AV PCO followed up with the third-party vendor responsible for developing the ePRO and PGI items to investigate the alarm/reminders and login pin code issues reported by some participants to determine if this issue was due to faulty devices or if it was a larger issue with the back programming of the devices that will need to be resolved before the second round of the data collection. Upon investigation, the third-party vendor, confirmed that they could not replicate the technical issues that were reported by the participants during the usability interviews. It was decided that the team could move into the second stage of data collection without any further updates needed to the platform.

Regarding increasing the font size of the ‘patient facing text’, participants recommended this update thinking of the potential needs of future participants, rather than because they had issues reading the text during the data collection period. Furthermore, upon discussions with the third-party vendor responsible for developing the ePRO, it was concluded that the implication of completing this update to the study timelines will be greater than initially estimated. Therefore, the development of two patient user guides, the Questionnaire Completion Patient User Guide and the Kayentis Web Platform Use Patient User Guide, which include all the necessary information need by the participants to complete and navigate the ePRO, was suggested as a solution.

## Limitations

It should be recognized that this was a small-scale usability qualitative study, with both the benefits and limitations in terms of sample size that one can typically expect from qualitative research. Thus, while the study design provided considerable depth of insight and descriptions regarding the patient experience when using the ePRO and PGI items, caution should be employed when drawing conclusions, due to the relatively small sample size.

There were instances where participants came across some technical difficulties when using the tablet device that led some of them to missing diary entries and therefore missing data. Further, the data collection period for the usability phase of the study also fell between national holidays for some participants which resulted in a higher percentage of missed diary entries. However, the overall compliance of the first ten participants completing the usability interviews was relatively high and the qualitative findings from the usability interviews indicated that the completion of the COP-Q was an overall easy and quick task to do and that participants could fit in their daily schedule without any issues. This eliminates any concerns as to participants’ compliance for the reasons described above during the second round of data collection for the wider observational study.

Despite the limitations described, this qualitative study followed rigorous and recommended methods to explore the usability of the COP-Q and PGI items in the COSP population.

## Conclusions

In summary, the findings support the usability of ePRO and PGI items and their suitability for use in future clinical trials. It is recommended that a comprehensive training manual is developed by the third-party vendor responsible for developing the ePRO, which will provide guidance to the participants in terms of completing the questionnaires, navigating the tablet and setting up the alarms/reminders and internet connection, to minimize compliance issues and to improve the overall experience of completing the ePRO. This was the first part of a larger observational study specifically aiming to explore the usability of the questionnaire. Further conclusions will be drawn from the next phase of the study aiming to evaluate the psychometric properties of the ePRO and PGI items.

# References

1. Jacobs DS. Diagnosis and treatment of ocular pain: the ophthalmologist’s perspective. *Current ophthalmology reports.* 2017;5(4):271-275.

2. Kalangara JP, Galor A, Levitt RC, Felix ER, Alegret R, Sarantopoulos CD. Burning eye syndrome: do neuropathic pain mechanisms underlie chronic dry eye? *Pain Medicine.* 2015;17(4):746-755.

3. FDA. Guidance for Industry: Patient-Reported Outcome Measures: Use in Medical Product Development to Support Labeling Claims. 2009:1-43.

4. FDA. Patient-Focused Drug Development: Collecting Comprehensive and Representative Input: Guidance for Industry, Food and Drug Administration Staff, and Other Stakeholders. 2020.

5. FDA. Patient-Focused Drug Development: Methods to Identify What Is Important to Patients: Guidance for Industry, Food and Drug Administration Staff, and Other Stakeholders. 2022.

6. FDA. Patient-Focused Drug Development: Selecting, Developing, or Modifying Fit-forPurpose Clinical Outcome Assessments: Guidance for Industry, Food and Drug Administration Staff, and Other Stakeholders 2022.

7. FDA. Patient-Focused Drug Development: Incorporating Clinical Outcome Assessments Into Endpoints For Regulatory Decision-Making: Guidance for Industry, Food and Drug Administration Staff, and Other Stakeholders. 2023.

8. Crane AM, Levitt RC, Felix ER, Sarantopoulos KD, McClellan AL, Galor A. Patients with more severe symptoms of neuropathic ocular pain report more frequent and severe chronic overlapping pain conditions and psychiatric disease. *British Journal of Ophthalmology.* 2017;101(2):227-231.

9. Benatti CA, Afshari NA. Postoperative pain after laser refractive surgery. *International ophthalmology clinics.* 2016;56(2):83-100.

10. Levitt AE, Galor A, Weiss JS, et al. Chronic dry eye symptoms after LASIK: parallels and lessons to be learned from other persistent post-operative pain disorders. *Molecular pain.* 2015;11:s12990-12015-10020-12997.

11. Sanchez-Avila RM, Merayo-Lloves J, Riestra AC, et al. Plasma rich in growth factors membrane as coadjuvant treatment in the surgery of ocular surface disorders. *Medicine.* 2018;97(17).

12. Shtein RM. Post-LASIK dry eye. *Expert review of ophthalmology.* 2011;6(5):575-582.

13. Toda I. LASIK and the ocular surface. *Cornea.* 2008;27:S70-S76.

14. Reilly MC, Zbrozek AS, Dukes EM. The validity and reproducibility of a work productivity and activity impairment instrument. *Pharmacoeconomics.* 1993;4(5):353-365.

15. Coons SJ, Gwaltney, C. J., Hays, R. D., Lundy, J. J., Sloan, J. A., Revicki, D. A., ... & Basch, E. . Recommendations on evidence needed to support measurement equivalence between electronic and paper‐based patient‐reported outcome (PRO) measures: ISPOR ePRO Good Research Practices Task Force report. *Value in Health.* 2009;12(4):419-429.

16. Ajyegbusi OL. Key methodological considerations for usability testing of electronic patient‑reported outcome (ePRO) systems. *QUALITY OF LIFE RESEARCH.* 2020;29:325-333.

17. Fonteyn ME, Kuipers B, Grobe SJ. A description of think aloud method and protocol analysis. *Qualitative health research.* 1993;3(4):430-441.

18. Hsieh H-F, Shannon SE. Three approaches to qualitative content analysis. *Qualitative health research.* 2005;15(9):1277-1288.

19. Joffe H, Yardley L, Marks D. Research methods for clinical and health psychology. *Content and thematic analysis London: Sage.* 2004:56-68.

# Appendix A. ePRO screenshots

1. **COP-Q title page**

1. **COP-Q Eye Pain Severity Module**

1. **COP-Q Eye Pain Frequency Module**

1. **COP-Q Symptom module (4-hour recall period)**

1. **COP-Q Symptom module (24- hour recall period)**

1. **COP-Q Visual Tasking Module**

1. **COP-Q Health-related Quality of Life Module**

1. **COP-Q ‘Patient facing text’**

1. **PGI-S Items**

1. **PGI-C Items**

# Appendix B- COP-Q

**‘Eye Pain Severity’ Module**

| **1. Eye pain Severity** | Please rate the severity of your eye pain **at its worst** over the **past 4 hours:**  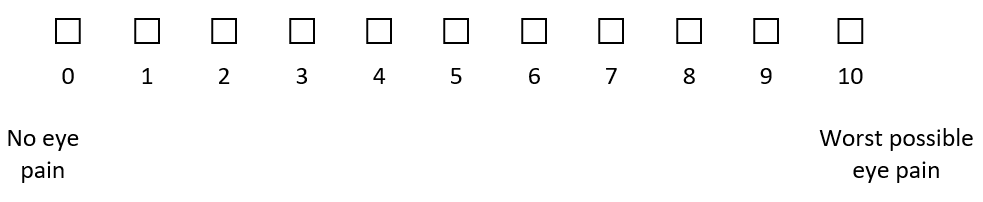 |
| --- | --- |

**‘Eye Pain Frequency’ Module**

| **1. Frequency** | How much of the time have you had eye pain over **the past 24 hours**?   \| 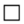 \| 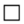 \| 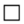 \| 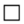 \| 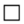 \|  \| \| --- \| --- \| --- \| --- \| --- \| --- \| \| None of the time \| A little of the time \| Some of the time \| A lot of the time \| All of the time \| |
| --- | --- | --- | --- | --- | --- | --- | --- | --- | --- | --- | --- | --- |

| **1. Eye pain** | Please rate the severity of your eye pain **at its worst** over the past 4 hours:  **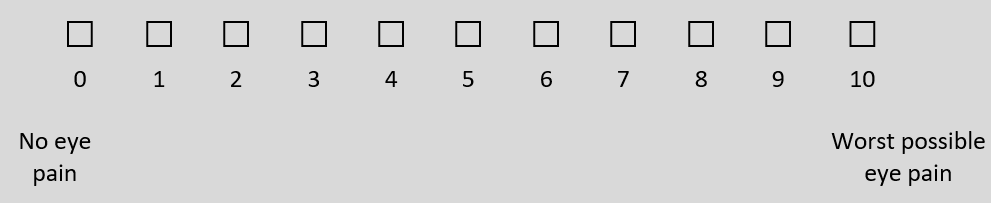** |
| --- | --- |
| **2. Eye irritation** | 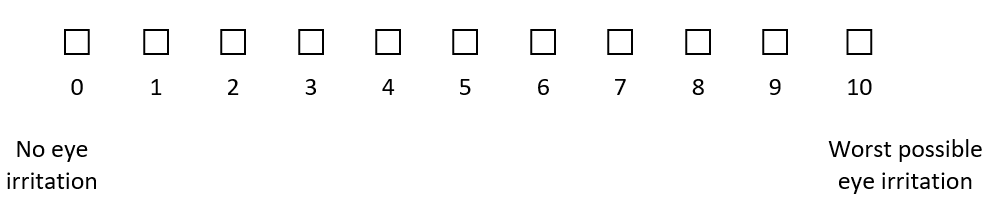Please rate the severity of your eye irritation **at its worst** over the past 4 hours: |
| **3. Burning of the eye** | Please rate the severity of any burning feelings in your eye(s) **at its worst** over the past 4 hours:  **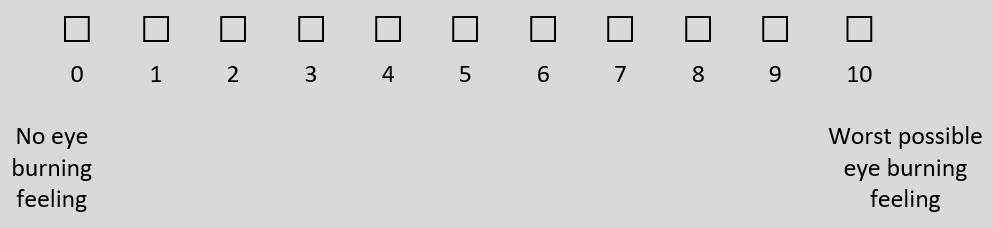** |
| **4. Eye tiredness** | Please rate the severity of your eye tiredness **at its worst** over the past 4 hours:  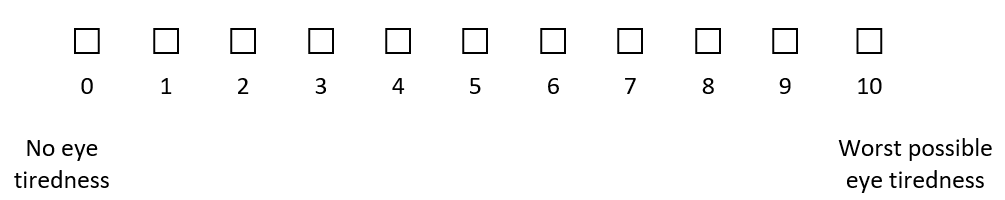 |
| **5. Eye dryness** | Please rate the severity of your eye dryness **at its worst** over the past 4 hours:  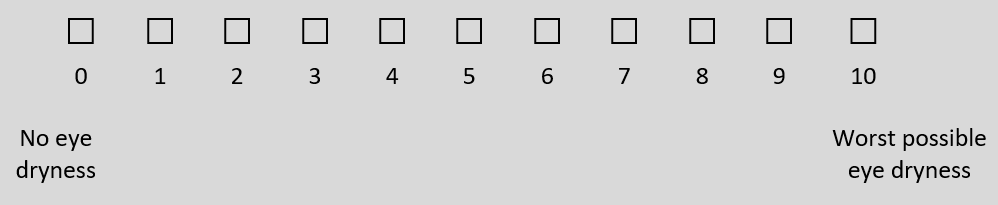 |

| **6. Feeling like there is something in your eye** | Please rate the severity of a feeling that you have something in your eye **at its worst** over the past 4 hours:  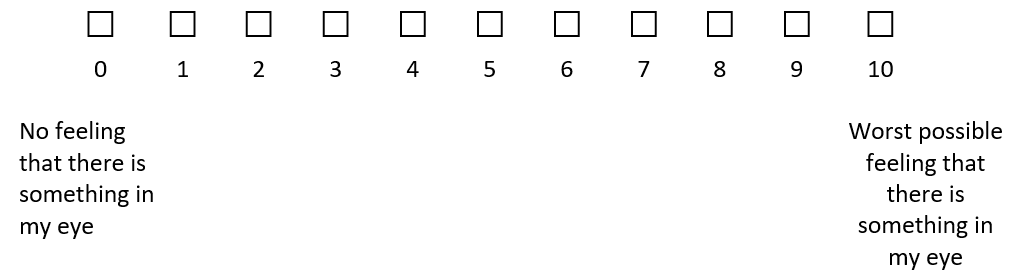 |
| --- | --- |
| **7. Eye itch** | Please rate the severity of your eye itch **at its worst** over the past 4 hours:  **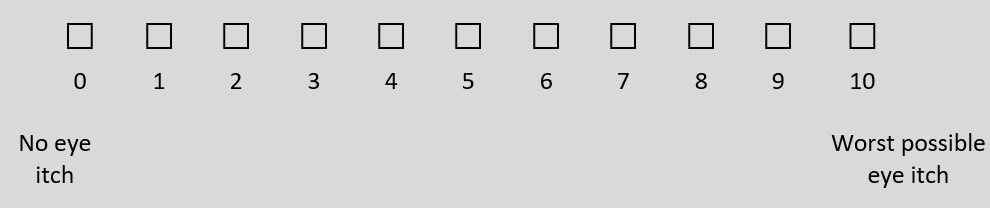** |

**Alternative: Symptom Module**

Please answer the following questions thinking about each symptom at the time it was **at its worst** over the past 24 hours.

| **1. Eye pain** | Please rate the severity of your eye pain **at its worst** over the past 24 hours:  **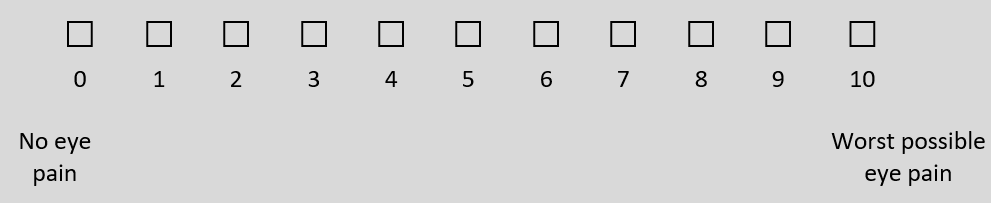** |
| --- | --- |
| **2. Eye irritation** | 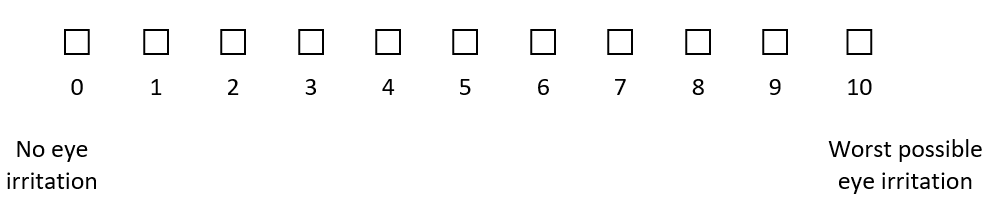Please rate the severity of your eye irritation **at its worst** over the past 24 hours: |
| **3. Burning of the eye** | Please rate the severity of any burning feelings in your eye(s) **at its worst** over the past 24 hours:  **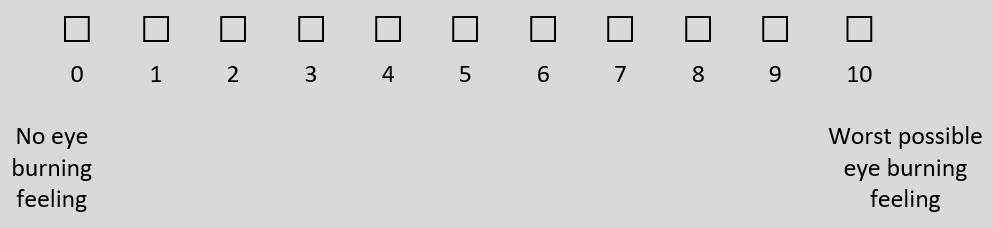** |
| **4. Eye tiredness** | Please rate the severity of your eye tiredness **at its worst** over the past 24 hours:  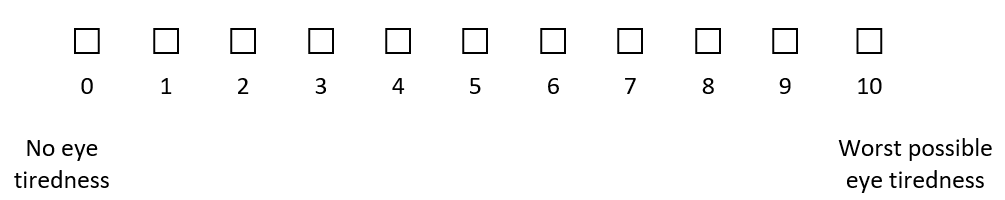 |
| **5. Eye dryness** | Please rate the severity of your eye dryness **at its worst** over the past 24 hours:  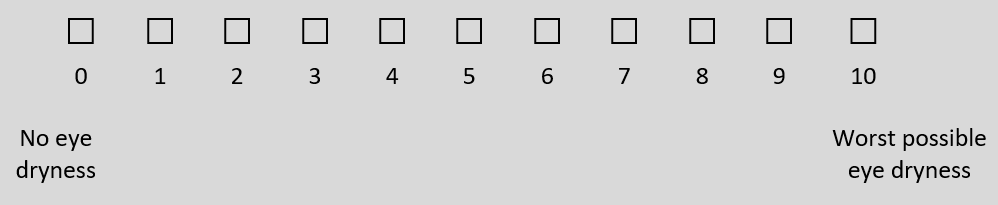 |
| **6. Feeling like there is something in your eye** | Please rate the severity of a feeling that you have something in your eye **at its worst** over the past 24 hours:  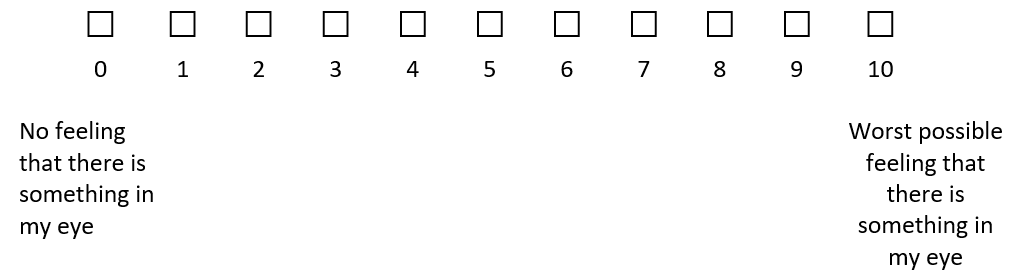 |
| **7. Eye itch** | Please rate the severity of your eye itch **at its worst** over the past 24 hours:  **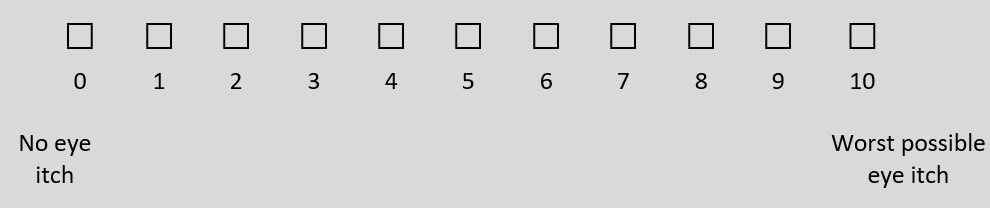** |

**Visual Tasking Module**

The following questions ask about how much of the time your **eye pain and related problems (e.g., blurriness) affected your ability to do visual activities over the past 7 days.** Please do not think about any other vision problems you have (such as difficulties seeing things up close or at a distance) when selecting an answer.

Difficulties doing visual activities might include **changing how you did an activity, avoiding an activity, or needing to take a rest from an activity** because of your eye pain and related problems.

| Over the past 7 days how much of the time did your eye pain and related problems affect your ability to… | None of the time | A little of the time | Some of the time | A lot of the time | All of the time | I avoided or was completely unable to do this activity due to my eye problems | I did not do this for reasons unrelated to my eye problems |
| --- | --- | --- | --- | --- | --- | --- | --- |
| 1. Read books, newspapers or magazines for more than one hour? |  |  |  |  |  |  |  |
| 2. Read on a screen for example a computer or tablet for more than one hour? |  |  |  |  |  |  |  |
| 3. Watch a program on the TV for more than one hour? |  |  |  |  |  |  |  |
| 4. Watch events at a distance for example a show or sporting event? |  |  |  |  |  |  |  |
| 5. Drive at night? |  |  |  |  |  |  |  |
| 6. Drive during the day? |  |  |  |  |  |  |  |
| 7. Look in the mirror for example to shave or put your make up on? |  |  |  |  |  |  |  |
| 8. Carry out your usual leisure activities or hobbies for example crafts, painting, playing cards? |  |  |  |  |  |  |  |

**Health-Related Quality of Life Module**

The following questions ask about **ways your eye pain and related problems (e.g., blurriness) may have affected you over the past 7 days.**

| Over the past 7 days… | | None of the time | A little of the time | Some of the time | A lot of the time | All of the time |
| --- | --- | --- | --- | --- | --- | --- |
| 1 | How much of the time did you feel low or depressed? |  |  |  |  |  |
| 2 | How much of the time did you feel anxious? |  |  |  |  |  |
| 3 | How much of the time did you feel frustrated? |  |  |  |  |  |
| 4 | How much of the time did you feel worried? |  |  |  |  |  |
| 5 | How many nights did your eye pain and related problems affect your sleep? | - 0 nights - 1-2 nights - 3-4 nights - 5-6 nights - Every night | | | | |

For each question, please choose the answer which describes how much of the time you were affected **because of your eye pain and related problems over the past 7 days.**

# Appendix C. PGI items

**Global Impression of Severity**

**1.** Please choose the response below that best describes the overall severity of your **eye pain** over the **past 7 days** (select one response).

□ Severe

□ Moderate

□ Mild

□ None

**2.** Please choose the response below that best describes the overall severity of your **eye pain and related problems (e.g., itch, irritation, blurriness)** over the **past 7 days** (select one response).

□ Severe

□ Moderate

□ Mild

□ None

**3.** Please choose the response below that best describes the overall severity of your **limitations in carrying out visual activities (e.g., reading, watching the TV)** over the **past 7 days** (select one response).

□ Severe

□ Moderate

□ Mild

□ None

**Global Impression of Change**

**1.** Please choose the response below that best describes the overall change in your **eye pain** compared to when you started this study (select one response).

□ Much better

□ A little better

□ No change

□ A little worse

□ Much worse

**2.** Please choose the response below that best describes the overall change in your **eye pain and related problems (e.g., itch, irritation, blurriness)** compared to when you started this study (select one response).

□ Much better

□ A little better

□ No change

□ A little worse

□ Much worse

**3.** Please choose the response below that best describes the overall change in your **limitations in carrying out visual activities (e.g., reading, watching the TV)** compared to when you started this study (select one response).

□ Much better

□ A little better

□ No change

□ A little worse

□ Much worse

1. Two participants (M54-SUR-MOD-01-02; F79-SUR-MIL-01-03) hadn’t attempted to find the ‘quit’ button before the interviewer asked them to so they hadn’t realised the button was there before this point [↑](#footnote-ref-2)
