# Supplementary material for: Psychometric validation of the Chronic Ocular Pain Questionnaire (COP-Q)
Source: J Patient Rep Outcomes. 2025 Mar 12;9:32. doi: 10.1186/s41687-025-00862-9 (PMC11903982; doi:10.1186/s41687-025-00862-9)
Supplement: Supplementary file 2 — Supplementary Material 2 [file 41687_2025_862_MOESM2_ESM.docx]

## Supplementary 2. Study eligibility criteria and target sampling quotas

Table 1. Study eligibility criteria

| **Inclusion criteria** |
| --- |
| 1. Patient is an adult aged 18 or above |
| 1. Patient has symptoms of COSP (chronic, persistent eye pain [can also be described as other symptoms e.g. burning, irritation, dryness etc.] at the ocular surface lasting for more than three months at screening), irrespective of treatment |
| 1. The primary complaint is ocular pain coming from the surface of the eye [corneal or conjunctiva rather than systemic pain] |
| 1. On average, the patient experiences ocular pain at least four days per week in a typical week |
| 1. Fluent speaker, literate and able to read and write in the English language |
| 1. Willing and able to provide written or electronic informed consent and to perform all study activities |
| **Exclusion criteria** |
| 1. Patient has an active ocular infection |
| 1. Patient is participating in another observational study or clinical trial |
| 1. Patient experiences acute seasonal ocular allergies during the time they would be participating in the study |
| 1. Patient has any other physical or mental illness that might influence the responses they give to questions about their ocular pain or might impact the patient’s ability to participate in the study |

* COSP diagnosis and patient selection was based on clinician assessment to differentiate true ocular pain from centralized/phantom pain

Table 2. Target sampling quotas

| **Patient characteristics** | | **Total target (N=120)** |
| --- | --- | --- |
| Gender | Female | ≥40 |
|  | Male | ≥40 |
| Age | 18-35 years old | ≥20 |
|  | 36-60 years old | ≥30 |
|  | >60 years old | ≥30 |
| Race | Non-Caucasian | ≥30 |
|  | Caucasian | ≥30 |
| Level of education | High school or less | ≥30 |
|  | More than high school | ≥30 |
| COSP severity level | Severe (pain score of 7-10 on a 0-10 scale, past week recall) | ≥40 |
|  | Moderate (pain score of 4-6 on a 0-10 scale, past week recall) | ≥40 |
|  | Mild (pain score of 1-3 on a 0-10 scale, past week recall) | ≥15 (no more than 24) |
| Diagnosis | Diagnosed with an ophthalmological condition (e.g., DED, MGD, blepharitis etc.) and no history of refractive surgery or systemic comorbidities | ≥30 |
|  | Has had refractive eye surgery (e.g., LASIK, PRK, etc.) | ≥30 |
|  | Patients with non-ophthalmic underlying conditions (e.g., Sjögren's syndrome, diabetes, rheumatoid arthritis etc.) | ≥30 |
