# Supplementary material for: Psychometric validation of the Chronic Ocular Pain Questionnaire (COP-Q)
Source: J Patient Rep Outcomes. 2025 Mar 12;9:32. doi: 10.1186/s41687-025-00862-9 (PMC11903982; doi:10.1186/s41687-025-00862-9)
Supplement: Supplementary file 3 — Supplementary Material 3 [file 41687_2025_862_MOESM3_ESM.docx]

## Supplementary 3. Overview of additional PRO assessments

### PGI items

#### Patient Global Impression of Severity (PGI-S)

COSP patients completed three Patient Global Impression of Severity (PGI-S) questions weekly during the data collection period. These PGI-S items were designed to capture patients’ perception of the overall severity of their eye pain, related symptoms, and limitations in carrying out visual activities, respectively, over the ‘past 7 days’. These items utilise a 4-point categorical response scale. A higher score indicates greater severity. None of the PGI-S items were reverse scored.

#### Patient Global Impression of Change (PGI-C)

The Patient Global Impression of Change items (PGI-C) are three items which are designed to capture patients’ perception of the overall change in their eye pain, related symptoms, and limitations in carrying out visual activities, respectively, since the start of the study until the time of completion. Similar to the PGI-S items, the PGI-C items utilise a 4-point categorical response scale. A higher score indicates greater severity. None of the PGI-C items were reverse scored.

### Visual Functioning Questionnaire (VFQ-25)

The Visual Functioning Questionnaire (VFQ-25) is a well-validated measure of eye pain and discomfort and vision-related activities, which was administered to assess the convergent validity of the COP-Q. The VFQ-25 consists of 25 items which are designed to capture patients’ general health and vision, difficulty with activities and responses to vision problems. Items are answered using a mixture of 2-point, 3-point, 4-point, 5-point and 6-point verbal response scales. A specific recall period is not specified for the VFQ-25 – patients are asked to think about their health, vision, eye pain and functioning ‘in general’. A higher score represents better functioning. No items are reverse scored in the VFQ-25.

### EuroQoL-5 Dimension-5 Level (EQ-5D-5L) with vision bolt on

The EuroQoL-5 Dimension-5 Level (EQ-5D-5L) is a well-validated measure of quality of life which was administered as part of this study to assess the convergent validity of the COP-Q. The EQ-5D-5L consists of five items which assess mobility, self-care, usual activities, pain/discomfort and anxiety/depression, with a recall period of ‘today’. There is also the EQ vertical visual analogue scale (EQ VAS) on which the individual is asked to rate their overall health on a scale with the anchors of – 0 (‘The worse health you can imagine’) to 100 (‘The best health you can imagine’). A bolt-on item assessing vision ‘today’ will also be administered as part of the EQ-5D-5L for the purposes of this study. Items are answered on a 5-point categorical response scale. A higher score indicates greater severity. No items are reverse scored in the EQ-5D-5L.

### Ocular Pain Assessment Survey (OPAS)

The Ocular Pain Assessment Survey (OPAS) is a validated measure of eye pain and discomfort, which will be administered to assess the convergent validity of the COP-Q. The OPAS consists of 27 items assessing eye pain, general pain, quality of life, eye pain aggravating factors, eye pain associated factors and symptom relief, across recall periods of ‘today’, the ‘past 24-hours’ and the ‘past 2 weeks’. Items are answered on a 0–10 NRS ranging from ‘No pain’ (0) to ‘Severe pain’ (10) for items assessing eye pain and general pain. Items assessing quality of life are answered on a 0–10 NRS ranging from ‘Not at all’ (0) to ‘Completely’ (10), and items assessing eye pain aggravating factors, eye pain associated factors and symptom relief are scored on a 0-100% scale ranging from ‘Never’ (0) to ‘All the time’ (100%). A higher score indicates great severity. No items are reverse scored in the OPAS.
