# Supplementary material for: Psychometric validation of the Chronic Ocular Pain Questionnaire (COP-Q)
Source: J Patient Rep Outcomes. 2025 Mar 12;9:32. doi: 10.1186/s41687-025-00862-9 (PMC11903982; doi:10.1186/s41687-025-00862-9)
Supplement: Supplementary file 4 — Supplementary Material 4 [file 41687_2025_862_MOESM4_ESM.docx]

**Supplementary 4. Analysis populations**

### Full analysis population

The full analysis population includes all patients enrolled into the observational study (N=124).

### Psychometric analysis population

The psychometric analysis sample was used for all analyses unless specified otherwise, and incorporated all patients enrolled into the study with at least one item competed on the COP-Q at any time point.

### Test-retest analysis population

The test-retest analysis populations are outlined below. Due to the alternating study design the Eye Pain Severity and Symptom Module samples have been combined for the following daily and 7-day average score analyses.

Table 1. Test-retest analysis populations

| **Test-retest population** | **COP-Q domain score** | **Time points** | **Measure of stability** | **Definition of stability** |
| --- | --- | --- | --- | --- |
| TRTAP_1 | Eye Pain Severity Module (4-hour recall period; AM and PM) | **Daily:** Day 2 – Day 16 or Day 9 – Day 23 (dependant on randomization)  **7-day average:**  Between Week 1 and Week 3 or Week 2 and Week 4, depending on when completed for each given patient | Eye pain PGI-S | Eye pain PGI-S change = 0 |
| TRTAP_2 | Eye Pain Frequency Module (24-hour recall period) | **Daily:** Day 2 and Day 16  **7-day average:** Between Week 1 and Week 3 | Eye pain PGI-S | Eye pain PGI-S change = 0 |
| TRTAP_3 | Symptom Module (4-hour recall period version; AM and PM) | **Daily:** Day 2 – Day 16 or Day 9 – Day 23 (dependant on randomization)  **7-day average:**  Between Week 1 and Week 3 or Week 2 and Week 4, depending when completed for each given patient | Symptom PGI-S | Symptom PGI-S change = 0 |
| TRTAP_4 | Symptom Module 24-hour recall period version | **Daily:** Day 2 – Day 16 or Day 9 – Day 23 (dependant on randomization)  **7-day average:**  Between Week 1 and Week 3 or Week 2 and Week 4, depending on when completed for each given patient | Symptom PGI-S | Symptom PGI-S change = 0 |
| TRTAP_5A | VTM (7-day recall period) | Between Week 1 and Week 2 | Visual tasking PGI-S | Visual tasking PGI-S change = 0 |
| TRTAP_5B | VTM (7-day recall period) | Between Week 1 and Week 3 | Visual tasking PGI-S | Visual tasking PGI-S change = 0 |
| TRTAP_6A | HRQoL Module (7-day recall period) | Between Week 1 and Week 2 | Symptom PGI-S | Symptom PGI-S change = 0 |
| TRTAP_6B | HRQoL Module (7-day recall period) | Between Week 1 and Week 3 | Symptom PGI-S | Symptom PGI-S change = 0 |
| TRTAP_6C | Sleep Module (7-day recall) | Between Week 1 and Week 2 | Symptom PGI-S | Symptom PGI-S change = 0 |
| TRTAP_6D | Sleep Module (7-day recall) | Between Week 1 and Week 3 | Symptom PGI-S | Symptom PGI-S change = 0 |

### Known-groups comparisons

The known-groups were defined as follows:

- COSP severity (clinician-rated)
  - Mild (reference)
  - Moderate
  - Severe
  - Very severe
- COSP severity (patient-reported)
  - Mild [pain score of 1-3 on a 0-10 scale, past week recall] (reference)
  - Moderate [pain score of 4-6 on a 0-10 scale, past week recall]
  - Severe [pain score of 7-10 on a 0-10 scale, past week recall]
  - No eye pain [pain score of 0 on a 0-10 scale]
