# Supplementary material for: Psychometric validation of the Chronic Ocular Pain Questionnaire (COP-Q)
Source: J Patient Rep Outcomes. 2025 Mar 12;9:32. doi: 10.1186/s41687-025-00862-9 (PMC11903982; doi:10.1186/s41687-025-00862-9)
Supplement: Supplementary file 5 — Supplementary Material 5 [file 41687_2025_862_MOESM5_ESM.docx]

## Supplementary 5. Demographic and clinical characteristics of the psychometric analysis population

Table 1. Demographic and clinical characteristics of the psychometric analysis population at baseline (N=124)

|  | COP-Q psychometric analysis population (N=124) |
| --- | --- |
| Participant sociodemographic characteristics | Statistic or N (%) |
| **Level of education** |  |
| College or Associate degree (vocational or academic) | 42 (33.9%) |
| Undergraduate degree (Bachelor's degree, Professional degree or equivalent) | 42 (33.9%) |
| Graduate degree (Masters, Doctorate or equivalent) | 20 (16.1%) |
| Middle and/or high school (Grade 6 to Grade 12; high school diploma/General Education Diploma or equivalent) | 20 (16.1%) |
| Work status | |
| Working full-time (in a paid job or as volunteer) | 59 (47.6%) |
| Retired | 42 (33.9%) |
| Working part-time (in a paid job or as volunteer) | 12 (9.7%) |
| Unemployed (for reasons unrelated to eye pain) | 5 (4.0%) |
| Full-time student | 2 (1.6%) |
| Disabled | 2 (1.6%) |
| Full-time homemaker | 1 (0.8%) |
| Part-time student | 1 (0.8%) |
| Health in general | |
| Good | 82 (66.1%) |
| Very good | 23 (18.5%) |
| Fair | 19 (15.3%) |
| COSP patient-reported severity level over past 7 days | |
| Severe (pain score 7-10) | 61 (49.2%) |
| Moderate (pain score 4-6) | 55 (44.4%) |
| Mild (pain score 1-3) | 8 (6.5%) |
| *Current eye pain treatment | |
| **Taking an eye pain treatment** | 93 (75.0%) |
| Eye Drops | 79 (55.6%) |
| Eye Gel | 20 (14.1%) |
| Artificial tears | 18 (12.7%) |
| Cold/hot compress | 11 (7.7%) |
| Pain relief tablets | 7 (4.9%) |
| Punctual Plugs | 2 (1.4%) |
| Epilepsy/migraine tablets | 1 (0.7%) |
| Antihistamine | 1 (0.7%) |
| Eye scrub | 1 (0.7%) |
| Thyroid medication | 1 (0.7%) |
| Vitamins and Minerals | 1 (0.7%) |
| **No eye pain treatment** | 31 (25.0%) |
| Non-ophthalmological co-morbid condition diagnosis | |
| None | 45 (36.3%) |
| Sjögren’s syndrome | 4 (3.2%) |
| Migraines | 4 (3.2%) |
| Diabetes | 3 (2.4%) |
| Systemic lupus erythematosus | 2 (1.6%) |
| Fibromyalgia | 2 (1.6%) |
| Thyroid disease | 1 (0.8%) |
| Severity of Patient experience of dry eye disease | |
| Moderate | 64 (51.6%) |
| Mild | 23 (18.5%) |
| Severe | 20 (16.1%) |
| Missing | 16 (12.9%) |
| Very Severe | 1 (0.8%) |
