# Supplementary material for: Psychometric validation of the Chronic Ocular Pain Questionnaire (COP-Q)
Source: J Patient Rep Outcomes. 2025 Mar 12;9:32. doi: 10.1186/s41687-025-00862-9 (PMC11903982; doi:10.1186/s41687-025-00862-9)
Supplement: Supplementary file 6 — Supplementary Material 6 [file 41687_2025_862_MOESM6_ESM.docx]

## Supplementary 6. Item response distributions

| Table 1. Item response distributions for the COP-Q Eye Pain Severity Module for the morning diary at Day 2 | | | | | | | | | | | | |
| --- | --- | --- | --- | --- | --- | --- | --- | --- | --- | --- | --- | --- |
|  | **COP-Q psychometric analysis population (N=124)^1^** | | | | | | | | | | | |
| **Eye Pain Severity Module Item** | **0** | **1** | **2** | **3** | **4** | **5** | **6** | **7** | **8** | **9** | **10** | **Missing** |
| Eye Pain Severity | 3 (5.7%) | 5 (9.4%) | 8 (15.1%) | 4 (7.5%) | 5 (9.4%) | 7 (13.2%) | 3 (5.7%) | 8 (15.1%) | 7 (13.2%) | 3 (5.7%) | 0 | 5 |
| ^1^Due to alternating study design, sample size is halved. | | | | | | | | | | | | |

Table 2. Item response distributions for the continuous response scale of COP-Q Eye Pain Severity Module for the evening diary at Day 2

|  | **COP-Q psychometric analysis population (N=124)** | | | | | | | | | | | |
| --- | --- | --- | --- | --- | --- | --- | --- | --- | --- | --- | --- | --- |
| **Eye Pain Severity Module Item** | **0** | **1** | **2** | **3** | **4** | **5** | **6** | **7** | **8** | **9** | **10** | **Missing** |
|  | | | | | | | | | | | | |
| Eye Pain Severity | 5 (8.9%) | 3 (5.4%) | 6 (10.7%) | 3 (5.4%) | 5 (8.9%) | 9 (16.1%) | 7 (12.5%) | 7 (12.5%) | 8 (14.3%) | 3 (5.4%) | 0 | 2 |
| ^1^Due to alternating study design, sample size is halved. | | | | | | | | | | | | |

Table 3. Item response distributions for the categorical response scale of COP-Q Eye Pain Frequency Module at Day 2

|  | **COP-Q psychometric analysis population (N=124)** | | | | | |
| --- | --- | --- | --- | --- | --- | --- |
| **Eye Pain Frequency Module Item** | **None of the time** | **A little of the time** | **Some of the time** | **A lot of the time** | **All of the time** | **Missing** |
| Eye Pain Frequency | 2 (1.7%) | 30 (25.9%) | 52 (44.8%) | 32 (27.6%) | 0 | 8 |


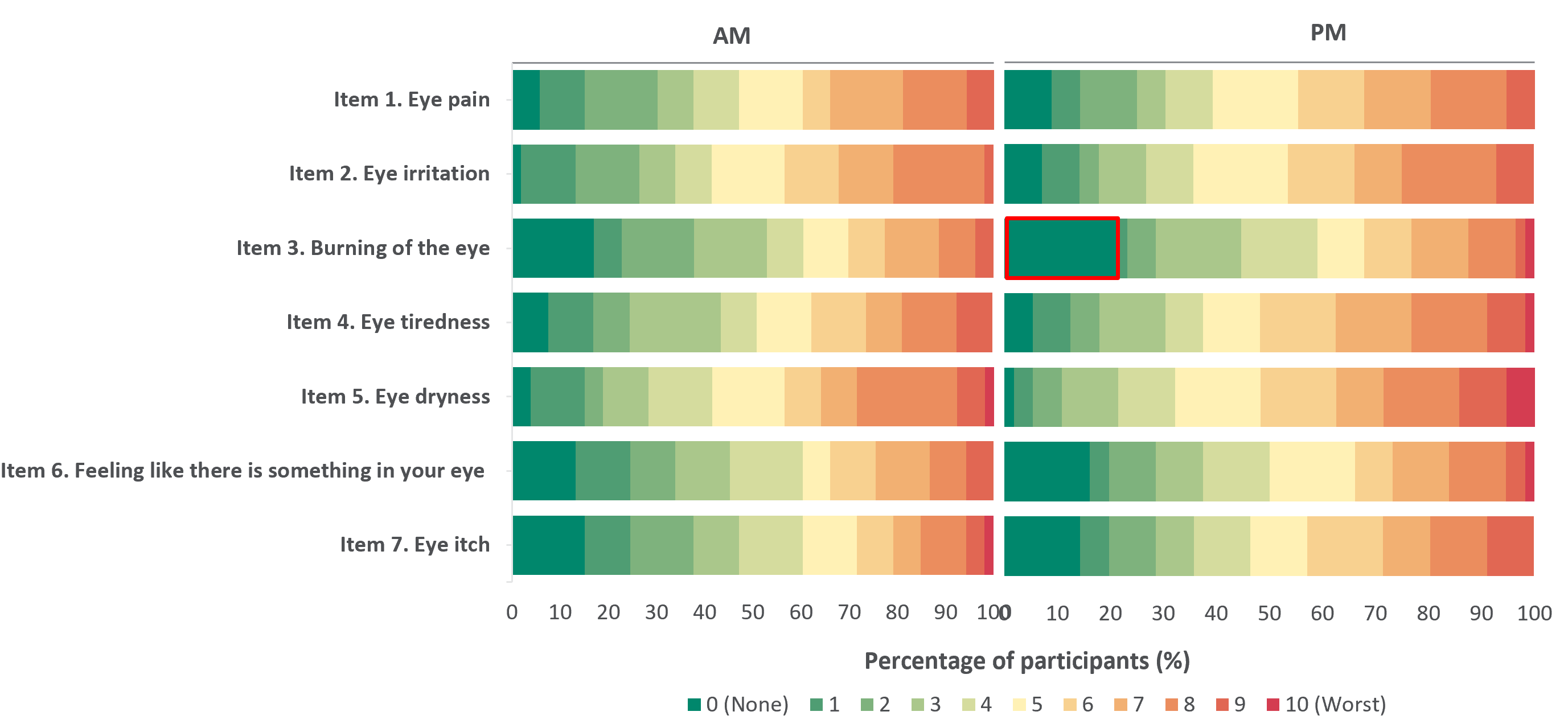


*To note: The red box in the graph above indicates an item (‘Burning of the eye’) with* ***>20%*** *of participants endorsing the ‘0 –No burning of the eye’ option which may indicate a ceiling effect for this item for the PM version of the 4-hour recall period Symptom Module.*

Figure 1. Item Response Distribution of the COP-Q Symptom Module (4-hour recall period, AM and PM diaries at Day 2).


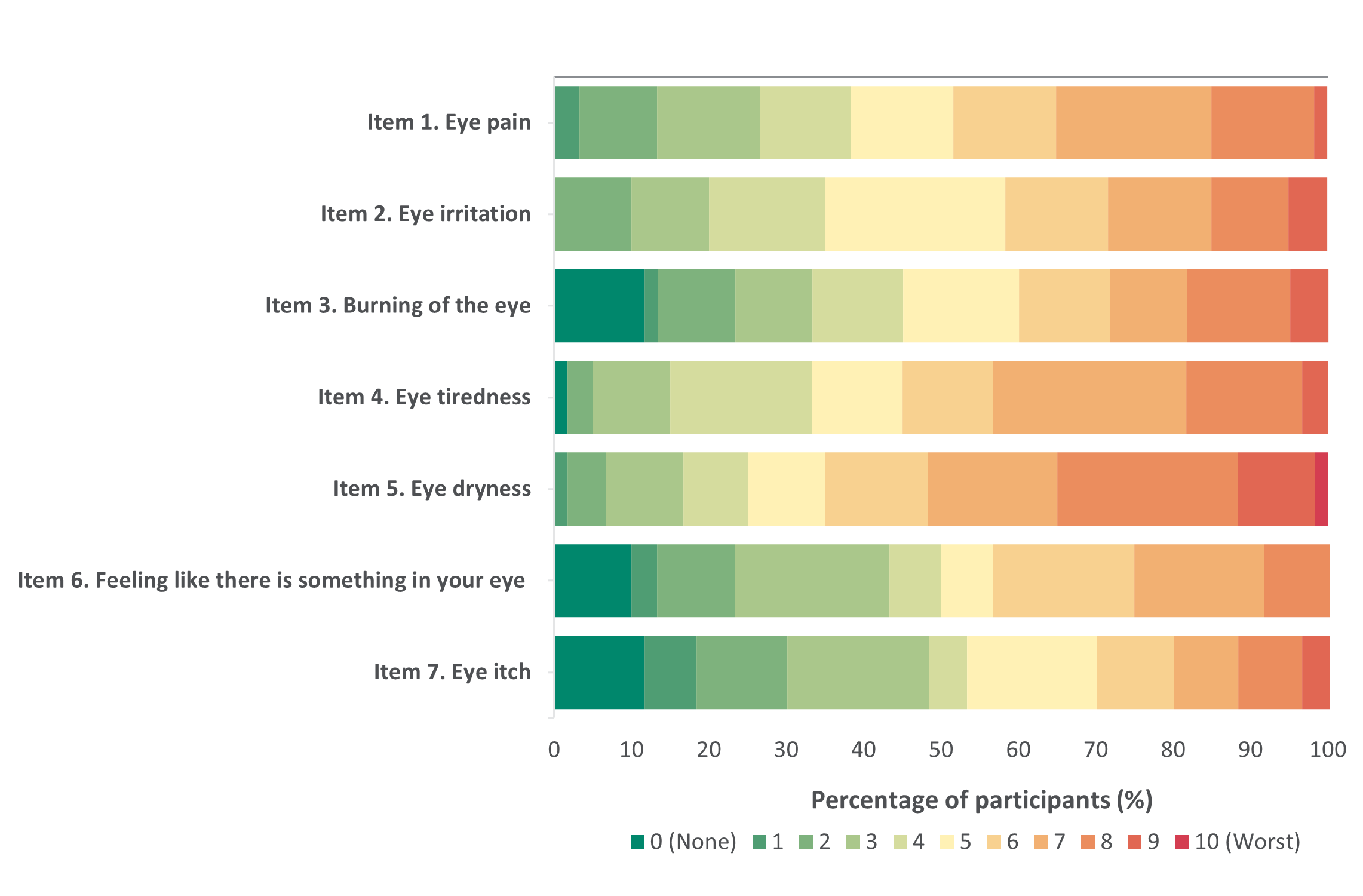


Figure 2. Item Response Distribution of the COP-Q Symptom Module (24-hour recall period at Day 2).


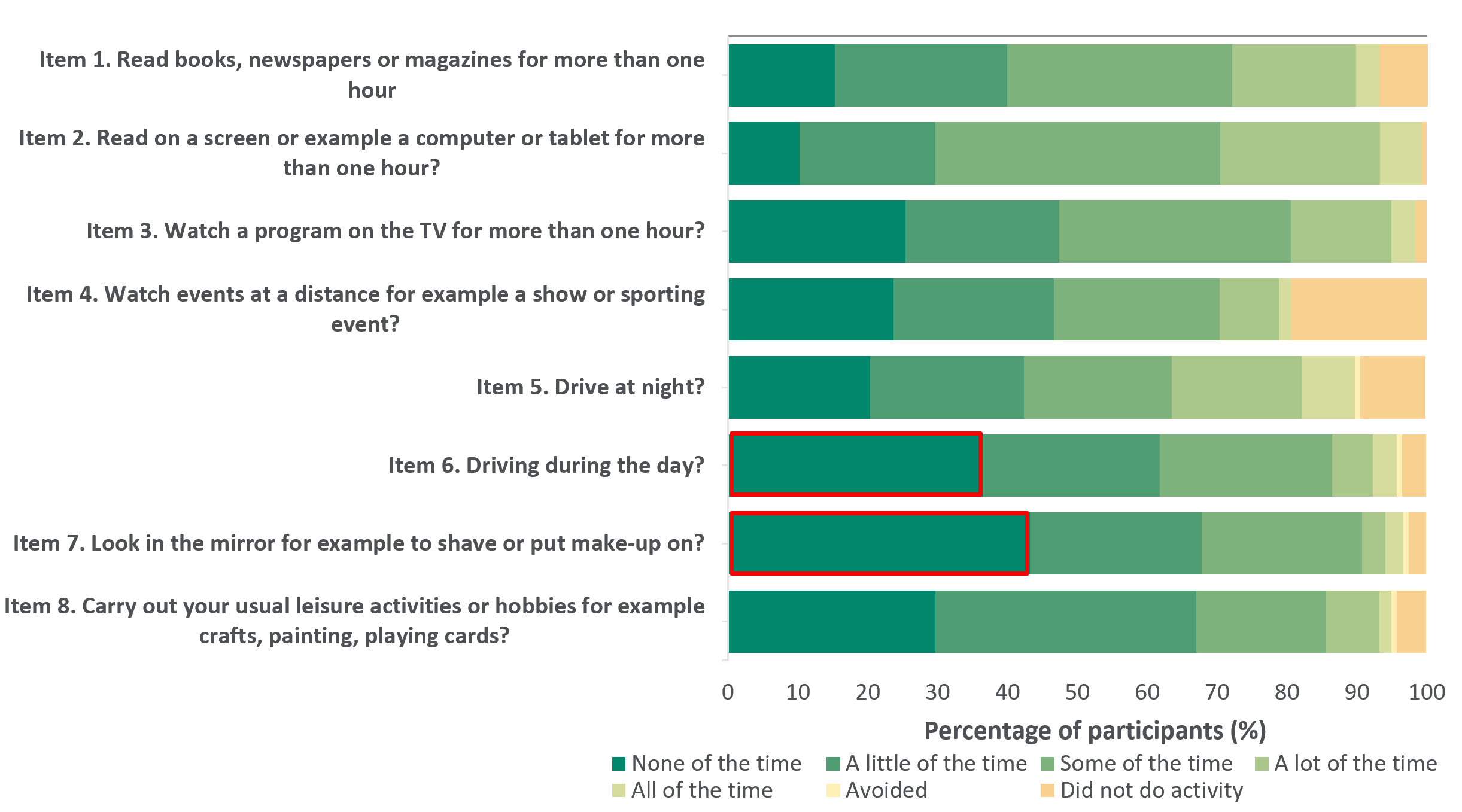


*To note: The red boxes in the graph above indicates two items (‘Driving during the day’ and ‘Look in the mirror for example to shave or put make-up on’) with* ***>30%*** *of participants endorsing the ‘None of the time’ options which may indicate a ceiling effect.*

Figure 3. Item Response Distribution of the COP-Q VTM at Week 2.


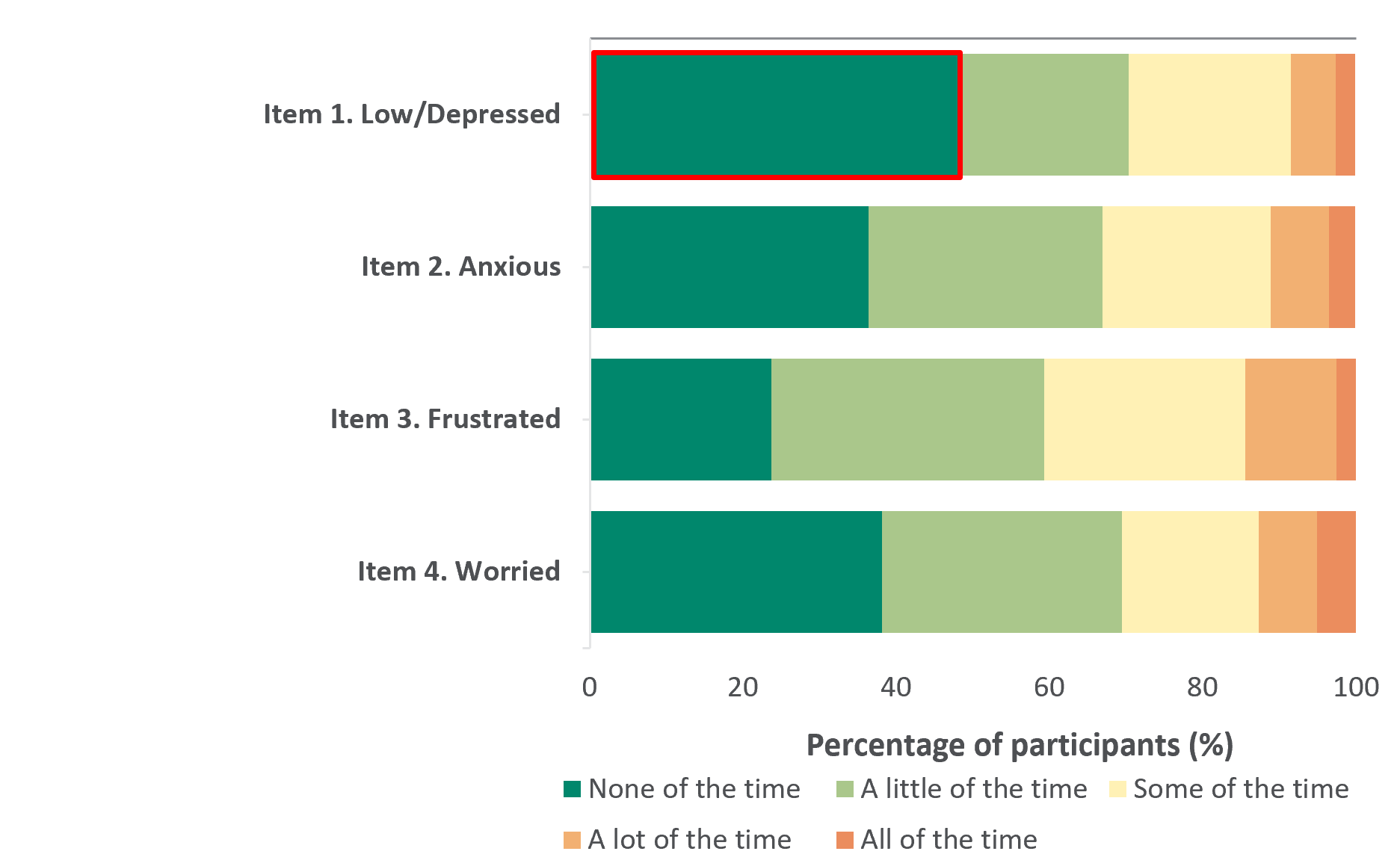


*To note: The red box in the graph indicates an item (‘Low/Depressed’) with* ***>40%*** *of participants endorsing the ‘None of the time’ option which may indicate a ceiling effect.*

Figure 4. Item Response Distribution of the COP-Q HRQoL Module at Week 2.

| **Table 4. Item response distributions for items for the Sleep Module at week 2** | | | | | | |
| --- | --- | --- | --- | --- | --- | --- |
|  | **COP-Q psychometric analysis population (N=124)** | | | | | |
| **Sleep Module Item** | **0 Nights** | **1-2 Nights** | **3-4 Nights** | **5-6 Nights** | **Every Night** | **Missing** |
| Item 5. Sleep affected | 46 (39.0%) | 37 (31.4%) | 22 (18.6%) | 8 (6.8%) | 5 (4.2%) | 6 |

| Uses responses: Level 1 = 0 nights, Level 2 = 1-2 nights, Level 3 = 3-4 nights, Level 4 = 5-6 nights and Level 5 = Every night. |
| --- |
