# Supplementary material for: Psychometric validation of the Chronic Ocular Pain Questionnaire (COP-Q)
Source: J Patient Rep Outcomes. 2025 Mar 12;9:32. doi: 10.1186/s41687-025-00862-9 (PMC11903982; doi:10.1186/s41687-025-00862-9)
Supplement: Supplementary file 7 — Supplementary Material 7 [file 41687_2025_862_MOESM7_ESM.docx]

## Supplementary 7. Inter-item correlations

| Table 1. Inter-item correlations for the COP-Q Symptom Module items in the full analysis population at the Day 2 morning administration (4-hour recall) | | | | | | | | | | | | | | |  |
| --- | --- | --- | --- | --- | --- | --- | --- | --- | --- | --- | --- | --- | --- | --- | --- |
|  | **Pearson Correlation Coefficient** | | | | | | | | | | | | | |  |
| **COP-Q Symptom Items** | **1** | | **2** | | **3** | | **4** | | **5** | | **6** | | **7** | |  |
| Item 1. Eye pain | 1.000 | |  | |  | |  | |  | |  | |  | |  |
| Item 2. Eye irritation | 0.867 | | 1.000 | |  | |  | |  | |  | |  | |  |
| Item 3. Burning of the eye | 0.790 | | 0.853 | | 1.000 | |  | |  | |  | |  | |  |
| Item 4. Eye tiredness | 0.680 | | 0.761 | | 0.669 | | 1.000 | |  | |  | |  | |  |
| Item 5. Eye dryness | 0.772 | | 0.873 | | 0.701 | | 0.804 | | 1.000 | |  | |  | |  |
| Item 6. Feeling like there is something in your eye | 0.685 | | 0.770 | | 0.700 | | 0.535 | | 0.734 | | 1.000 | |  | |  |
| Item 7. Eye itch | 0.589 | | 0.729 | | 0.753 | | 0.640 | | 0.665 | | 0.746 | | 1.000 | |  |
| **Table 2. Inter-item correlations for the COP-Q Symptom Module items in the full analysis population at the Day 2 evening administration (4-hour recall)** | | | | | | | | | | | | | | | |
|  | | **Pearson Correlation Coefficient** | | | | | | | | | | | | | |
| **COP-Q Symptom Items** | | **1** | | **2** | | **3** | | **4** | | **5** | | **6** | | **7** | |
| Item 1. Eye pain | | 1.000 | |  | |  | |  | |  | |  | |  | |
| Item 2. Eye irritation | | 0.874 | | 1.000 | |  | |  | |  | |  | |  | |
| Item 3. Burning of the eye | | 0.693 | | 0.747 | | 1.000 | |  | |  | |  | |  | |
| Item 4. Eye tiredness | | 0.790 | | 0.798 | | 0.629 | | 1.000 | |  | |  | |  | |
| Item 5. Eye dryness | | 0.810 | | 0.860 | | 0.690 | | 0.775 | | 1.000 | |  | |  | |
| Item 6. Feeling like there is something in your eye | | 0.749 | | 0.790 | | 0.786 | | 0.617 | | 0.743 | | 1.000 | |  | |
| Item 7. Eye itch | | 0.739 | | 0.797 | | 0.787 | | 0.651 | | 0.670 | | 0.756 | | 1.000 | |

| **Table 3. Inter-item correlations for the COP-Q Symptom Module items in the full analysis population for the 24-hour recall at Day 2** | | | | | | | |
| --- | --- | --- | --- | --- | --- | --- | --- |
|  | **Pearson Correlation Coefficient** | | | | | | |
| **COP-Q Symptom Items** | **1** | **2** | **3** | **4** | **5** | **6** | **7** |
| Item 1. Eye pain | 1.000 |  |  |  |  |  |  |
| Item 2. Eye irritation | 0.800 | 1.000 |  |  |  |  |  |
| Item 3. Burning of the eye | 0.663 | 0.762 | 1.000 |  |  |  |  |
| Item 4. Eye tiredness | 0.672 | 0.743 | 0.572 | 1.000 |  |  |  |
| Item 5. Eye dryness | 0.701 | 0.765 | 0.596 | 0.788 | 1.000 |  |  |
| Item 6. Feeling like there is something in your eye | 0.480 | 0.572 | 0.665 | 0.445 | 0.518 | 1.000 |  |
| Item 7. Eye itch | 0.541 | 0.552 | 0.701 | 0.459 | 0.449 | 0.815 | 1.000 |

| **Table 4. Inter-item correlations for the COP-Q VTM items in the full analysis population at Week 2** | | | | | | | | |
| --- | --- | --- | --- | --- | --- | --- | --- | --- |
|  | **Polychoric Correlation Coefficient** | | | | | | | |
| **VTM Items** | **1** | **2** | **3** | **4** | **5** | **6** | **7** | **8** |
| Item 1. Read books, newspapers or magazines for more than one hour? | 1.000 |  |  |  |  |  |  |  |
| Item 2. Read on a screen or example a computer or tablet for more than one hour? | 0.736 | 1.000 |  |  |  |  |  |  |
| Item 3. Watch a program on the TV for more than one hour? | 0.749 | 0.775 | 1.000 |  |  |  |  |  |
| Item 4. Watch events at a distance for example a show or sporting event? | 0.736 | 0.610 | 0.757 | 1.000 |  |  |  |  |
| Item 5. Drive at night? | 0.562 | 0.443 | 0.562 | 0.683 | 1.000 |  |  |  |
| Item 6. Driving during the day? | 0.625 | 0.669 | 0.709 | 0.713 | 0.722 | 1.000 |  |  |
| Item 7. Look in the mirror for example to shave or put make-up on? | 0.591 | 0.535 | 0.699 | 0.616 | 0.615 | 0.733 | 1.000 |  |
| Item 8. Carry out your usual leisure activities or hobbies for example crafts, painting, playing cards? | 0.643 | 0.582 | 0.686 | 0.741 | 0.649 | 0.741 | 0.809 | 1.000 |

| **Table 5. Inter-item correlations for the COP-Q Health Related Quality of Life Module items in the psychometric analysis sample at Week 2** | | | | |
| --- | --- | --- | --- | --- |
|  | **Polychoric Correlation Coefficient** | | | |
| **HRQoL Items** | **1** | **2** | **3** | **4** |
| Item 1. Low/Depressed | 1.000 |  |  |  |
| Item 2. Anxious | 0.845 | 1.000 |  |  |
| Item 3. Frustrated | 0.814 | 0.806 | 1.000 |  |
| Item 4. Worried | 0.834 | 0.865 | 0.867 | 1.000 |
