# Supplementary material for: Psychometric validation of the Chronic Ocular Pain Questionnaire (COP-Q)
Source: J Patient Rep Outcomes. 2025 Mar 12;9:32. doi: 10.1186/s41687-025-00862-9 (PMC11903982; doi:10.1186/s41687-025-00862-9)
Supplement: Supplementary file 8 — Supplementary Material 8 [file 41687_2025_862_MOESM8_ESM.docx]

## Supplementary 8. Unconstrained CFA path diagrams for the Symptom Modules (4-hr AM, 4-hr PM and 24-hr), VTM and HRQoL


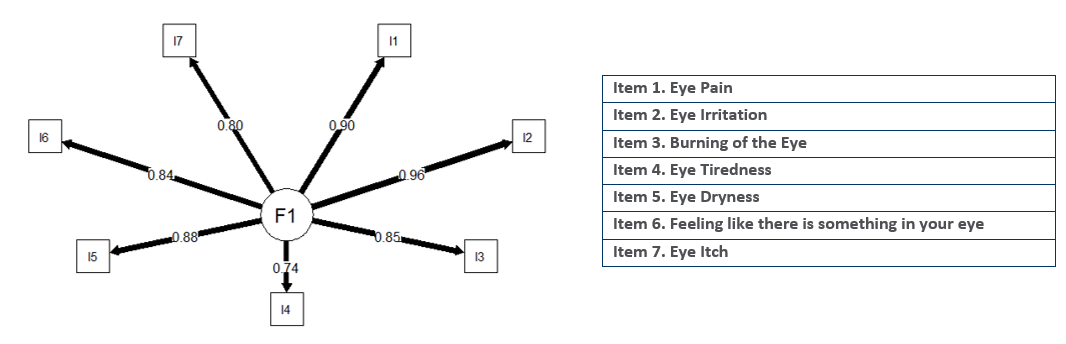


Figure 1. Four-hour AM path diagram; Model 1


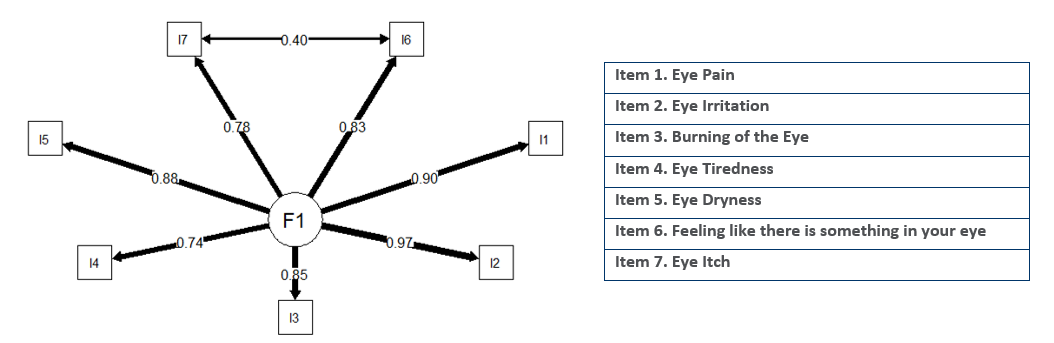


**Figure 2. Four-hour AM path diagram; Model 2 (Error term specified)**


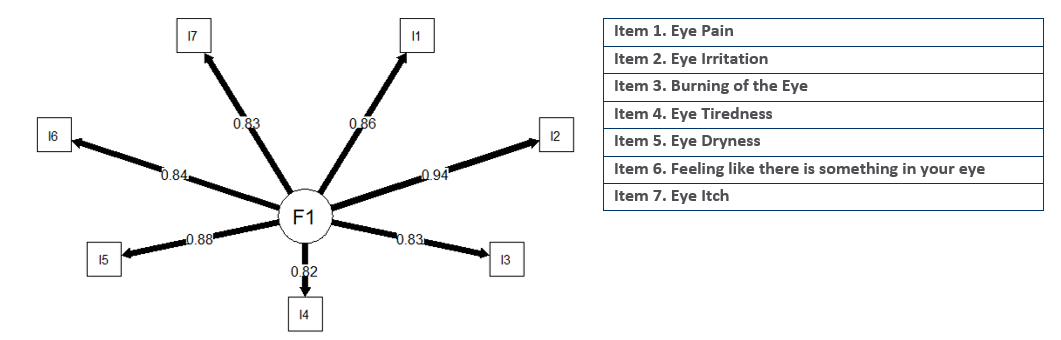


Figure 3. Four-hour PM path diagram; Model 1


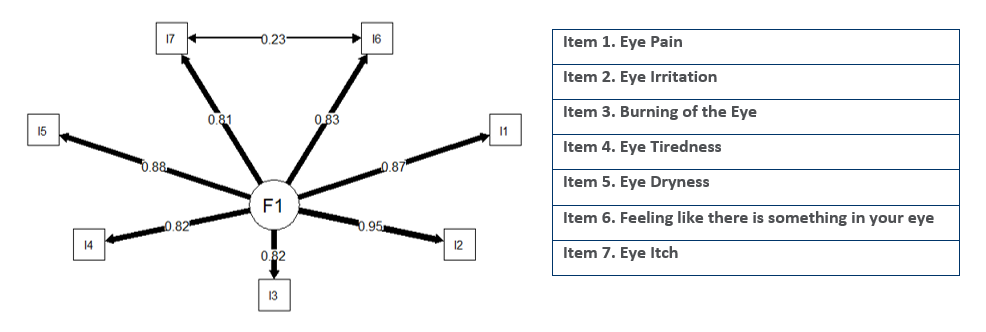


Figure 4. Four-hour PM path diagram; Model 2 (Error term specified)


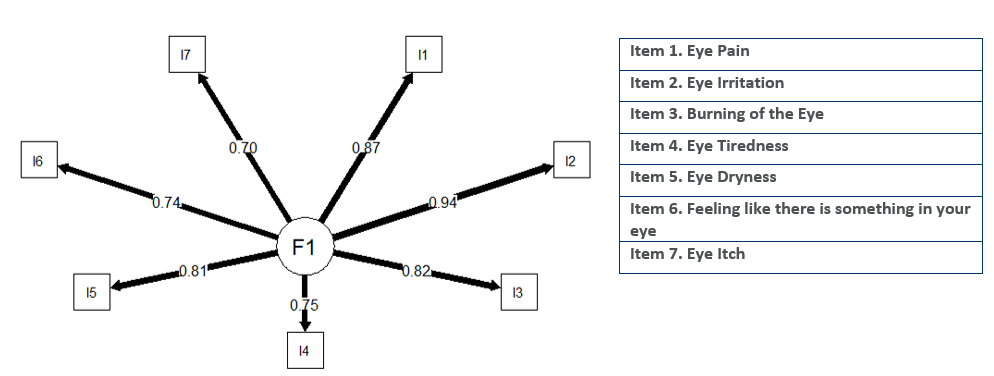


Figure 5. Twenty-four – hour path diagram; Model 1


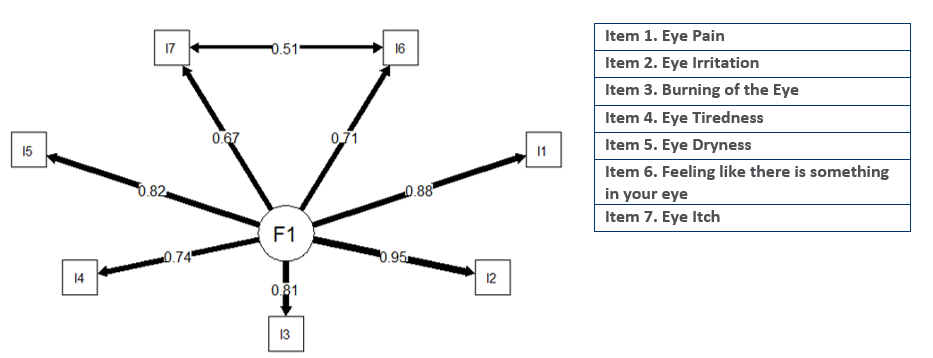


Figure 6. Twenty-four – hour path diagram; Model 2 (Error term specified)


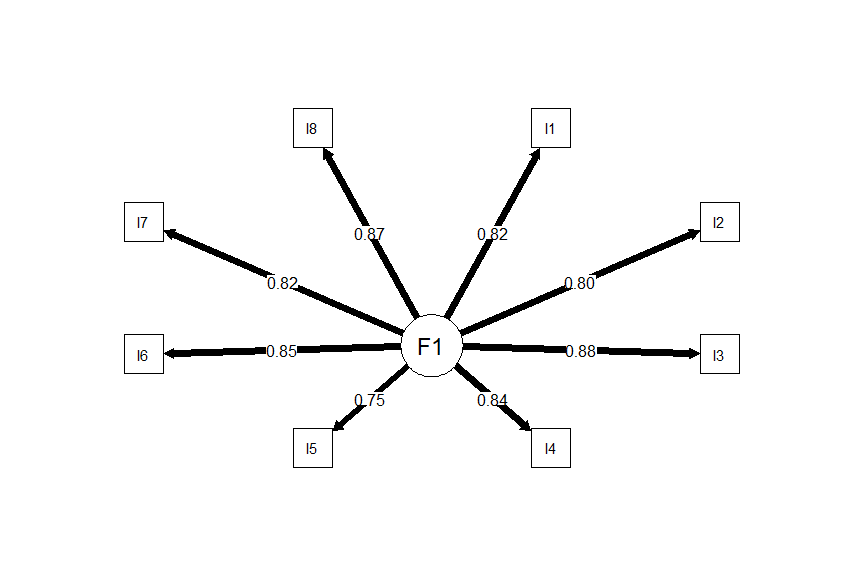


| **Item 1. Read books, newspapers or magazines for more than one hour?** |
| --- |
| **Item 2. Read on a screen or example a computer or tablet for more than one hour?** |
| **Item 3. Watch a program on the TV for more than one hour?** |
| **Item 4. Watch events at a distance for example a show or sporting event?** |
| **Item 5. Drive at night?** |
| **Item 6. Driving during the day?** |
| **Item 7. Look in the mirror for example to shave or put make-up on?** |
| **Item 8. Carry out your usual leisure activities or hobbies for example crafts, painting, playing cards?** |

**Figure 7. VTM path diagram prior to item deletion**


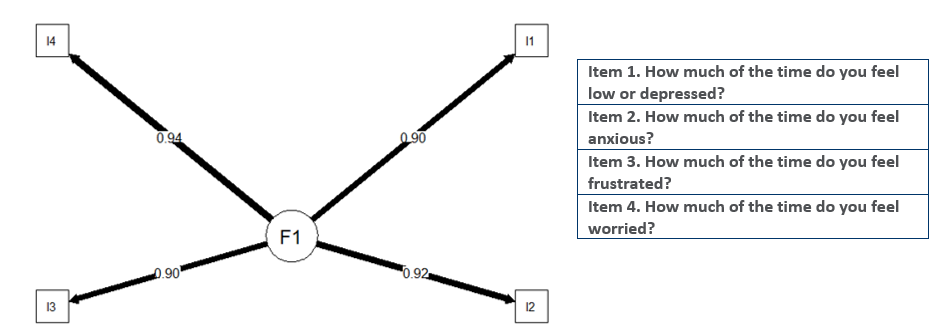


Figure 8. HRQoL path diagram
