# Supplementary material for: Psychometric validation of the Chronic Ocular Pain Questionnaire (COP-Q)
Source: J Patient Rep Outcomes. 2025 Mar 12;9:32. doi: 10.1186/s41687-025-00862-9 (PMC11903982; doi:10.1186/s41687-025-00862-9)
Supplement: Supplementary file 9 — Supplementary Material 9 [file 41687_2025_862_MOESM9_ESM.docx]

## Supplementary 9. Infit and outfit statistics

| Table 1. Infit and outfit statistics for the VTM and HRQoL items at Week 2 | | |
| --- | --- | --- |
| **Module (Items)** | **Outfit** | **Infit** |
| **VTM** | | |
| Item 1. Read books | 0.757 | 0.732 |
| Item 2. Read on a screen | 0.819 | 0.810 |
| Item 3. Watch TV | 0.691 | 0.727 |
| Item 4. Watch events | 0.733 | 0.586 |
| Item 5. Drive at night? | 1.234 | 1.161 |
| Item 6. Driving during the day? | 0.740 | 0.783 |
| Item 7. Look in the mirror | 0.881 | 0.925 |
| Item 8. Leisure activities or hobbies | 0.695 | 0.617 |
| **HRQoL** | | |
| Item 1. Low/Depressed | 0.620 | 0.827 |
| Item 2. Anxious | 0.548 | 0.603 |
| Item 3. Frustrated | 0.547 | 0.578 |
| Item 4. Worried | 0.554 | 0.650 |
| Note. Infit and outfit values cut-offs outlined below: | | |
| >2.0: Distorts or degrades the measurement system. May be caused by only one or two observations.  1.5-2.0: Unproductive for construction of measurement, but not degrading.  0.5-1.5: Productive for measurement.  <0.5: Less productive for measurement, but not degrading. May produce misleadingly high reliability and separation coefficients. | | |
