# Supplementary material for: Psychometric validation of the Chronic Ocular Pain Questionnaire (COP-Q)
Source: J Patient Rep Outcomes. 2025 Mar 12;9:32. doi: 10.1186/s41687-025-00862-9 (PMC11903982; doi:10.1186/s41687-025-00862-9)
Supplement: Supplementary file 10 — Supplementary Material 10 [file 41687_2025_862_MOESM10_ESM.docx]

## Supplementary 10. Rating Scale Model Parameters for the VTM and HRQoL Module

Table 1. Rating Scale Model Parameters for the VTM

| **Visual Tasking Rating Scale Model Parameters** | | | | | | |
| --- | --- | --- | --- | --- | --- | --- |
|  | **a1** | **b1** | **b2** | **b3** | **b4** | **c** |
| VTM | | | | | | |
| Item 7. Look in the mirror | 1 | -1.68 | -0.51 | 1.57 | 3.34 | -1.55 |
| Item 6. Driving during the day? | 1 | -1.68 | -0.51 | 1.57 | 3.34 | -1.19 |
| Item 8. Leisure activities or hobbies | 1 | -1.68 | -0.51 | 1.57 | 3.34 | -1.18 |
| Item 4. Watch events | 1 | -1.68 | -0.51 | 1.57 | 3.34 | -0.71 |
| Item 3. Watch TV | 1 | -1.68 | -0.51 | 1.57 | 3.34 | -0.40 |
| Item 1. Read books | 1 | -1.68 | -0.51 | 1.57 | 3.34 | 0.00 |
| Item 5. Drive at night? | 1 | -1.68 | -0.51 | 1.57 | 3.34 | 0.01 |
| Item 2. Read on a screen | 1 | -1.68 | -0.51 | 1.57 | 3.34 | 0.55 |
| a1 = Slopes. b1 to b3 = Intercepts/thresholds. c = item difficulty. | | | | | | |

Table 2. Rating Scale Model Parameters for the HRQoL Module

| **HRQoL Rating Scale Model Parameters** | | | | | | |
| --- | --- | --- | --- | --- | --- | --- |
|  | **a1** | **b1** | **b2** | **b3** | **b4** | **c** |
| HRQoL | | | | | | |
| Item 1. Low/Depressed | 1 | -0.40 | 1.76 | 4.17 | 5.62 | 0.00 |
| Item 4. Worried | 1 | -0.40 | 1.76 | 4.17 | 5.62 | 0.55 |
| Item 2. Anxious | 1 | -0.40 | 1.76 | 4.17 | 5.62 | 0.58 |
| Item 3. Frustrated | 1 | -0.40 | 1.76 | 4.17 | 5.62 | 1.22 |
| a1 = Slopes. b1 to b3 = Intercepts/thresholds. c = item difficulty. | | | | | | |
