# Supplementary material for: Psychometric validation of the Chronic Ocular Pain Questionnaire (COP-Q)
Source: J Patient Rep Outcomes. 2025 Mar 12;9:32. doi: 10.1186/s41687-025-00862-9 (PMC11903982; doi:10.1186/s41687-025-00862-9)
Supplement: Supplementary file 11 — Supplementary Material 11 [file 41687_2025_862_MOESM11_ESM.docx]

## Supplementary 11. Item Characteristic Curves and Item Information Curves for the VTM and HRQoL Module at Week 2


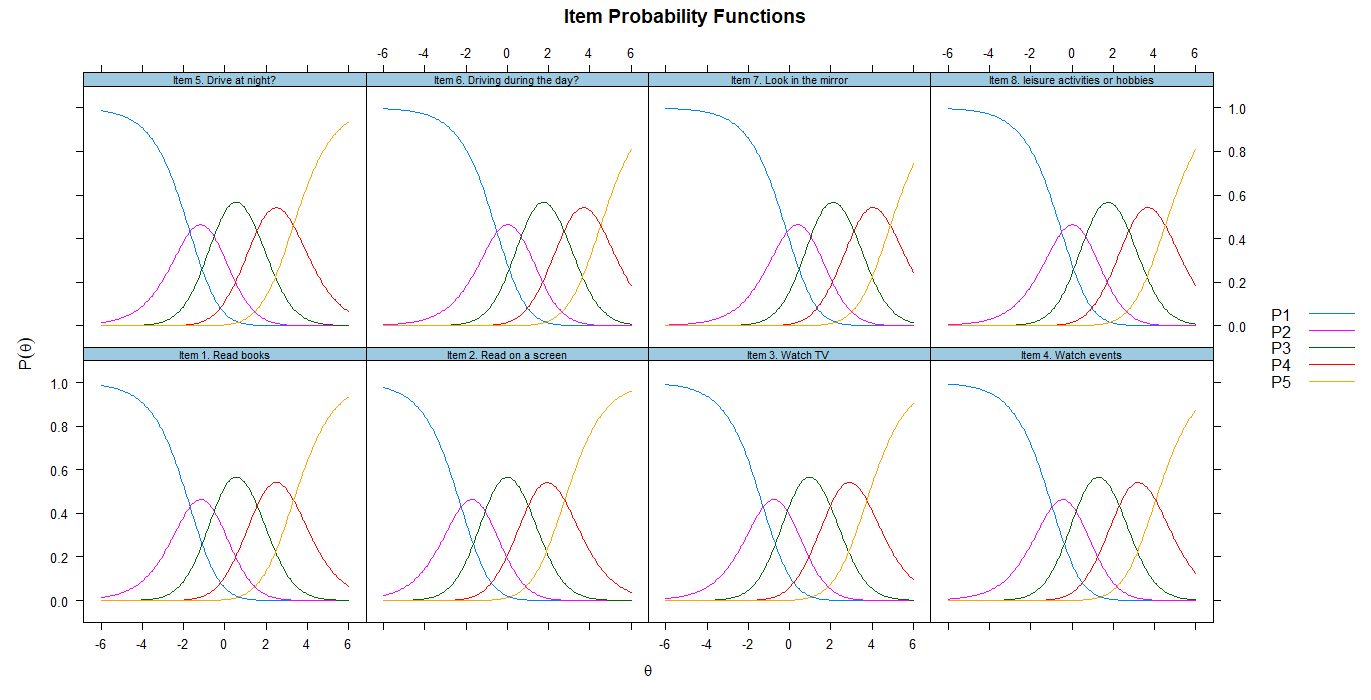

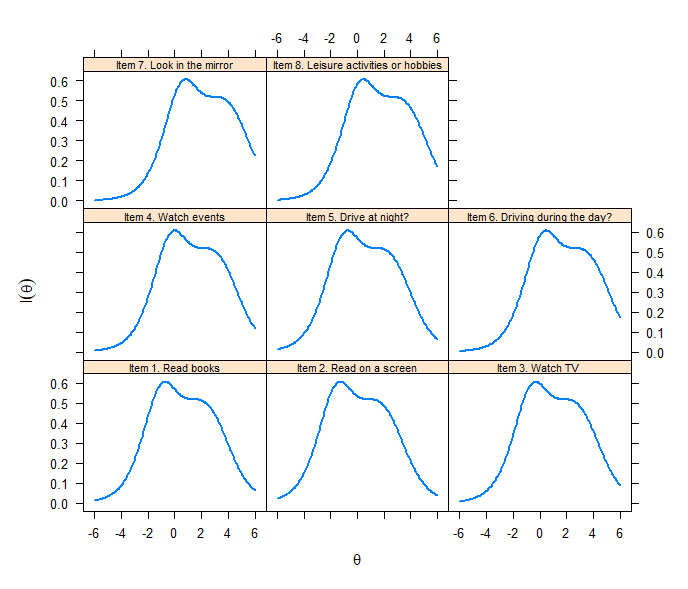


Figure 1. VTM item characteristic curves

Figure 2. VTM item information curves

Figure 1. VTM item characteristic curves


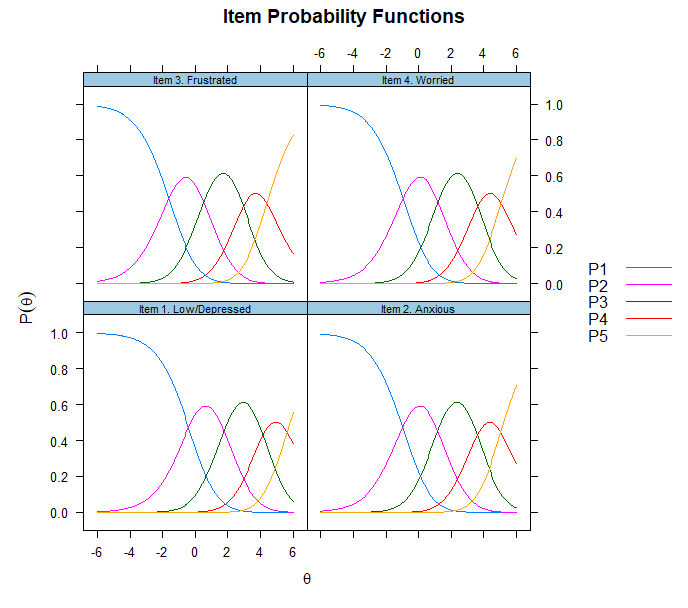


Figure 3. HRQoL Module item characteristic curves


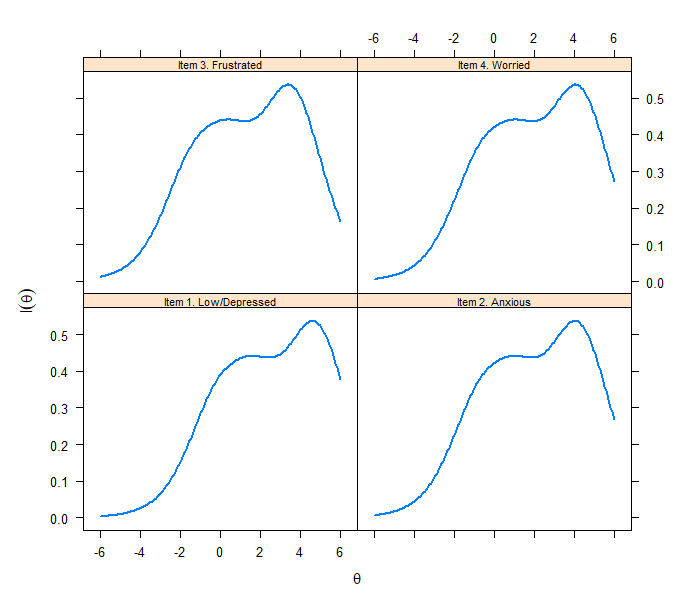


Figure 4. HRQoL Module item information curves
