# Supplementary material for: Psychometric validation of the Chronic Ocular Pain Questionnaire (COP-Q)
Source: J Patient Rep Outcomes. 2025 Mar 12;9:32. doi: 10.1186/s41687-025-00862-9 (PMC11903982; doi:10.1186/s41687-025-00862-9)
Supplement: Supplementary file 12 — Supplementary Material 12 [file 41687_2025_862_MOESM12_ESM.docx]

## Supplementary 12. Item-person maps

##
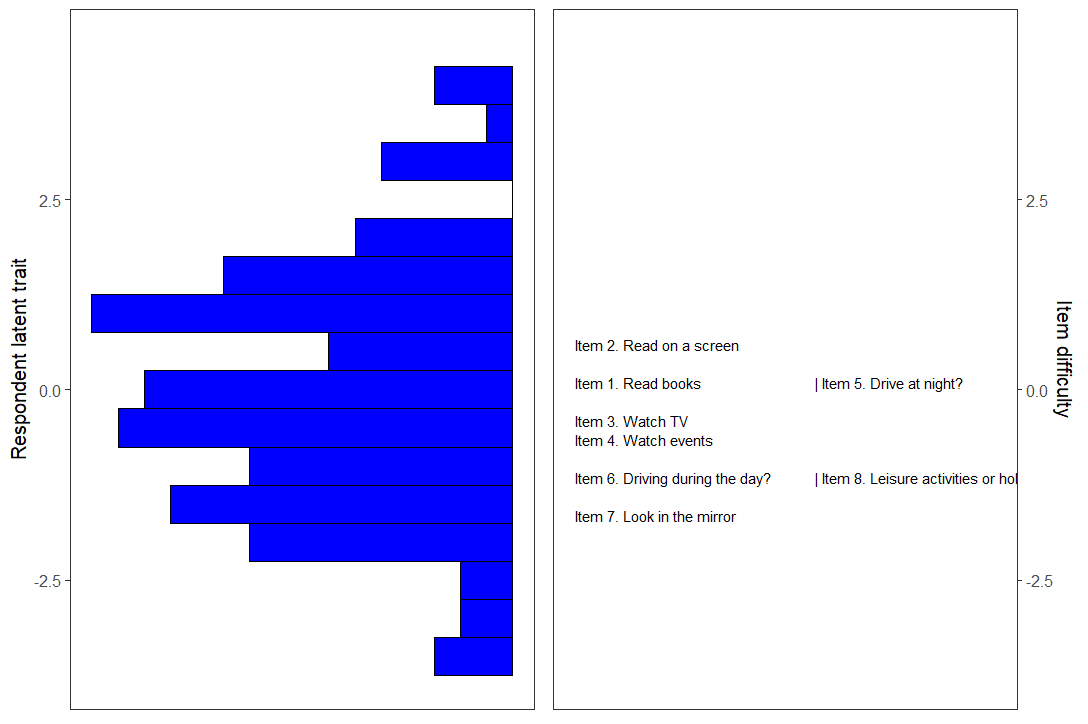


Figure 1. Item-person map representing participants and VTM items on the same latent trait at Week 2

##
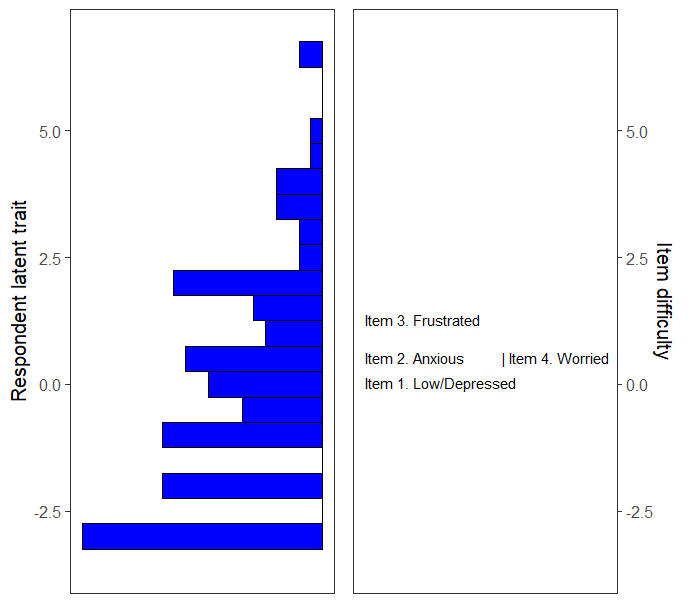


Figure 2. Item-person map representing participants and HRQoL items on the same latent trait at Week 2
