# Supplementary material for: Psychometric validation of the Chronic Ocular Pain Questionnaire (COP-Q)
Source: J Patient Rep Outcomes. 2025 Mar 12;9:32. doi: 10.1186/s41687-025-00862-9 (PMC11903982; doi:10.1186/s41687-025-00862-9)
Supplement: Supplementary file 13 — Supplementary Material 13 [file 41687_2025_862_MOESM13_ESM.docx]

## Supplementary File 13. Local dependency of items for the VTM and HRQoL Module

| **Table 1. Local Dependency of items for the COP-Q Visual Tasking Module (VTM)** | | | | | | | | |
| --- | --- | --- | --- | --- | --- | --- | --- | --- |
|  | **Item 1. Read books** | **Item 2. Read on a screen** | **Item 3. Watch TV** | **Item 4. Watch events** | **Item 5. Drive at night?** | **Item 6. Driving during the day?** | **Item 7. Look in the mirror** | **Item 8. Leisure activities or hobbies** |
| Item 1. Read books | 1.000 |  |  |  |  |  |  |  |
| Item 2. Read on a screen | 0.145 | 1.000 |  |  |  |  |  |  |
| Item 3. Watch TV | -0.035 | 0.148 | 1.000 |  |  |  |  |  |
| Item 4. Watch events | -0.034 | -0.314 | -0.033 | 1.000 |  |  |  |  |
| Item 5. Drive at night? | -0.249 | -0.422 | -0.373 | 0.010 | 1.000 |  |  |  |
| Item 6. Driving during the day? | -0.369 | -0.131 | -0.230 | -0.218 | 0.089 | 1.000 |  |  |
| Item 7. Look in the mirror | -0.282 | -0.297 | -0.094 | -0.290 | -0.166 | -0.007 | 1.000 |  |
| Item 8. Leisure activities or hobbies | -0.211 | -0.274 | -0.269 | -0.015 | -0.105 | -0.096 | **0.162** | 1.000 |
| Average residual correlation = -0.141, Yens Q3 cut off = 0.159 (Average residual correlation +0.30); **Bold** represents items that exceeded the cut off. | | | | | | | | |

| **Table 2. Local Dependency of items for the COP-Q Health-Related Quality of Life (HRQoL) Module** | | | | |
| --- | --- | --- | --- | --- |
|  | **Item 1.**  **Low/Depressed** | **Item 2. Anxious** | **Item 3.**  **Frustrated** | **Item 4.**  **Worried** |
| Item 1. Low/Depressed | 1.000 |  |  |  |
| Item 2. Anxious | -0.188 | 1.000 |  |  |
| Item 3. Frustrated | -0.361 | -0.402 | 1.000 |  |
| Item 4. Worried | -0.337 | -0.190 | -0.194 | 1.000 |
| Average residual correlation = -0.279, Yens Q3 cut off = 0.021 (Average residual correlation +0.30). | | | | |
