# Supplementary material for: Psychometric validation of the Chronic Ocular Pain Questionnaire (COP-Q)
Source: J Patient Rep Outcomes. 2025 Mar 12;9:32. doi: 10.1186/s41687-025-00862-9 (PMC11903982; doi:10.1186/s41687-025-00862-9)
Supplement: Supplementary file 14 — Supplementary Material 14 [file 41687_2025_862_MOESM14_ESM.docx]

## Supplementary 14. Rationale for item reduction

Table 1. Rationale for possible items for deletion

| Item | Rationale | | | | Decision |
| --- | --- | --- | --- | --- | --- |
|  | Inter-item correlation | Confirmatory Factor Analysis | Item properties | IRT modelling |  |
| 7 (Symptom module; ALL). ‘Please rate the severity of your eye itch at its worst over the past 4/24 hours’. | Inter-item correlations were less than 0.9. Lower correlations compared to other items. | Fit indices for all Symptom Modules suggest fit of the model was fair but not ideal.  Residual correlation with Item 6 (‘Something in your eye’) for Symptom Modules (0.085 – 0.23).  Lowest Factor loading. | NA | NA | Retain item due to clinical importance. |
| 7 (VTM). ‘Look in the mirror for example to shave or put your make-up on’. | Inter-item correlations were less than 0.9. | Residual correlation (0.127) present with Item 2 (‘Read on a screen for example a computer or a tablet for more than one hour’).  One of the lowest Factor loadings. | Ceiling effect for response option ‘None of the time’ (43.2%). | Potential local dependency with Item 8 (‘Carry out usual leisure activities or hobbies for example crafts, painting, playing cards’). | Item removal agreed. |
| 5 (VTM). ‘Drive at night’. | Inter-item correlations were less than 0.9. Lower correlations compared to other items. | Fit indices suggest that the model fits the data moderately well with fit improving with this item removed.  Residual correlations between Item 4 (‘Watch events at a distance for example a show or sporting event’) and Item 5 (‘Drive at night’) after Item 7 (‘Look in the mirror for example to shave or put your make-up on’) was removed.  Lowest Factor loading. | Approaching a ceiling effect but not currently over the threshold. | Performance is generally adequate for IRT. | Item removal agreed. |
| 3 (Symptom module 4-hour PM). ‘Please rate severity of any burning feelings in your eye(s) at its worst over the past 4 hours’. | Inter-item correlations were less than 0.9. | Factor loading above 0.4 and no residual correlation present. | Ceiling effect for response option ‘None of the time’ (21.4%). | NA | Retain as ceiling effect was only marginally over the threshold of >20% and other psychometric assessments are adequate. |
| 6 (VTM). ‘Drive during the day’. | Inter-item correlations were less than 0.9. | Factor loading above 0.4 and no residual correlation present. | Ceiling effect for response option ‘None of the time’ (36.4%). | Good performance across IRT analyses. | Retain as ceiling effect was only 6.4% over the threshold and other psychometric evaluations are adequate. Potential utility in the assessment of visual limitations with regard to day driving. |
| 1 (HRQoL). ‘How much of the time do you feel low or depressed.’ | Inter-item correlations were less than 0.9. | Factor loading above 0.4 and no residual correlation present. | Ceiling effect for response option ‘None of the time’ (48.3%). | Good performance across IRT analyses. | Retain. The ceiling effect is of some concern but the item presents as an important one for the assessment of HRQoL concerns. |
