# Supplementary material for: Psychometric validation of the Chronic Ocular Pain Questionnaire (COP-Q)
Source: J Patient Rep Outcomes. 2025 Mar 12;9:32. doi: 10.1186/s41687-025-00862-9 (PMC11903982; doi:10.1186/s41687-025-00862-9)
Supplement: Supplementary file 15 — Supplementary Material 15 [file 41687_2025_862_MOESM15_ESM.docx]

## Supplementary 15. Reliability and SEM for different number of missing items to form a daily score for each COP-Q Module

Table 1. Reliability and SEM for different number of missing items to form a daily score for each COP-Q Module

|  | | | | | | | | | | | | | | | | |
| --- | --- | --- | --- | --- | --- | --- | --- | --- | --- | --- | --- | --- | --- | --- | --- | --- |
|  | **HRQoL Module** | | **VTM** | | **Symptom Module 4hr; AM** | | **Symptom Module 4hr; AM minus Item 7** | | **Symptom Module 4hr; PM** | | **Symptom Module 4hr; PM minus Item 7** | | **Symptom Module 24hr** | | **Symptom Module 24hr; minus Item 7** | |
| **Items Missing** | **Reliability** | **SEM** | **Reliability** | **SEM** | **Reliability** | **SEM** | **Reliability** | **SEM** | **Reliability** | **SEM** | **Reliability** | **SEM** | **Reliability** | **SEM** | **Reliability** | **SEM** |
| **0** | 0.927 | 1.07 | 0.904 | 1.55 | 0.947 | 3.84 | 0.943 | 3.46 | 0.947 | 3.7 | 0.945 | 3.25 | 0.938 | 3.92 | 0.936 | 3.42 |
| **1** | 0.905 | 1.22 | 0.887 | 1.69 | 0.939 | 4.13 | 0.932 | 3.77 | 0.939 | 3.98 | 0.935 | 3.54 | 0.928 | 4.21 | 0.924 | 3.72 |
| **2** | 0.864 | 1.46 | 0.863 | 1.86 | 0.927 | 4.49 | 0.917 | 4.18 | 0.927 | 4.33 | 0.920 | 3.93 | 0.915 | 4.58 | 0.907 | 4.12 |
| **3** | 0.76 | 1.94 | 0.825 | 2.1 | 0.911 | 4.98 | 0.892 | 4.76 | 0.911 | 4.80 | 0.896 | 4.47 | 0.896 | 5.06 | 0.880 | 4.69 |
| **4** | - | - | 0.758 | 2.46 | 0.884 | 5.67 | 0.846 | 5.67 | 0.884 | 5.46 | 0.851 | 5.34 | 0.866 | 5.75 | 0.830 | 5.58 |
| **5** | - | - | 0.611 | 3.13 | 0.836 | 6.75 | 0.734 | 7.47 | 0.836 | 6.50 | 0.741 | 7.05 | 0.812 | 6.82 | 0.709 | 7.29 |
| **6** | - | - | - | - | 0.719 | 8.85 | - | - | 0.719 | 8.53 | - | - | 0.684 | 8.84 | - | - |
| **7** | - | - | - | - | - | - | - | - | - | - | - | - | - | - | - | - |
| **8** | - | - | - | - | - | - | - | - | - | - | - | - | - | - | - | - |
|  | | | | | | | | | | | | | | | | |

| **Table 2. Reliability for different number of missing days to form a 7-day averaged score for each COP-Q Module** | | | | | | | | | | |  |
| --- | --- | --- | --- | --- | --- | --- | --- | --- | --- | --- | --- |
| **Missing Days** | **Days averaged for weekly score** | **Eye Pain Frequency** | **Eye Pain Severity AM** | **Eye Pain Severity PM** | **Symptom Module 24hr recall** | **Symptom Module 24h recall (Minus Item 7)** | **Symptom Module 4hr recall; AM** | **Symptom Module 4hr recall; AM (Minus Item 7)** | **Symptom Module 4hr recall; PM** | **Symptom Module 4hr recall; PM (Minus Item 7)** |  |
|  |  |  |  |  |  |  |  |  |  |  |  |
| 0 days | 7 days | 0.814 | 0.940 | 0.940 | 0.917 | 0.910 | 0.916 | 0.915 | 0.915 | 0.917 |  |
| 1 days | 6 days | 0.790 | 0.931 | 0.931 | 0.904 | 0.897 | 0.903 | 0.902 | 0.902 | 0.904 |  |
| 2 days | 5 days | 0.758 | 0.918 | 0.918 | 0.888 | 0.878 | 0.886 | 0.885 | 0.885 | 0.888 |  |
| 3 days | 4 days | 0.714 | 0.900 | 0.900 | 0.863 | 0.852 | 0.862 | 0.860 | 0.860 | 0.863 |  |
| 4 days | 3 days | 0.652 | 0.870 | 0.870 | 0.826 | 0.813 | 0.824 | 0.822 | 0.822 | 0.826 |  |
| 5 days | 2 days | 0.556 | 0.817 | 0.817 | 0.759 | 0.743 | 0.757 | 0.755 | 0.755 | 0.759 |  |
| 6 days | 1 day | 0.385 | 0.691 | 0.691 | 0.612 | 0.591 | 0.609 | 0.606 | 0.606 | 0.612 |  |
|  | | | | | | | | | | |  |
