# Supplementary material for: Psychometric validation of the Chronic Ocular Pain Questionnaire (COP-Q)
Source: J Patient Rep Outcomes. 2025 Mar 12;9:32. doi: 10.1186/s41687-025-00862-9 (PMC11903982; doi:10.1186/s41687-025-00862-9)
Supplement: Supplementary file 16 — Supplementary Material 16 [file 41687_2025_862_MOESM16_ESM.docx]

## Supplementary 16. Convergent validity results

| Table 1. Correlations for the Eye Pain Severity (AM and PM) Module | | | | | | | | | | |
| --- | --- | --- | --- | --- | --- | --- | --- | --- | --- | --- |
|  |  | **Psychometric Analysis Population (N=124)** | | | | | | | | |
| **Convergent Measure** | **Domain** | | **Daily Score** | | | | **7-day Average Score** | | | |
|  |  |  | **Eye Pain Severity AM** | | **Eye Pain Severity PM** | | **Eye Pain Severity AM** | | **Eye Pain Severity PM** | |
|  |  |  | **n** | **Correlation Coefficient** | **n** | **Correlation Coefficient** | **n** | **Correlation Coefficient** | **n** | **Correlation Coefficient** |
| VFQ-25 | Ocular Pain | | 115 | -0.331 | 118 | -0.312 | 123 | -0.332 | 123 | -0.333 |
|  | Near Activities | | 115 | -0.237 | 118 | -0.300 | 123 | -0.205 | 123 | -0.163 |
| OPAS | Eye Pain Intensity 24 Hours | | 114 | 0.690 | 118 | 0.680 | 123 | 0.801 | 123 | 0.847 |
|  | Eye Pain Intensity 2 Weeks | | 114 | 0.682 | 118 | 0.676 | 123 | 0.831 | 123 | 0.840 |
|  | Non-Eye Pain | | 114 | 0.404 | 118 | 0.303 | 123 | 0.360 | 123 | 0.346 |
|  | Quality of Life | | 114 | 0.452 | 118 | 0.421 | 123 | 0.569 | 123 | 0.548 |
| WPAI + CIQ | Workplace Presenteeism | | 53 | 0.452 | 55 | 0.526 | 58 | 0.528 | 58 | 0.582 |
|  | Workplace Productivity Loss | | 52 | 0.472 | 54 | 0.506 | 57 | 0.534 | 57 | 0.580 |
|  | Classroom Absenteeism | | 4 | 0.174 | 6 | -0.457 | 6 | -0.218 | 6 | -0.371 |
|  | Classroom Presenteeism | | 4 | 0.905 | 6 | 0.843 | 6 | 0.891 | 6 | 0.649 |
|  | Classroom Productivity Loss | | 4 | 0.836 | 6 | 0.478 | 6 | 0.646 | 6 | 0.353 |
| The COP-Q psychometric analysis population includes participants enrolled into the study with at least one item completed on the COP-Q at any time point.  A higher score on the Eye Pain Severity Modules reflects greater impairment and a higher score on the VFQ-25 indicates better functioning.  Pearson’s correlation coefficient used for all associations.  Sample size for WPAI+CIQ; Classroom domains are very small <10 and results are not critically appraised. | | | | | | | | | | |

|  |  |  | | **Psychometric Analysis Population (N=124)** | | | |
| --- | --- | --- | --- | --- | --- | --- | --- |
|  |  | | **Daily Score** **†** | | | **7-day Average Score††** | |
| **Convergent Measure** | **Domain** | | **n** | | **Eye Pain Frequency** | **n** | **Eye Pain Frequency** |
| VFQ-25 | Ocular Pain | | 115 | | -0.280 | 123 | -0.293 |
| OPAS | Eye Pain Intensity 24 Hours | | 115 | | 0.588 | 123 | 0.737 |
|  | Eye Pain Intensity 2 Weeks | | 115 | | 0.605 | 123 | 0.752 |
|  | Quality of Life | | 115 | | 0.429 | 123 | 0.544 |
| WPAI + CIQ | Workplace Presenteeism | | 53 | | 0.427 | 58 | 0.564 |
|  | Work Productivity Loss | | 52 | | 0.392 | 57 | 0.554 |
|  | Classroom Absenteeism | | 6 | | -0.348 | 6 | -0.373 |
|  | Classroom Presenteeism | | 6 | | 0.664 | 6 | 0.802 |
|  | Classroom Productivity Loss | | 6 | | 0.494 | 6 | 0.486 |
| The COP-Q psychometric analysis population includes participants enrolled into the study with at least one item completed on the COP-Q at any time point.  A higher score on the Eye Pain Frequency Module reflects greater impairment and a higher score on the VFQ-25 indicates better functioning.  †Polyserial correlation coefficient.  †† Pearson’s correlation coefficient. | | | | | | | |

| **Table 2. Correlations for all Symptom Module recall period versions** | | | | | | | | | | | | | | | | | | | | | |
| --- | --- | --- | --- | --- | --- | --- | --- | --- | --- | --- | --- | --- | --- | --- | --- | --- | --- | --- | --- | --- | --- |
|  |  |  |  | |  | |  | |  | | **Psychometric Analysis Population (N=124)** | | | | | | | | |  |  |
|  | | **Daily Score*** | | | | | | | | | | | **7-day Average Score*** | | | | | | | | |
| **Convergent measure** | **Domain** | **n** | **Symptom Module 4-hour AM** | **n** | | **Symptom Module 4-hour PM** | | **n** | | **Symptom Module 24-hour** | | | **n** | | **Symptom Module 4-hour AM** | | **n** | **Symptom Module 4-hour PM** | | **n** | **Symptom Module 24-hour** |
| VFQ-25 | Ocular Pain | 115 | -0.348 (-0.354) | 118 | | -0.350 (-0.364) | | 114 | | -0.278 (-0.283) | | 123 | | -0.351 (-0.364) | | 123 | | -0.357 (-0.368) | 123 | | -0.388 (-0.402) |
| OPAS | Eye Pain Intensity 24 Hours | 114 | 0.662 (0.666) | 118 | | 0.682 (0.677) | | 114 | | 0.667 (0.676) | | 123 | | 0.753 (0.767) | | 123 | | 0.787 (0.799) | 123 | | 0.813 (0.824) |
|  | Eye Pain Intensity 2 Weeks | 114 | 0.661 (0.671) | 118 | | 0.691 (0.685) | | 114 | | 0.705 (0.717) | | 123 | | 0.776 (0.791) | | 123 | | 0.782 (0.797) | 123 | | 0.827 (0.839) |
|  | Non-Eye Pain | 114 | 0.390 (0.416) | 118 | | 0.325 (0.344) | | 114 | | 0.322 (0.348) | | 123 | | 0.340 (0.361) | | 123 | | 0.320 (0.343) | 123 | | 0.360 (0.383) |
|  | Quality of Life | 114 | 0.483 (0.494) | 118 | | 0.473 (0.482) | | 114 | | 0.466 (0.484) | | 123 | | 0.562 (0.583) | | 123 | | 0.541 (0.563) | 123 | | 0.574 (0.594) |
| WPAI + CIQ | Workplace Absenteeism | 52 | 0.310 (0.302) | 54 | | 0.214 (0.217) | | 53 | | 0.192 (0.187) | | 57 | | 0.297 (0.295) | | 57 | | 0.268 (0.267) | 57 | | 0.265 (0.261) |
|  | Workplace Presenteeism | 53 | 0.498 (0.478) | 55 | | 0.531 (0.527) | | 54 | | 0.550 (0.539) | | 58 | | 0.573 (0.567) | | 58 | | 0.596 (0.595) | 58 | | 0.575 (0.569) |
|  | Work Productivity Loss | 52 | 0.519 (0.501) | 54 | | 0.525 (0.525) | | 53 | | 0.537 (0.528) | | 57 | | 0.579 (0.574) | | 57 | | 0.595 (0.596) | 57 | | 0.570 (0.565) |
|  | Classroom Absenteeism | 4 | 0.422 (0.375) | 6 | | -0.433 (-0.407) | | 6 | | -0.532 (-0.520) | | 6 | | -0.373 (-0.392) | | 6 | | -0.481 (-0.436) | 6 | | -0.432 (-0.436) |
|  | Classroom Presenteeism | 4 | 0.964 (0.960) | 6 | | 0.912 (0.921) | | 6 | | 0.807 (0.760) | | 6 | | 0.802 (0.826) | | 6 | | 0.728 (0.726) | 6 | | 0.708 (0.726) |
|  | Classroom Productivity Loss | 4 | 0.932 (0.920) | 6 | | 0.554 (0.575) | | 6 | | 0.416 (0.382) | | 6 | | 0.486 (0.497) | | 6 | | 0.361 (0.364) | 6 | | 0.373 (0.387) |
| The COP-Q psychometric analysis population includes participants enrolled into the study with at least one item completed on the COP-Q at any time point.  A higher score on the Symptom Modules reflects greater impairment and a higher score on the VFQ-25 indicates better functioning.  Scores in parentheses indicate correlation results without item 7 (Eye itch).  *Pearson’s correlation coefficient | | | | | | | | | | | | | | | | | | | | | |

| Table 3. Correlations for the VTM, HRQoL and Sleep Modules | | | | | |
| --- | --- | --- | --- | --- | --- |
|  |  | **Psychometric Analysis Population (N=124)** | | | |
|  |  | **7-day Recall Scores** | | | |
| **Convergent Measure** | **Domain** | **n** | **VTM** | **HRQoL Module** | **Sleep Module** |
| VFQ-25 | Ocular Pain | 117 | -0.477 | -0.330 | -0.439 |
|  | Near Activities | 117 | -0.362 | -0.196 | - |
|  | Distance Activities | 117 | -0.378 | -0.247 | - |
| VFQ-25 (Vision Specific) | Mental Health | 117 | - | -0.322 | -0.356 |
|  | Role Difficulties | 117 | -0.438 | -0.202 | -0.232 |
| EQ-5D-5L | Usual activities | 108 | 0.405 | 0.356 | - |
|  | Anxiety/Depression | 108 | - | 0.604 | - |
|  | Vision bolt-on | 108 | 0.318 | 0.302 | 0.203 |
| OPAS | Eye Pain Intensity 24 Hours | 118 | 0.425 | 0.134 | 0.388 |
|  | Eye Pain Intensity 2 Weeks | 118 | 0.439 | 0.122 | 0.384 |
|  | Non-Eye Pain | 118 | 0.213 | 0.275 | 0.352 |
|  | Quality of Life | 118 | 0.405 | 0.332 | 0.548 |
| WPAI + CIQ | Work Productivity Loss | 53 | 0.657 | 0.490 | 0.459 |
|  | Activity Impairment | 106 | 0.490 | 0.417 | 0.555 |
|  | Classroom Productivity Loss | 5 | -0.183 | -0.601 | 0.081 |
| The COP-Q psychometric analysis population includes participants enrolled into the study with at least one item completed on the COP-Q at any time point.  A higher score on the Symptom Modules reflects greater impairment and a higher score on the VFQ-25 indicates better functioning.  * Pearson correlation coefficient is used for the VTM and HRQoL Modules and Spearman’s correlation coefficient for the Sleep Module. | | | | | |
