# Supplementary material for: Psychometric validation of the Chronic Ocular Pain Questionnaire (COP-Q)
Source: J Patient Rep Outcomes. 2025 Mar 12;9:32. doi: 10.1186/s41687-025-00862-9 (PMC11903982; doi:10.1186/s41687-025-00862-9)
Supplement: Supplementary file 17 — Supplementary Material 17 [file 41687_2025_862_MOESM17_ESM.docx]

## Supplementary 17. Known-groups validity

| Table 1. Known-groups validity comparisons for the Eye Pain Severity Module | | | | | | | | | | |
| --- | --- | --- | --- | --- | --- | --- | --- | --- | --- | --- |
|  | **AM** | | | | | **PM** | | | | |
| **Item/Score Anchor** | **n** | **Median Score** | **Mean (SD)** | **Between groups effect size** | **Pairwise p-value** | **n** | **Median Score** | **Mean (SD)** | **Between groups effect size** | **Pairwise p-value** |
| COSP Severity (clinician-rated)* | | | | | | | | | | |
| Mild (reference) | 16 | 3.1 | 3.9 (3.37) | - | - | 16 | 2.9 | 3.7 (2.89) | - | - |
| Moderate | 75 | 3.0 | 3.5 (2.33) | -0.18 | 1 | 75 | 4.3 | 4.0 (2.35) | 0.12 | 1 |
| Severe/Very Severe | 33 | 6.1 | 6.3 (2.06) | 0.94 | <0.001 | 33 | 6.9 | 6.6 (1.77) | 1.32 | <0.001 |
| COSP Severity (patient-reported)* | | | | | | | | | | |
| Mild (pain score of 1-3 on a 0-10 scale) (reference) | 8 | 2.5 | 3.0 (2.62) | - | - | 8 | 2.4 | 2.5 (1.46) | - | - |
| Moderate (pain score of 4-6 on a 0-10 scale) | 55 | 3.0 | 3.1 (2.21) | 0.03 | 1 | 55 | 3.6 | 3.6 (2.23) | 0.55 | 0.500 |
| Severe (pain score of 7-10 on a 0-10 scale) | 61 | 6.0 | 5.6 (2.55) | 1.00 | 0.017 | 61 | 6.4 | 5.9 (2.34) | 1.51 | <0.001 |
| PGI-S Eye Pain* | | | | | | | | | | |
| Response of 1 – Mild (Reference) | 48 | 2.7 | 2.9 (1.87) | - | - | 48 | 3.3 | 3.4 (1.85) | - | - |
| Response of 2 – Moderate | 45 | 5.8 | 5.6 (2.23) | 1.29 | <0.001 | 45 | 5.9 | 5.9 (1.77) | 1.42 | <0.001 |
| Response of 3 – Severe | 9 | 8.0 | 7.6 (1.18) | 2.62 | <0.001 | 9 | 8.0 | 7.8 (1.08) | 2.45 | <0.001 |
| PGI-S Eye Pain and Related Problems* | | | | | | | | | | |
| Response of 1 - Mild (Reference) | 49 | 2.7 | 2.9 (2.09) | - | - | 49 | 3.0 | 3.2 (1.90) | - | - |
| Response of 2 - Moderate | 46 | 5.3 | 5.1 (2.15) | 1.03 | <0.001 | 46 | 5.6 | 5.7 (1.78) | 1.36 | <0.001 |
| Response of 3 - Severe | 11 | 8.2 | 7.9 (1.08) | 2.56 | <0.001 | 11 | 8.1 | 7.9 (1.10) | 2.66 | <0.001 |
| PGI-S Limitations in Visual Activities* | | | | | | | | | | |
| Response of 0 - None | 25 | 2.0 | 2.8 (2.86) | -0.39 | 0.540 | 25 | 1.9 | 2.8 (2.61) | -0.60 | 0.047 |
| Response of 1 - Mild (Reference) | 51 | 3.3 | 3.7 (2.18) | - | - | 51 | 4.0 | 4.1 (2.04) | - | - |
| Response of 2 - Moderate | 28 | 5.4 | 5.1 (2.13) | 0.65 | 0.062 | 28 | 5.8 | 5.9 (1.65) | 0.91 | 0.002 |
| Response of 3 - Severe | 8 | 8.6 | 8.2 (1.11) | 2.16 | <0.001 | 8 | 8.2 | 8.3 (0.63) | 2.20 | <0.001 |
| Population includes all patients in the psychometric analysis population with at least one completed COP-Q item at any timepoint.  The between-groups effect size is using Hedge's g compared to the reference group (ref). Hedge's g is calculated as the difference in means ((comparison group) - (reference group)) divided by the pooled standard deviation.  Pair-wise p-values are from two-sample t-tests testing mean score differences between corresponding group and reference group. P-values are adjusted for multiple comparisons using Bonferroni correction.  *Indicates a significant F-test value (p<0.05).  SD: Standard Deviation; PGI-S: Patient global impression of severity; COSP: Chronic Ocular Surface Pain. | | | | | | | | | | |

| Table 2. Known-groups validity comparisons for the Eye Pain Frequency Module | | | | | |
| --- | --- | --- | --- | --- | --- |
| **Item/Score Anchor** | **n** | **Median Score** | **Mean (SD)** | **Between groups effect size** | **Pairwise p-value** |
| COSP Severity (clinician-rated)* | | | | | |
| Mild (reference) | 16 | 1.8 | 1.7 (0.95) | - | - |
| Moderate | 75 | 1.7 | 1.8 (0.78) | 0.09 | 1 |
| Severe | 33 | 2.6 | 2.6 (0.57) | 1.27 | <0.001 |
| COSP Severity (patient-reported)* | | | | | |
| Mild (pain score of 1-3 on a 0-10 scale) (reference) | 8 | 1.5 | 1.4 (0.61) | - | - |
| Moderate (pain score of 4-6 on a 0-10 scale) | 55 | 1.7 | 1.7 (0.76) | 0.46 | 0.739 |
| Severe (pain score of 7-10 on a 0-10 scale) | 61 | 2.1 | 2.3 (0.79) | 1.24 | 0.004 |
| PGI-S Eye Pain* | | | | | |
| Response of 1 - Mild (Reference) | 42 | 1.6 | 1.5 (0.64) | - | - |
| Response of 2 - Moderate | 61 | 2.2 | 2.3 (0.65) | 1.21 | <0.001 |
| Response of 3 - Severe | 10 | 3.1 | 3.0 (0.42) | 2.43 | <0.001 |
| PGI-S Eye Pain and Related Problems* | | | | | |
| Response of 1 - Mild (Reference) | 38 | 1.3 | 1.4 (0.57) | - | - |
| Response of 2 - Moderate | 58 | 2.2 | 2.4 (0.64) | 1.56 | <0.001 |
| Response of 3 - Severe | 14 | 3.0 | 2.9 (0.48) | 2.70 | <0.001 |
| PGI-S Limitations in Visual Activities* | | | | | |
| Response of 1 - Mild (Reference) | 65 | 2.0 | 1.8 (0.73) | - | - |
| Response of 2 - Moderate | 35 | 2.3 | 2.5 (0.68) | 0.92 | <0.001 |
| Response of 3 - Severe | 5 | 3.0 | 3.0 (0.13) | 1.68 | 0.003 |
| Population includes all patients in the psychometric analysis population with at least one completed COP-Q item at any timepoint.  The between-groups effect size is using Hedge's g compared to the reference group (ref). Hedge's g is calculated as the difference in means ((comparison group) - (reference group)) divided by the pooled standard deviation. Pair-wise p-values are from two-sample t-tests testing mean score differences between corresponding group and reference group. P-values are adjusted for multiple comparisons using Bonferroni correction.  *Indicates a significant F-test value (p<0.05).  SD: Standard Deviation; PGI-S: Patient global impression of severity; COSP: Chronic Ocular Surface Pain. | | | | | |

| Table 3. Known-groups validity comparisons for the Symptom Module (4-hour AM) 7-day average scores | | | | | |
| --- | --- | --- | --- | --- | --- |
| **Item/Score Anchor** | **n** | **Median Score** | **Mean (SD)** | **Between groups effect size** | **Pairwise p-value** |
| COSP Severity (clinician-rated)* | | | | | |
| Mild (reference) | 16 | 23.7 | 25.7 (21.55) | - | - |
| Moderate | 75 | 21.7 | 24.4 (14.49) | -0.08 | 1 |
| Severe | 33 | 42.5 | 42.4 (13.89) | 1.00 | 0.002 |
| COSP Severity (patient-reported)* | | | | | |
| Mild (pain score of 1-3 on a 0-10 scale) (reference) | 8 | 17.1 | 18.8 (12.21) | - | - |
| Moderate (pain score of 4-6 on a 0-10 scale) | 55 | 18.6 | 21.0 (14.09) | 0.16 | 1 |
| Severe (pain score of 7-10 on a 0-10 scale) | 61 | 38.2 | 38.3 (15.68) | 1.27 | 0.002 |
| PGI-S Eye Pain* | | | | | |
| Response of 1 - Mild (Reference) | 48 | 18.7 | 21.9 (12.81) | - | - |
| Response of 2 - Moderate | 45 | 34.4 | 35.5 (14.63) | 0.99 | <0.001 |
| Response of 3 - Severe | 9 | 52.4 | 48.4 (11.31) | 2.10 | <0.001 |
| PGI-S Eye Pain and Related Problems* | | | | | |
| Response of 1 - Mild (Reference) | 49 | 18.3 | 19.7 (11.66) | - | - |
| Response of 2 – Moderate | 46 | 33.9 | 34.5 (13.05) | 1.20 | <0.001 |
| Response of 3 – Severe | 11 | 57.3 | 53.5 (10.04) | 2.97 | <0.001 |
| PGI-S Limitations in Visual Activities* | | | | | |
| Response of 0 - None | 25 | 17.1 | 17.4 (14.39) | -0.60 | 0.092 |
| Response of 1 - Mild (Reference) | 51 | 23.4 | 25.6 (13.33) | - | - |
| Response of 2 - Moderate | 28 | 35.1 | 35.1 (14.52) | 0.69 | 0.024 |
| Response of 3 - Severe | 8 | 58.9 | 55.8 (9.77) | 2.33 | <0.001 |
| Population includes all patients in the psychometric analysis population with at least one completed COP-Q item at any timepoint.  The between-groups effect size is using Hedge's g compared to the reference group (ref). Hedge's g is calculated as the difference in means ((comparison group) - (reference group)) divided by the pooled standard deviation. Pair-wise p-values are from two-sample t-tests testing mean score differences between corresponding group and reference group. P-values are adjusted for multiple comparisons using Bonferroni correction.  *Indicates a significant F-test value (p<0.05).  SD: Standard Deviation; PGI-S: Patient global impression of severity; COSP: Chronic Ocular Surface Pain. | | | | | |

| Table 4. Known-groups validity comparisons for the Symptom Module (4-hour PM) 7-day average scores | | | | | | | | | | | | | | |
| --- | --- | --- | --- | --- | --- | --- | --- | --- | --- | --- | --- | --- | --- | --- |
| **Item/Score Anchor** | | | | **n** | | | **Median Score** | | | **Mean (SD)** | | | **Between groups effect size** | **Pairwise p-value** |
| COSP Severity (clinician-rated)* | | | | | | | | | | | | | | |
| Mild (reference) | | | | 16 | | | 16.3 | | | 24.5 (19.86) | | | - | - |
| Moderate | | | | 75 | | | 23.8 | | | 27.5 (14.31) | | | 0.19 | 1.00 |
| Severe | | | | 33 | | | 43.6 | | | 44.2 (13.24) | | | 1.25 | <0.001 |
| COSP Severity (patient-reported)* | | | | | | | | | | | | | | |
| Mild (pain score of 1-3 on a 0-10 scale) (reference) | | | | 8 | | | 15.6 | | | 18.1 (9.86) | | | - | - |
| Moderate (pain score of 4-6 on a 0-10 scale) | | | | 55 | | | 21.0 | | | 23.4 (13.96) | | | 0.40 | 0.934 |
| Severe (pain score of 7-10 on a 0-10 scale) | | | | 61 | | | 42.0 | | | 40.7 (14.40) | | | 1.61 | <0.001 |
| PGI-S Eye Pain* | | | | | | | | | | | | | | |
| Response of 1 - Mild (Reference) | | | | 48 | | | 20.0 | | | 23.9 (12.44) | | | - | - |
| Response of 2 - Moderate | | | | 45 | | | 38.7 | | | 38.1 (13.55) | | | 1.09 | <0.001 |
| Response of 3 - Severe | | | | 9 | | | 52.4 | | | 49.9 (11.05) | | | 2.13 | <0.001 |
| PGI-S Eye Pain and Related Problems* | | | | | | | | | | | | | | |
| Response of 1 - Mild (Reference) | | | | 49 | | | 19.4 | | | 21.2 (11.05) | | | - | - |
| Response of 2 – Moderate | | | | 46 | | | 39.1 | | | 38.1 (11.44) | | | 1.50 | <0.001 |
| Response of 3 – Severe | | | | 11 | | | 58.8 | | | 53.6 (11.12) | | | 2.93 | <0.001 |
| PGI-S Limitations in Visual Activities* | | | | | | | | | | | | | | |
| Response of 0 – None | | | | 25 | | | 15.0 | | | 17.9 (13.92) | | | -0.74 | 0.012 |
| Response of 1 - Mild (Reference) | | | | 51 | | | 24.6 | | | 27.7 (12.81) | | | - | - |
| Response of 2 - Moderate | | | | 28 | | | 40.4 | | | 39.3 (12.39) | | | 0.92 | <0.001 |
| Response of 3 - Severe | | | | 8 | | | 59.2 | | | 57.4 (6.63) | | | 2.44 | <0.001 |
| Population includes all patients in the psychometric analysis population with at least one completed COP-Q item at any timepoint.  The between-groups effect size is using Hedge's g compared to the reference group (ref). Hedge's g is calculated as the difference in means ((comparison group) - (reference group)) divided by the pooled standard deviation. Pair-wise p-values are from two-sample t-tests testing mean score differences between corresponding group and reference group. P-values are adjusted for multiple comparisons using Bonferroni correction.  SD: Standard Deviation; PGI-S: Patient global impression of severity; COSP: Chronic Ocular Surface Pain. | | | | | | | | | | | | | | |
| **Table 5. Known-groups validity comparisons for the Symptom Module (24-hour)** | | | | | | | | | | | | | | |
| **Item/Score Anchor** | | **n** | **Median Score** | | | | | **Mean (SD)** | | | | **Between groups effect size** | | **Pairwise p-value** |
|  | COSP Severity (clinician-rated)* | | | | | | | | | | | | | |
| Mild (reference) | | 16 | 25.0 | | | | | 26.8 (19.82) | | | | - | | - |
| Moderate | | 75 | 24.9 | | | | | 28.0 (13.79) | | | | 0.08 | | 1.00 |
| Severe | | 32 | 48.0 | | | | | 45.7 (12.13) | | | | 1.25 | | <0.001 |
|  | COSP Severity (patient-reported)* | | | | | | | | | | | | | |
| Mild (pain score of 1-3 on a 0-10 scale) (reference) | | 8 | 18.0 | | | | | 19.2 (11.24) | | | | - | | - |
| Moderate (pain score of 4-6 on a 0-10 scale) | | 55 | 20.7 | | | | | 23.6 (13.07) | | | | 0.34 | | 1.00 |
| Severe (pain score of 7-10 on a 0-10 scale) | | 61 | 44.1 | | | | | 42.4 (13.20) | | | | 1.78 | | <0.001 |
|  | PGI-S Eye Pain* | | | | | | | | | | | | | |
| Response of 1 – Mild (Reference) | | 43 | 22.3 | | | | | 24.5 (12.47) | | | | - | | - |
| Response of 2 – Moderate | | 54 | 37.4 | | | | | 37.2 (13.53) | | | | 0.97 | | <0.001 |
| Response of 3 – Severe | | 13 | 48.9 | | | | | 49.1 (10.58) | | | | 2.04 | | <0.001 |
|  | PGI-S Eye Pain and Related Problems* | | | | | | | | | | | | | |
| Response of 1 – Mild (Reference) | | 39 | 21.4 | | | | | 23.1 (11.06) | | | | - | | - |
| Response of 2 – Moderate | | 54 | 37.9 | | | | | 37.9 (13.58) | | | | 1.17 | | <0.001 |
| Response of 3 – Severe | | 11 | 49.0 | | | | | 51.4 (7.75) | | | | 2.70 | | <0.001 |
|  | PGI-S Limitations in Visual Activities* | | | | | | | | | | | | | |
| Response of 0 – None | | 23 | 22.3 | | | | | 26.6 (16.24) | | | | -0.15 | | 1.00 |
| Response of 1 – Mild (Reference) | | 55 | 29.1 | | | | | 28.9 (13.89) | | | | - | | - |
| Response of 2 – Moderate | | 33 | 43.1 | | | | | 40.8 (13.16) | | | | 0.88 | | 0.001 |
| Response of 3 – Severe | | 3 | 59.2 | | | | | 59.9 (1.31) | | | | 2.27 | | 0.002 |
|  | Population includes all patients in the psychometric analysis population with at least one completed COP-Q item at any timepoint.  The between groups effect size is using Hedge's g compared to the reference group (ref). Hedge's g is calculated as the difference in means ((comparison group) - (reference group)) divided by the pooled standard deviation. Pair-wise p-values are from two-sample t-tests testing mean score differences between corresponding group and reference group. P-values are adjusted for multiple comparisons using Bonferroni correction.  SD: Standard Deviation; PGI-S: Patient global impression of severity; COSP: Chronic Ocular Surface Pain. | | | | | | | | | | | | | |
| **Table 6. Known-groups validity comparisons for the VTM** | | | | | | | | | | | | | | |
| **Item/Score Anchor** | | | | | **n** | **Median Score** | | | **Mean (SD)** | | **Between groups effect size** | | | **Pairwise p-value** |
| COSP Severity (clinician-rated)* | | | | | | | | | | | | | | |
| Mild (reference) | | | | | 15 | 3.0 | | | 5.0 (5.07) | | - | | | - |
| Moderate | | | | | 71 | 7.0 | | | 7.7 (4.33) | | 0.61 | | | 0.135 |
| Severe | | | | | 32 | 9.0 | | | 10.4 (5.40) | | 1.01 | | | <0.001 |
| COSP Severity (patient-reported)* | | | | | | | | | | | | | | |
| Mild (pain score of 1-3 on a 0-10 scale) (reference) | | | | | 8 | 6.5 | | | 7.6 (5.50) | | - | | | - |
| Moderate (pain score of 4-6 on a 0-10 scale) | | | | | 52 | 6.0 | | | 6.6 (4.57) | | -0.22 | | | 1.00 |
| Severe (pain score of 7-10 on a 0-10 scale) | | | | | 58 | 9.0 | | | 9.6 (4.97) | | 0.40 | | | 0.828 |
| PGI-S Eye Pain* | | | | | | | | | | | | | | |
| Response of 1 - Mild (Reference) | | | | | 47 | 5.0 | | | 5.6 (4.24) | | - | | | - |
| Response of 2 - Moderate | | | | | 61 | 9.0 | | | 9.3 (4.30) | | 0.86 | | | 0.002 |
| Response of 3 - Severe | | | | | 10 | 12.5 | | | 13.5 (6.06) | | 1.73 | | | <0.001 |
| PGI-S Eye Pain and Related Problems* | | | | | | | | | | | | | | |
| Response of 1 - Mild (Reference) | | | | | 46 | 5.0 | | | 5.1 (3.67) | | - | | | - |
| Response of 2 - Moderate | | | | | 58 | 9.5 | | | 9.5 (4.24) | | 1.11 | | | <0.001 |
| Response of 3 - Severe | | | | | 14 | 12.0 | | | 12.7 (6.02) | | 1.78 | | | <0.001 |
| PGI-S Limitations in Visual Activities* | | | | | | | | | | | | | | |
| Response of 1 - Mild (Reference) | | | | | 78 | 6.0 | | | 6.1 (4.05) | | - | | | - |
| Response of 2 - Moderate | | | | | 35 | 12.0 | | | 11.8 (4.24) | | 1.39 | | | <0.001 |
| Response of 3 - Severe | | | | | 5 | 16.0 | | | 14.2 (4.71) | | 1.98 | | | <0.001 |
| Population includes all patients in the psychometric analysis population with at least one completed COP-Q item at any timepoint.  The between-groups effect size is using Hedge's g compared to the reference group (ref). Hedge's g is calculated as the difference in means ((comparison group) - (reference group)) divided by the pooled standard deviation. Pair-wise p-values are from two-sample t-tests testing mean score differences between corresponding group and reference group. P-values are adjusted for multiple comparisons using Bonferroni correction.  *Indicates a significant F-test value (p<0.05).  SD: Standard Deviation; PGI-S: Patient global impression of severity; COSP: Chronic Ocular Surface Pain. | | | | | | | | | | | | | | |

| **Table 7. Known-groups validity comparisons for the HRQoL Module** | | | | | |
| --- | --- | --- | --- | --- | --- |
| **Item/Score Anchor** | **n** | **Median Score** | **Mean (SD)** | **Between groups effect size** | **Pairwise p-value** |
| COSP Severity (clinician-rated)* | | | | | |
| Mild (reference) | 15 | 1.0 | 2.3 (2.97) | - | - |
| Moderate | 71 | 4.0 | 4.2 (3.52) | 0.55 | 0.254 |
| Severe | 31 | 5.0 | 6.0 (4.81) | 0.85 | 0.008 |
| COSP Severity (patient-reported) | | | | | |
| Mild (pain score of 1-3 on a 0-10 scale) (reference) | 8 | 3.5 | 3.8 (2.38) | - | - |
| Moderate (pain score of 4-6 on a 0-10 scale) | 52 | 4.0 | 4.2 (3.69) | 0.11 | 1.00 |
| Severe (pain score of 7-10 on a 0-10 scale) | 58 | 4.0 | 4.9 (4.36) | 0.27 | 1.00 |
| PGI-S Eye Pain* | | | | | |
| Response of 1 - Mild (Reference) | 47 | 2.0 | 3.0 (3.04) | - | - |
| Response of 2 - Moderate | 61 | 5.0 | 4.7 (3.65) | 0.52 | 0.093 |
| Response of 3 - Severe | 10 | 9.5 | 10.0 (4.64) | 2.10 | <0.001 |
| PGI-S Eye Pain and Related Problems* | | | | | |
| Response of 1 - Mild (Reference) | 46 | 2.0 | 2.7 (2.79) | - | - |
| Response of 2 - Moderate | 58 | 5.0 | 4.9 (3.85) | 0.64 | 0.023 |
| Response of 3 - Severe | 14 | 8.0 | 8.3 (4.70) | 1.67 | <0.001 |
| PGI-S Limitations in Visual Activities* | | | | | |
| Response of 1 - Mild (Reference) | 78 | 2.0 | 3.1 (2.82) | - | - |
| Response of 2 - Moderate | 35 | 8.0 | 6.8 (4.42) | 1.10 | <0.001 |
| Response of 3 - Severe | 5 | 8.0 | 10.0 (4.00) | 2.40 | <0.001 |
| Population includes all patients in the psychometric analysis population with at least one completed COP-Q item at any timepoint.  The between groups effect size is using Hedge's g compared to the reference group (ref). Hedge's g is calculated as the difference in means ((comparison group) - (reference group)) divided by the pooled standard deviation. Pair-wise p-values are from two-sample t-tests testing mean score differences between corresponding group and reference group. P-values are adjusted for multiple comparisons using Bonferroni correction.  *Indicates a significant F-test value (p<0.05).  SD: Standard Deviation; PGI-S: Patient global impression of severity; COSP: Chronic Ocular Surface Pain; HRQoL: Health-related Quality of Life. | | | | | |

| **Table 8. Known-groups validity comparisons for the Sleep Module** | | | | | |
| --- | --- | --- | --- | --- | --- |
| **Item/Score Anchor** | **n** | **Median Score** | **Mean (SD)** | **Between groups effect size** | **Pairwise p-value** |
| COSP Severity (clinician-rated)* | | | | | |
| Mild (reference) | 15 | 0.0 | 0.7 (1.44) | - | - |
| Moderate | 71 | 1.0 | 0.9 (0.96) | 0.15 | 1.00 |
| Severe | 32 | 2.0 | 1.6 (1.12) | 0.69 | 0.034 |
| COSP Severity (patient-reported)* | | | | | |
| Mild (pain score of 1-3 on a 0-10 scale) (reference) | 8 | 0.0 | 0.5 (0.76) | - | - |
| Moderate (pain score of 4-6 on a 0-10 scale) | 52 | 1.0 | 0.8 (0.87) | 0.34 | 1.00 |
| Severe (pain score of 7-10 on a 0-10 scale) | 58 | 1.0 | 1.4 (1.25) | 0.73 | 0.095 |
| PGI-S Eye Pain* | | | | | |
| Response of 1 - Mild (Reference) | 47 | 0.0 | 0.6 (0.71) | - | - |
| Response of 2 - Moderate | 61 | 1.0 | 1.2 (1.16) | 0.66 | 0.013 |
| Response of 3 - Severe | 10 | 2.5 | 2.3 (1.16) | 2.15 | <0.001 |
| PGI-S Eye Pain and Related Problems* | | | | | |
| Response of 1 - Mild (Reference) | 46 | 0.0 | 0.5 (0.62) | - | - |
| Response of 2 - Moderate | 58 | 1.0 | 1.3 (1.15) | 0.85 | <0.001 |
| Response of 3 - Severe | 14 | 2.0 | 2.0 (1.24) | 1.89 | <0.001 |
| PGI-S Limitations in Visual Activities* | | | | | |
| Response of 1 - Mild (Reference) | 78 | 1.0 | 0.6 (0.68) | - | - |
| Response of 2 - Moderate | 35 | 2.0 | 1.8 (1.37) | 1.22 | <0.001 |
| Response of 3 - Severe | 5 | 3.0 | 2.4 (0.89) | 2.53 | 0.001 |
| Population includes all patients in the psychometric analysis population with at least one completed COP-Q item at any timepoint.  The between-groups effect size is using Hedge's g compared to the reference group (ref). Hedge's g is calculated as the difference in means ((comparison group) - (reference group)) divided by the pooled standard deviation. Pair-wise p-values are from two-sample t-tests testing mean score differences between corresponding group and reference group. P-values are adjusted for multiple comparisons using Bonferroni correction.  SD: Standard Deviation; PGI-S: Patient global impression of severity; COSP: Chronic Ocular Surface Pain. | | | | | |
