# Supplementary material for: Psychometric validation of the Chronic Ocular Pain Questionnaire (COP-Q)
Source: J Patient Rep Outcomes. 2025 Mar 12;9:32. doi: 10.1186/s41687-025-00862-9 (PMC11903982; doi:10.1186/s41687-025-00862-9)
Supplement: Supplementary file 18 — Supplementary Material 18 [file 41687_2025_862_MOESM18_ESM.docx]

## Supplementary 18. R packages used

**R version used 4.1.1 (2021-08-10)**

| base | packages: |  |
| --- | --- | --- |
| stats | graphics | grDevices |
|  |  |  |
| attached | packages: |  |
| lubridate_1.9.2 | forcats_1.0.0 | stringr_1.5.0 |
| dplyr_1.1.0 | purrr_1.0.1 | readr_2.1.4 |
| tidyr_1.3.0 | tibble_3.2.0 | tidyverse_2.0.0 |
| rhdf5_2.38.1 | qgraph_1.9.3 | readxl_1.4.2 |
| kableExtra_1.3.4 | devtools_2.4.5 | usethis_2.1.6 |
| Hmisc_5.0-1 | BiocManager_1.30.20 | ggplot2_3.4.1 |
| psych_2.2.9 | semPlot_1.1.6 | pander_0.6.5 |
| icesTAF_4.1.0 | MplusAutomation_1.1.0 | pacman_0.5.1 |
| TAM_4.1-4 | CDM_8.2-6 | mvtnorm_1.1-3 |
| paran_1.5.2 | relimp_1.0-5 | RColorBrewer_1.1-3 |
| mirt_1.37.1 | EGAnet_1.2.3 | forcats_0.5.2 |
| stringr_1.5.0 | dplyr_1.0.10 | purrr_1.0.1 |
| readr_2.1.2 | tidyr_1.3.0 | tibble_3.1.8 |
| tidyverse_1.3.2 | knitr_1.40 | ShinyItemAnalysis_1.4.2 |
| ggmirt_0.1.0 | rhdf5_2.36.0 | kableExtra_1.3.4 |
| plyr_1.8.7 | haven_2.5.1 | icesTAF_4.0.0 |
| TAF_4.0.0 | semTools_0.5-6 | semPlot_1.1.6 |
| pander_0.6.5 | corrplot_0.92 | igraph_1.3.4 |
| qgraph_1.9.2 | mokken_3.0.6 | eRm_1.0-2 |
| ltm_1.2-0 | polycor_0.8-1 | msm_1.6.9 |
| poLCA_1.6.0.1 | MASS_7.3-54 | scatterplot3d_0.3-42 |
| lavaan_0.6-12 | psych_2.2.5 | Hmisc_4.7-1 |
| ggplot2_3.4.0 | Formula_1.2-4 | survival_3.2-11 |
| lattice_0.20-44 | car_3.1-0 | carData_3.0-5 |
| MplusAutomation_1.1.0 | |  |
| Formula_1.2-5 | stats4_4.1.1 | profvis_0.3.7 |
| htmlwidgets_1.6.1 | httr_1.4.5 | lavaan_0.6-15 |
| ellipsis_0.3.2 | urlchecker_1.0.1 | pkgconfig_2.0.3 |
| XML_3.99-0.13 | nnet_7.3-18 | sass_0.4.5 |
| kutils_1.70 | utf8_1.2.3 | tidyselect_1.2.0 |
| rlang_1.1.0 | reshape2_1.4.4 | later_1.3.0 |
| cellranger_1.1.0 | munsell_0.5.0 | tools_4.1.1 |
| cachem_1.0.7 | cli_3.6.0 | gsubfn_0.7 |
| generics_0.1.3 | fdrtool_1.2.17 | evaluate_0.20 |
| fastmap_1.1.1 | arm_1.13-1 | yaml_2.3.7 |
| processx_3.8.0 | knitr_1.42 | fs_1.5.2 |
| zip_2.2.2 | glasso_1.11 | pbapply_1.7-0 |
| nlme_3.1-162 | mime_0.12 | xml2_1.3.3 |
| compiler_4.1.1 | rstudioapi_0.14 | png_0.1-8 |
| TAF_4.1.0 | bslib_0.4.2 | pbivnorm_0.6.0 |
| stringi_1.7.12 | highr_0.10 | ps_1.7.2 |
| rockchalk_1.8.157 | lattice_0.20-45 | texreg_1.38.6 |
| Matrix_1.5-3 | nloptr_2.0.3 | vctrs_0.5.2 |
| rhdf5filters_1.6.0 | pillar_1.8.1 | lifecycle_1.0.3 |
| jquerylib_0.1.4 | OpenMx_2.21.1 | data.table_1.14.8 |
| corpcor_1.6.10 | httpuv_1.6.9 | R6_2.5.1 |
| promises_1.2.0.1 | gridExtra_2.3 | codetools_0.2-19 |
| sessioninfo_1.2.2 | boot_1.3-28.1 | fastDummies_1.6.3 |
| MASS_7.3-58.3 | gtools_3.9.4 | pkgload_1.3.2 |
| proto_1.0.0 | withr_2.5.0 | mnormt_2.1.1 |
| hms_1.1.2 | parallel_4.1.1 | quadprog_1.5-8 |
| grid_4.1.1 | rpart_4.1.19 | coda_0.19-4 |
| minqa_1.2.5 | rmarkdown_2.20 | carData_3.0-5 |
| shiny_1.7.3 | base64enc_0.1-3 |  |
|  |  |  |
|  |  |  |
| utf8_1.2.2 | proto_1.0.0 | tidyselect_1.2.0 |
| lme4_1.1-30 | htmlwidgets_1.5.4 | grid_4.1.1 |
| munsell_0.5.0 | codetools_0.2-18 | interp_1.1-3 |
| withr_2.5.0 | colorspace_2.1-0 | highr_0.9 |
| OpenMx_2.20.6 | rstudioapi_0.14 | labeling_0.4.2 |
| mi_1.1 | mnormt_2.1.0 | farver_2.1.1 |
| coda_0.19-4 | vctrs_0.5.2 | generics_0.1.3 |
| xfun_0.32 | R6_2.5.1 | arm_1.13-1 |
| deltaPlotR_1.6 | reshape_0.8.9 | rhdf5filters_1.4.0 |
| cachem_1.0.6 | assertthat_0.2.1 | scales_1.2.1 |
| nnet_7.3-16 | googlesheets4_1.0.1 | texreg_1.38.6 |
| gtable_0.3.1 | rlang_1.0.6 | systemfonts_1.0.4 |
| splines_4.1.1 | gargle_1.2.1 | broom_1.0.1 |
| checkmate_2.1.0 | yaml_2.3.5 | reshape2_1.4.4 |
| abind_1.4-5 | modelr_0.1.9 | backports_1.4.1 |
| tools_4.1.1 | statnet.common_4.6.0 | ellipsis_0.3.2 |
| jquerylib_0.1.4 | gsubfn_0.7 | Rcpp_1.0.10 |
| base64enc_0.1-3 | rockchalk_1.8.157 | rpart_4.1-15 |
| deldir_1.0-6 | pbapply_1.7-0 | cluster_2.1.2 |
| fs_1.5.2 | magrittr_2.0.3 | sna_2.7 |
| data.table_1.14.2 | openxlsx_4.2.5.1 | reprex_2.0.2 |
| googledrive_2.0.0 | hms_1.1.2 | evaluate_0.16 |
| xtable_1.8-4 | XML_3.99-0.10 | jpeg_0.1-9 |
| readxl_1.4.1 | fastDummies_1.6.3 | gridExtra_2.3 |
| compiler_4.1.1 | crayon_1.5.1 | minqa_1.2.4 |
| htmltools_0.5.3 | mgcv_1.8-36 | corpcor_1.6.10 |
| tzdb_0.3.0 | expm_0.999-6 | RcppParallel_5.1.5 |
| lubridate_1.8.0 | DBI_1.1.3 | kutils_1.70 |
| dbplyr_2.2.1 | boot_1.3-28 | Matrix_1.4-1 |
| permute_0.9-7 | cli_3.6.0 | difR_5.1 |
| GPArotation_2022.10-2 | parallel_4.1.1 | pkgconfig_2.0.3 |
| sem_3.1-15 | foreign_0.8-81 | xml2_1.3.3 |
| roxygen2_7.2.1 | pbivnorm_0.6.0 | svglite_2.1.0 |
| bslib_0.4.0 | admisc_0.29 | webshot_0.5.3 |
| rvest_1.0.3 | digest_0.6.29 | vegan_2.6-4 |
| rmarkdown_2.16 | cellranger_1.1.0 | htmlTable_2.4.1 |
| Deriv_4.1.3 | lisrelToR_0.1.5 | gtools_3.9.3 |
| nloptr_2.0.3 | lifecycle_1.0.3 | nlme_3.1-152 |
| glasso_1.11 | jsonlite_1.8.0 | Rhdf5lib_1.14.2 |
| network_1.17.2 | viridisLite_0.4.1 | fansi_1.0.4 |
| pillar_1.8.1 | GGally_2.1.2 | fastmap_1.1.0 |
| httr_1.4.4 | glue_1.6.2 | zip_2.2.1 |
| fdrtool_1.2.17 | png_0.1-7 | stringi_1.7.12 |
| sass_0.4.2 | latticeExtra_0.6-30 | dcurver_0.9.2 |
